# Development of Human Carbonic Anhydrase II Heterobifunctional Degraders

**DOI:** 10.1021/acs.jmedchem.2c01843

**Published:** 2023-02-03

**Authors:** Conor B. O’Herin, Yuta W. Moriuchi, Troy A. Bemis, Alysia J. Kohlbrand, Michael D. Burkart, Seth M. Cohen

**Affiliations:** Department of Chemistry and Biochemistry, University of California, La Jolla, California 92093, United States

## Abstract

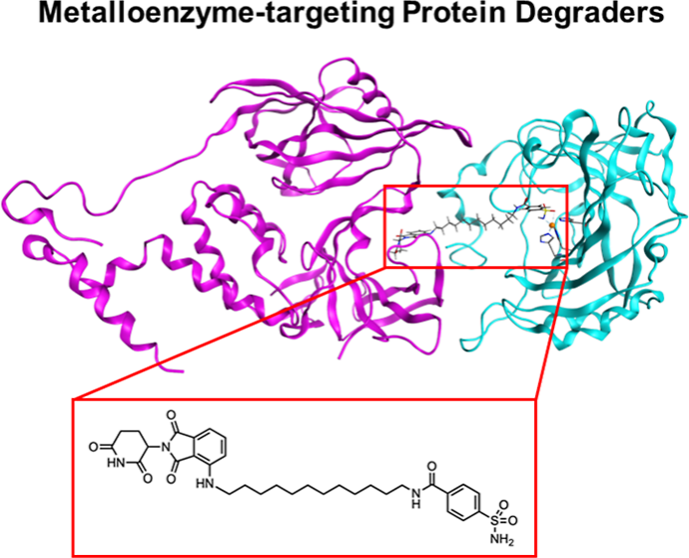

Human carbonic anhydrase II (hCAII) is a metalloenzyme essential to critical physiological processes in the body. hCA inhibitors are used clinically for the treatment of indications ranging from glaucoma to epilepsy. Targeted protein degraders have emerged as a promising means of inducing the degradation of disease-implicated proteins by using the endogenous quality control mechanisms of a cell. Here, a series of heterobifunctional degrader candidates targeting hCAII were developed from a simple aryl sulfonamide fragment. Degrader candidates were functionalized to produce either cereblon E3 ubiquitin ligase (CRBN) recruiting proteolysis targeting chimeras (PROTACs) or adamantyl-based hydrophobic tags (HyTs). Screens in HEK293 cells identified two PROTAC small-molecule degraders of hCA. Optimization of linker length and composition yielded a degrader with sub-nanomolar potency and sustained depletion of hCAII over prolonged treatments. Mechanistic studies suggest that this optimized degrader depletes hCAII through the same mechanism as previously reported CRBN-recruiting heterobifunctional degraders.

## Introduction

Human carbonic anhydrases (hCAs) are a family of Zn^2+^-dependent metalloenzymes that catalyze the reversible interconversion of CO_2_ to bicarbonate, an equilibrium essential to physiological processes such as respiration,^1^ pH balance,^2^ ion exchange,^3^ and bone readsorption.^4^ To date, there are 16 known hCA isoforms that vary in their activity, expression level, tissue distribution, and subcellular localization.^5,6^ In catalytic isoforms of hCA, the active site is located at the base of an ∼15 Å deep conical cleft,^7^ which is divided into distinct hydrophilic and hydrophobic regions that facilitate the transport of the bicarbonate ion and neutral carbon dioxide.^8^ The catalytic domain contains a Zn^2+^ ion coordinated to three histidine residues and a nucleophilic hydroxide in a tetrahedral coordination geometry. CO_2_ binds to a nearby hydrophobic pocket, and an adjacent threonine residue (Thr199) accepts a hydrogen bond from the Zn^2+^-coordinated hydroxide to orient it for the nucleophilic attack, leading to the formation of bicarbonate.

**Figure 1:**
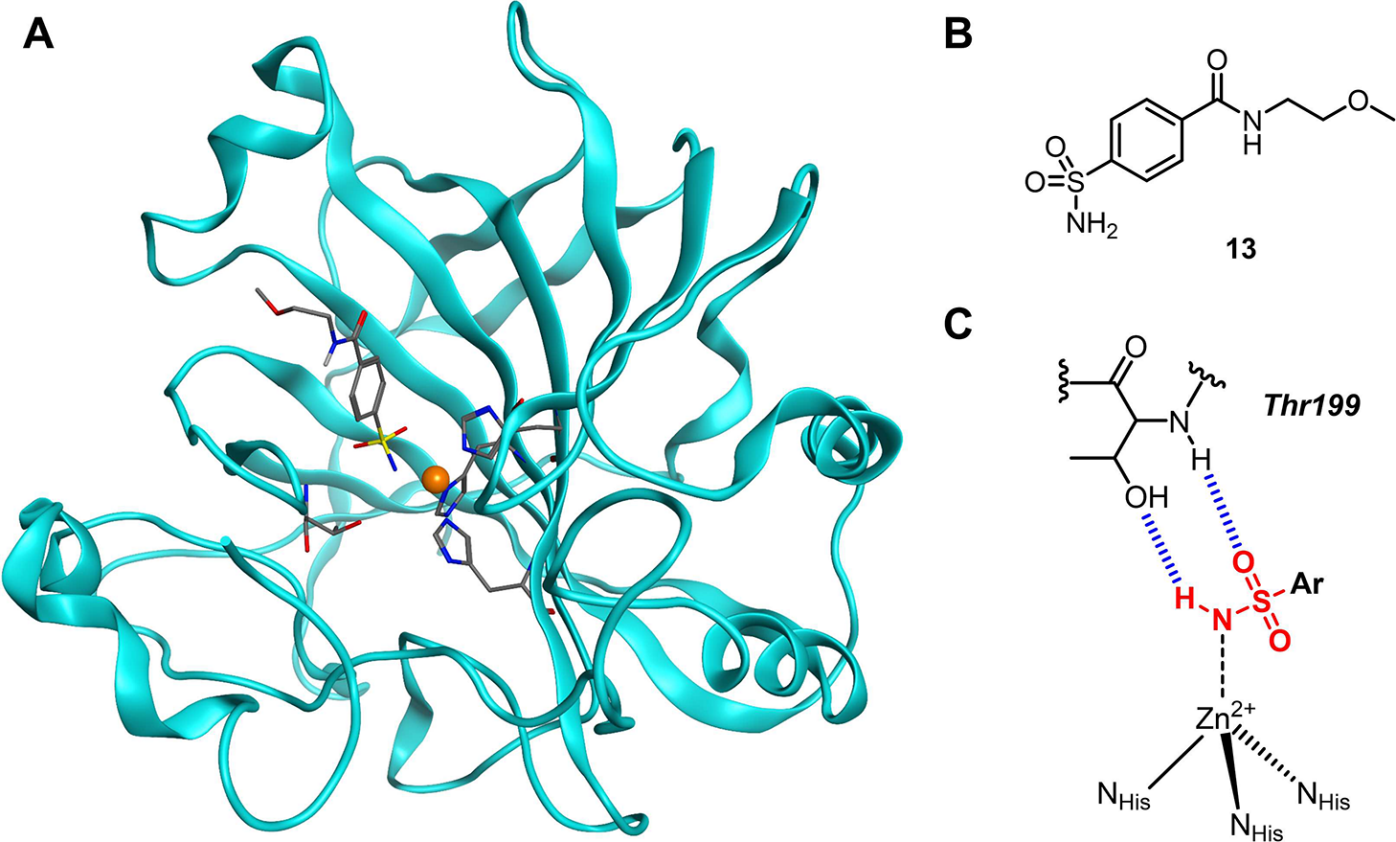
Representations of aryl sulfonamide inhibitors bound to hCAII. (A) Structure of hCAII bound to an aryl sulfonamide inhibitor (compound **13**, this work). The protein is represented as ribbons (cyan), the Zn^2+^ ion as an orange sphere, and the inhibitor, Zn^2+^-coordinating residues, and Thr199 as sticks (colored by atom). (B) Chemical structure of aryl sulfonamide inhibitor **13**. (C) Binding mode of an aryl sulfonamide coordinated to the catalytic Zn^2+^ ion of hCAII. The sulfonamide binding moiety is highlighted in red, and the hydrogen bonds to the adjacent Thr199 residue are marked as blue hashes.

Given their crucial role in important physiological processes, hCAs have been explored as pharmaceutical targets and biomarkers for a variety of diseases.^9,10^ Shortly following the discovery of carbonic anhydrases,^11^ aryl sulfonamides were identified as privileged and potent inhibitors^12^ of these enzymes and have since been the basis of several FDA-approved hCA-targeting drugs.^13^ Upon binding of these inhibitors, the metal bound hydroxide ion is displaced by the deprotonated nitrogen atom of the aryl sulfonamide moiety and the interaction is stabilized by strong hydrogen bonding with the nearby Thr199 residue (Figure 1). Among the hCA isoforms, human carbonic anhydrase II (hCAII) is abundantly expressed and found to be broadly distributed in human tissues.^5^ As such, it has been explored as a target in clinical treatments for indications including altitude sickness, edema, epilepsy, and glaucoma.^9^ Despite widespread clinical application for the treatment of glaucoma,^14^ none of the first or second generation FDA-approved hCA inhibitors are isoform selective. Ongoing efforts have tried to identify hCAII selective inhibitors that minimize off-target, systemic activities to decrease side effects and improve efficacy.^15−17^ Additionally, recent studies have elucidated the important non-catalytic proton shuttling function of hCAII, which supports lactate transport in cancer cells^18^ through its interaction with and subsequent activation of monocarboxylate transporter isozyme 1.^19,20^ Such activity is the consequence of residues on the surface of the enzyme and is thereby independent of enzymatic activity or inhibition. This observation prompted interest in exploring approaches beyond conventional small-molecule inhibitors to target and modulate the levels of this enzyme within targeted cells.

One alternative approach to modulating protein function that has gathered interest over the past two decades is targeted protein degraders (TPDs). These molecules have an “event-driven” pharmacology that is distinct from the “occupancy-driven” pharmacology of traditional inhibitors and is dependent neither on enzymatic inhibition nor on prolonged active site occupancy. Instead, TPDs recruit endogenous, cellular quality control mechanisms to a protein of interest (POI) in a transient interaction that selectively labels the target for degradation to eliminate its function.^21^ Heterobifunctional degraders are a subset of TPDs that achieve this distinct mechanism of action (MoA) by pairing a POI targeting ligand to a host recruiting ligand through a chemical linker. This molecular design allows degraders to bind to a POI and display moieties which recruit cellular quality control mechanisms to the target protein for degradation.

Access to rapid and reversible in vivo protein silencing via TPDs offers a new approach to both eliminate protein activity for therapeutics^22^ and achieve chemical knockdown in studies of protein function.^23,24^ Moreover, the characteristic, “event-driven” pharmacology found in TPDs offers exciting benefits over the activity of traditional small-molecule inhibitors. Iterative rounds of degradation by a single TPD can yield sub-stoichiometric activity,^25^ providing the potential for lower dosages and correspondingly diminished off-target toxicity. Sustained degradation can also circumvent the compensatory feedback activation observed upon treatment with small-molecule inhibitors in certain signaling pathways^26^ and provide durable suppression of proteins with low resynthesis rates.

Since 2019, a variety of heterobifunctional degraders have entered clinical trials for FDA approval. The two most advanced candidates from Arvinas (ARV-110 and ARV-471) entered Phase II trials in 2021,^27,28^ suggesting a positive future for TPDs. Current efforts are underway to identify target classes that are best suited to overcome the limitations of conventional inhibitors through the unique mechanism of TPDs.^22^ One of the potential benefits of the transient nature of degrader–target interactions is the need for targeting ligands of only moderate affinity.^25^ Metalloenzymes, such as hCAII, are a target class uniquely poised to take advantage of this facet of degradation because of the ability of their metal active sites to form strong, yet reversible,^29^ coordinate covalent bonds with fragment-sized metal binding pharmacophores (MBPs). This distinctive binding strategy makes metalloenzymes attractive targets for heterobifunctional degrader designs because of the potential to quickly generate a large collection of degrader candidates from relatively simple MBP fragments. Specific interactions at the ternary complex interface can also introduce novel selectivity beyond that of the degrader targeting group,^30−32^ allowing for the generation of specific degraders from less specific MBPs. This would provide an opportunity to overcome limitations in inhibitor selectivity caused by conserved active sites across and within metalloenzyme families.

At present, the majority of reported heterobifunctional degraders utilize the best available small-molecule inhibitors as targeting groups because of their well-established potency and selectivity. Previously reported studies have shown that both advanced inhibitors and smaller molecules containing metal-binding moieties can be used as targeting groups in the development of Zn^2+^-dependent histone deacetylase (HDAC) heterobifunctional degraders.^31,33−36^ It is the intention of this study to explore the well-studied hCAII enzyme to evaluate the potential of a ground-up TPD discovery approach that utilizes small, simple MBP fragments as ligands for the POI. Here, the privileged features of the metalloenzyme target class and the unique mechanism of heterobifunctional degraders are leveraged to promote the degradation of the target metalloenzyme.

## Results and Discussion

To evaluate an MBP-based TPD design approach with hCAII, the *p*-sulfamoyl benzamide MBP fragment was chosen because of its simplicity, well-characterized binding to the Zn^2+^ active site, and amenability to linker ligation.^7^ Two recruiting groups were selected from the variety of the distinct degradation pathways identified in heterobifunctional degraders.^37^ The first pathway pursued belongs to proteolysis targeting chimeras (PROTACs), which have moieties that bind both the POI and one of over 600 endogenous E3 ubiquitin ligases of the ubiquitin-proteasome system (UPS).^38^ When all three components (POI, TPD, and E3 ligase) are simultaneously bound in a “productive” ternary complex, surface lysine residues on the POI are ubiquitinated. Following the disassembly of this complex, the degrader is free to repeat this cycle in an iterative fashion, while the ubiquitin-labeled POI is degraded by the proteasome.^39,40^ The second pathway explored belongs to hydrophobic tags (HyTs), which display bulky hydrophobic groups at the POI surface. These hydrophobic moieties are believed to cause misfolding at the protein surface or be recognized as the interior residues of misfolded proteins to be subsequently delivered to the proteasome for degradation by cellular chaperone proteins.^41^

Ten initial heterobifunctional degrader candidates were synthesized by joining the *p*-sulfamoyl benzamide moiety to either the cereblon E3 ubiquitin ligase (CRBN) recruiting pomalidomide ligand (PROTAC candidates **1–5**) or 1-adamantaneacetic acid HyT (candidates **6–10**) using 1 of 5 linkers. A variety of linkers were screened because of the well-established impact linker length and composition have on heterobifunctional degrader activity,^42^ due to the importance of the linker structure in PROTAC ternary complex formation and HyT surface display.

**Scheme 1:**
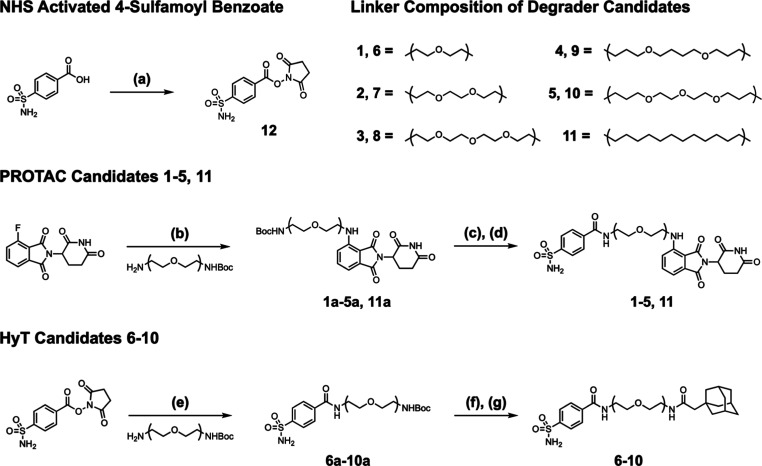
Synthesis of Degrader Candidates **1–11** Reaction conditions: (a) NHS, EDC, DMF, 0 °C to rt, 18 h, 50–81%; (b) DIPEA, DMSO, 90 °C, 18 h, 31–65%; (c) TFA, DCM, rt, 2 h; (d) **12**, DMF, rt, 18 h, 22–45%; (e) **12**, DMF, rt, 18 h, 46–84%; (f) TFA, DCM, rt, 2 h; (g) DIPEA, HATU, 1-adamantane acetic acid, DMF, rt, 18 h, 24–81%.

The general synthetic route for these candidate hCAII degraders is summarized in Scheme 1. First, NHS-activated 4-sulfamoyl benzoate (**12**) was synthesized through coupling of NHS to appropriately substituted benzoic acids using EDC.^43^ PROTAC candidates were prepared by nucleophilic aromatic substitution between racemic 4-fluorothalidomide and mono-Boc protected diamine linkers to generate intermediates **1a–5a**.^44^ Subsequent deprotection of the Boc group using trifluoroacetic acid (TFA) produced the triflate salts of terminal amines, which were then coupled with the NHS-activated, 4-sulfamoyl benzoate to yield completed PROTAC candidates **1–5**.^45^ HyT candidates were synthesized by coupling mono-Boc protected diamine linkers to NHS-activated, 4-sulfamoyl benzoate to give intermediates **6a–10a**. Deprotection of the Boc group using TFA generated the triflate salts of terminal amines, which were then coupled to 1-adamantaneacetic acid using HATU to yield completed HyT candidates **6–10**.^44^

To verify that the *p*-sulfamoyl benzamide MBP retained its binding following ligation with the linker and bulky recruiting group moieties, the inhibition activity of each degrader candidate was determined using a colorimetric *p*-nitrophenol acetate hCAII activity assay.^46^ In short, recombinantly expressed hCAII was preincubated with the degrader candidate before the addition of *p*-nitrophenyl acetate, a substrate that upon enzymatic cleavage leads to acetic acid and *p*-nitrophenolate. By monitoring the absorbance of *p*-nitrophenolate at 405 nm over 15 min, the degree of inhibition was determined by comparison of substrate hydrolysis in inhibited and uninhibited assay wells. Inhibition activity was reported as half maximal inhibitory concentration (IC_50_) values with 95% confidence interval. IC_50_ values of degrader candidates **1–10** and test compound **13** were compared to the FDA-approved hCA inhibitor, acetazolamide (**AAZ**). All measured values fell below an IC_50_ value of 100 nM (Table S1), with values of **AAZ**, **4**, **5**, **7**, and **10** falling below the detection limit of the assay (≤20 nM at an enzyme concentration of 40 nM). These results demonstrate that neither the para-linker attachment position nor the steric bulk of linked recruiting groups substantially impacted in vitro hCAII binding. Observed differences in candidate binding affinity can be attributed to the varying peripheral contacts of different linker and recruiting groups with the active site channel and protein surface and is a well-documented SAR in the hCA inhibitor literature.^47^

**Figure 2:**
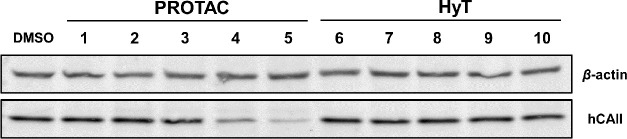
Single-point activity screen of degrader candidates **1**–**10**. Western blot analysis of HEK293 cells treated with degrader candidate (5 μM final concentration) or vehicle (DMSO) for 24 h. PROTAC degrader candidates **1–3** and HyT degrader candidates **6–10** showed no observable activity under the conditions of this single-point screen and were not studied further. Degraders **4** and **5** were identified as active from this screen. Full images of the western blots are provided within the Supporting Information (Figure S1).

Following the confirmation of candidate hCAII’s in vitro activity, candidate degraders **1–10** were evaluated for their degradation of hCAII using HEK293 cells, a line known to abundantly express hCAII.^48^ To obtain a preliminary evaluation of the activity of these compounds, an initial single-point screen was performed with HEK293 cells dosed with individual degrader candidates at a final concentration of 5 μM for 24 h prior to cell lysis. The relative abundance of hCAII in whole cell lysates was then evaluated via western blot analysis (see the Experimental Section for details). From this experiment, the PROTAC degrader candidates with the two longest linkers (compounds **4** and **5**) were shown to decrease the percent hCAII relative to the β-actin (loading) and dimethyl sulfoxide (DMSO) vehicle controls (Figure 2). Both active degraders **4** and **5** were selected for further characterization, although **5** demonstrated higher activity (85% protein degradation) than **4** (64% protein degradation) when quantified by western blot analysis (Figure 2).

**Figure 3:**
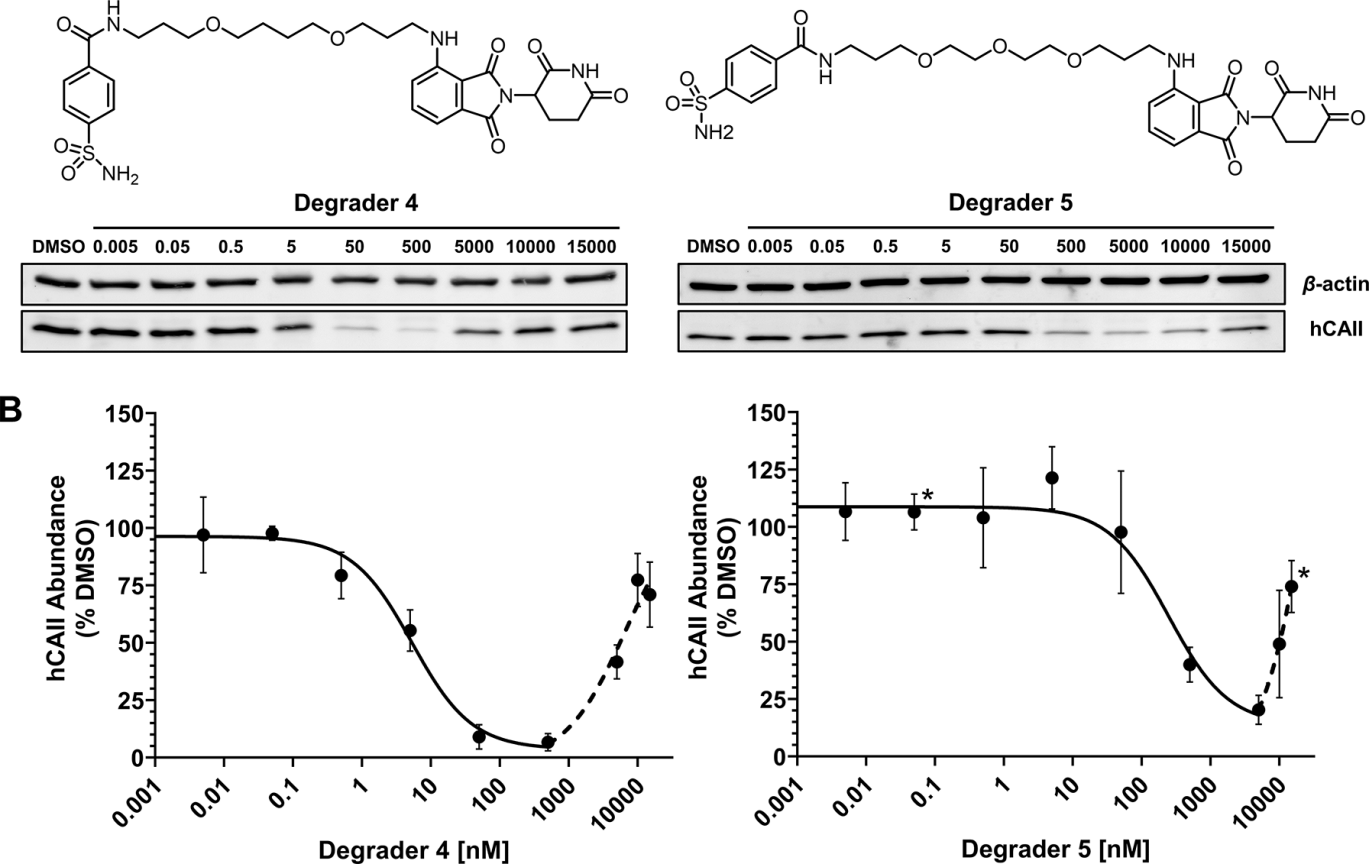
Dose-dependent degradation of hCAII by **4** and **5**. (A) Western blot images of HEK293 cells dosed for 24 h with degrader **4** (left) or **5** (right) at concentrations of 5 pM to 15 μM. (B) Dose response curves of hCAII abundance as a percent of DMSO vehicle for **4** (left) or **5** (right). Band intensities of blots from three biological replicates were quantified using ImageJ software (Experimental Section). Data were normalized to a vehicle-treated (DMSO) group and plotted as a sigmoidal curve. Nonlinear fitting of log([inhibitor]) vs response (three parameters) was generated for **4** with *R*^2^ = 0.955 and **5** with *R*^2^ = 0.855 in GraphPad Prism. Points marked with an asterisk indicate only two biological replicates. In all other cases, error bars represent the standard deviation of triplicate experiments. Full images of the western blots are provided in the Supporting Information (Figures S1 and S3).

To evaluate the potency of the active compounds identified in the candidate screen, nine-point dose response degradation assays were performed using degraders **4** and **5**. Three biological replicates of HEK293 cells were dosed in parallel with **4** or **5** at concentrations ranging from 5 pM to 15 μM for 24 h prior to lysis. Western blot analysis and quantification of blot intensity showed both **4** and **5** degrading hCAII in a dose-dependent manner (Figures 3A and S3). Of the two active degraders, **4** (maximum degradation at 500 nM) showed greater activity in the dose response experiment than **5** (maximum degradation at 5 μM). In concentrations above their maximum activity, both compounds exhibited a well-known attenuation in target degradation known as the “hook effect”.^49^ This phenomenon can be attributed to the binary complexes of degrader-POI and degrader-E3 ligase outcompeting the POI-degrader-E3 ligase ternary complex at high degrader concentrations. The observed discrepancy in comparative potencies of **4** and **5** between the single-point candidate screen and dose response experiments can, in part, be attributed to the “hooking” of **5** at a 5 μM dose. The half-maximal degradation (DC_50_ value reported as a 95% confidence interval) and maximum percentage of degradation (*D*_Max_) of each active degrader were determined using a least squares sigmoidal fitting of the degradation activity of dosing concentrations below the observable “hook effect” (Figure 2B). Degrader **4** was determined to possess a DC_50_ value of 5 ± 3 nM with a *D*_Max_ of 96%, and degrader **5** was determined to possess a DC_50_ value of 245 ± 246 nM with a *D*_Max_ of 86%.

**Figure 4:**
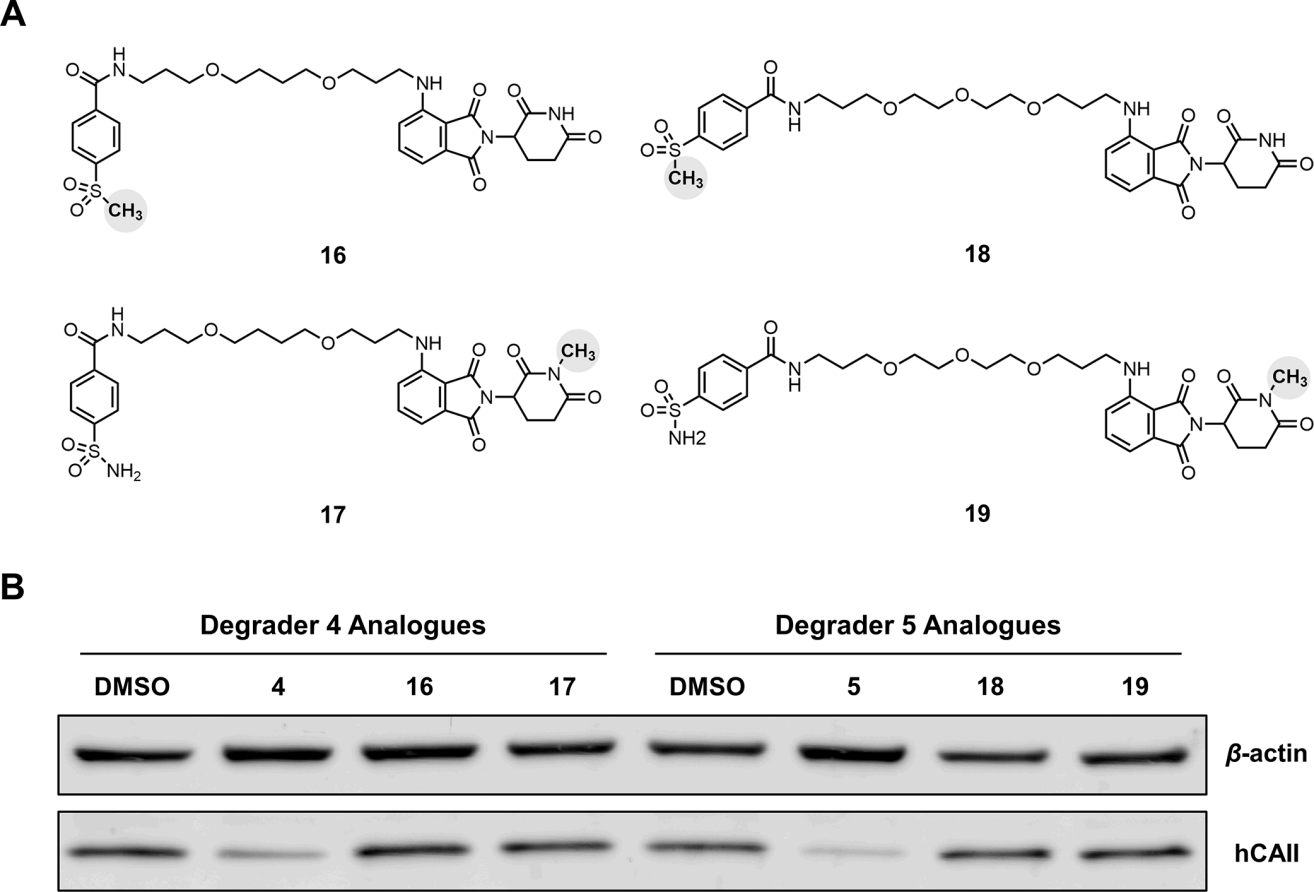
Inactivated mechanistic controls for degraders **4** and **5**. (A) Analogues of **4** (**16** and **17**, left) and **5** (**18** and **19**, right) with inactivating methyl groups (bold, in shaded spheres). (B) Western blot analysis of HEK293 cells treated with degraders **4** and **5** (5 μM final concentration), inactivated analogues **16–19** (5 μM final concentration), or vehicle (DMSO) for 24 h. Full images of the western blots are provided in the Supporting Information (Figure S1).

Next, analogues of each degrader were synthesized with inactivated binding motifs to either hCAII, **16** and **18**, or CRBN, **17** and **19** (Figure 4) to confirm that hCAII depletion produced by treatment with **4** or **5** was the consequence of a PROTAC mechanism requiring ternary complex formation. With **16** and **18**, binding to hCAII was blocked by replacing the essential sulfonamide moiety with a mesyl group. For **17** and **19**, binding to CRBN was blocked by N-methylation of the glutarimide nitrogen of the pomalidomide moiety. Compounds **16–19** were synthesized in a similar fashion to PROTAC degrader candidates **4** and **5** using an N-methylated 4-fluorothalidomide (**14**). Western blot analysis of HEK293 cells treated with inactivated degrader analogues **16–19** at final concentrations of 5 μM for 24 h prior to cell lysis showed no observable degradation in comparison to a vehicle (DMSO) control, unlike those treated with either degrader **4** or **5** (Figure 4). This result suggests that the hCAII degradation observed in treatments with **4** and **5** is induced by the productive ternary complex formation expected from the established mechanism of PROTAC action.

**Figure 5:**
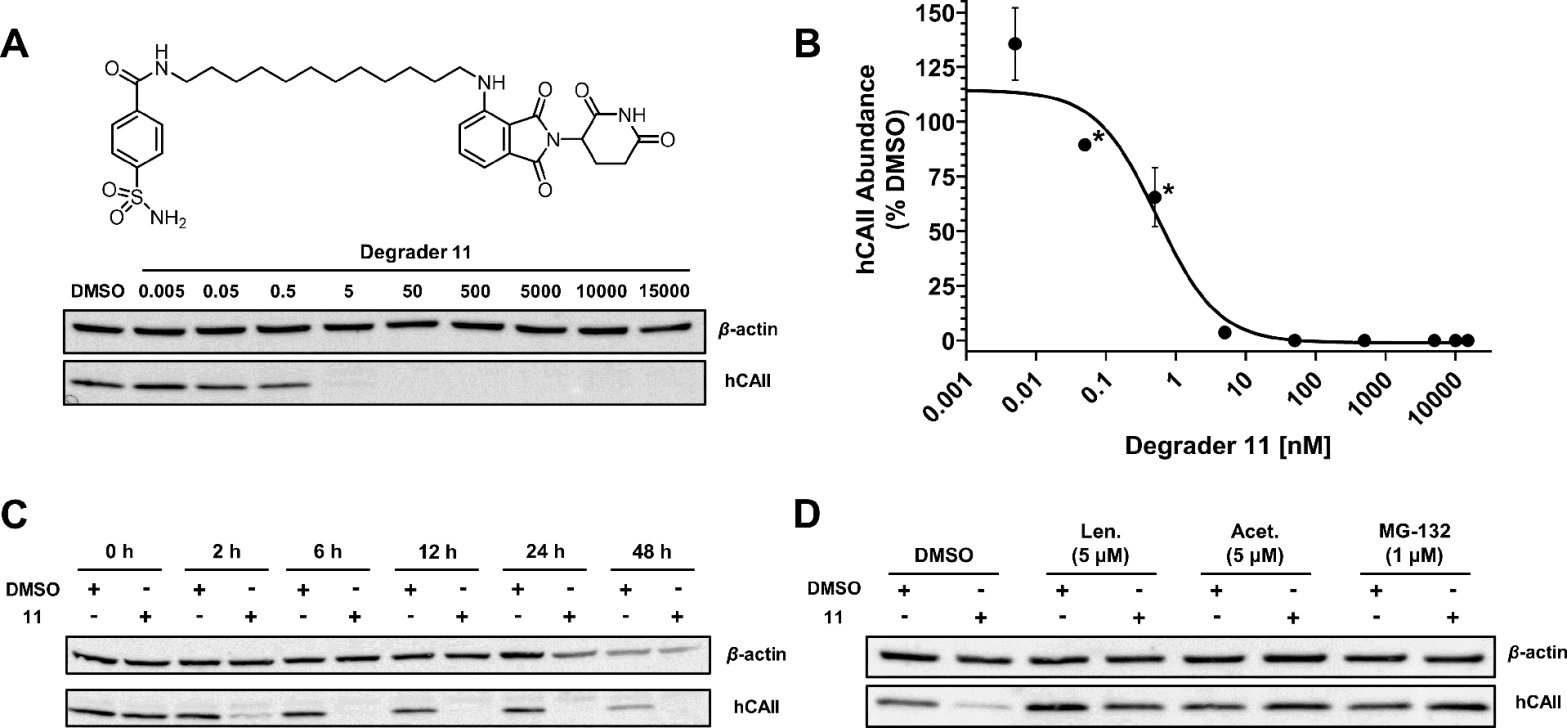
Compound **11** induces dose-dependent degradation of hCAII through a mechanism consistent with the heterobifunctional degrader activity. (A) Western blot images of HEK293 cells dosed with degrader **11** at final concentrations of 5 pM to 15 μM for 24 h. (B) Dose response curve of hCAII abundance as a percent of DMSO vehicle for **11**. The band intensity of the dose response blots of three biological replicates was quantified using the ImageJ software (Experimental Section). Data were normalized to a vehicle-treated (DMSO) group and were plotted as a sigmoidal curve. Nonlinear fitting of log([inhibitor]) vs response (three parameters) was generated for **11** in GraphPad Prism with *R*^2^ = 0.952. Points indicated by an asterisk indicate the plotting of only two biological replicates. In all other cases, error bars represent the standard deviation of triplicate experiments. (C) Time-course change in hCAII expression with treatment of **11** at 50 nM for 0–48 h. (D) Mechanistic studies of **11**. Western blot analysis of HEK293 cells pretreated with vehicle (DMSO), CRBN inhibitor lenalidomide, hCAII inhibitor acetazolamide, or proteasome inhibitor MG132 for 1 h, followed by treatment with 50 nM of **11** or vehicle (DMSO) for 6 h. Full images of the western blots are provided in the Supporting Information (Figures S2 and S3).

Following the identification of **4** as the most active lead compound and confirmation of the PROTAC-style mechanism of hCAII degradation, consideration was given to the development of a more potent degrader using the limited SAR observed in the degrader candidate screen. Given the inactivity of **1–3** and increased potency of **4** over **5**, a 12-atom linker appeared to be a preferred length for favorable ternary complex formation in the heterobifunctional degraders tested. To further examine the role of linker length and composition, we explored hydrocarbon linkers for their ability to improve degrader lipophilicity and membrane permeability without impacting the favorable ternary complex formation. Compound **11**, an analogue of degrader **4** with a purely aliphatic linker, was synthesized and evaluated (Figure 5) using a nine-point dose response degradation assay with dosages ranging from 5 pM to 15 μM administered 24 h before lysis. Western blot analysis and quantification of three biological replicates identified the degradation of hCAII at concentrations of **11** as low as 50 pM and achieved near-complete hCAII degradation at concentrations of 5 nM (Figures 5A and S3). Moreover, in contrast to the activity of **4** and **5**, dosages as high as 15 μM maintained complete degradation of hCAII and exhibited no appreciable “hook effect”. Least squares sigmoidal fitting of the degradation activity of hCAII in comparison to vehicle (DMSO) revealed a DC_50_ value of 0.5 ± 0.3 nM and *D*_Max_ of 100%, a 10-fold increase in activity over **4**.

To determine the efficiency of **11**, hCAII abundance was evaluated in HEK293 cells that were dosed with 50 nM of **11** over 48 h. Depletion of hCAII relative to vehicle (DMSO) began within 2 h and reached a maximal effect by 6 h (Figure 5c). Sustained degradation was also observed for the entirety of the 48 h tested. Based on the results of this time-course dosing experiment, it appears that **11** begins degrading hCAII quickly after exposure to the cell and maintains prolonged activity after initial dosing.

Competition experiments were performed to verify that the degradation activity of **11** was the result of a PROTAC-style mechanism requiring concurrent degrader engagement with both hCAII and CRBN. Following 1 h of preincubation with 5 μM of either hCAII-binding acetazolamide or CRBN-binding lenalidomide, HEK293 cells were treated with 50 nM of compound **11** for 6 h. Lenalidomide and acetazolamide pre-treatments dosed with **11** showed no degradation of hCAII compared to their vehicle (DMSO) controls (Figure 5d), suggesting that **11** must engage with both hCAII and CRBN to achieve degradation. To confirm that the degradation activity of compound **11** was UPS dependent, as is typical of CRBN-recruiting heterobifunctional degraders, HEK293 cells were pre-treated with the proteasome inhibitor MG132^50^ before treatment with **11**. Pre-treating HEK293 cells with 1 μM of MG-132 for 1 h before dosing with 50 nM of **11** for 6 h resulted in a complete loss of hCAII degradation activity (Figure 5d), confirming that the observed activity was proteasome dependent. Taken together, these results strongly suggest that **11** degrades hCAII through the same mechanism as previously reported for CRBN-recruiting heterobifunctional degraders and does so with a high level of efficiency.

To understand the binding mode of active degraders with hCAII and CRBN, the structures of degraders co-crystallized with recombinantly expressed hCAII and models of ternary complexes of CRBN·degrader·hCAII with molecular docking were determined. Crystallographic conditions were initially optimized with *N*-(2-methoxyethyl)-4-sulfamoylbenzamide (**13**), a test compound composed of the *p*-sulfamoyl benzamide moiety attached to a methoxyethanamine linker but no CRBN recruiting moiety (Figure 1). Crystallographic analysis found that **13** bound to the Zn^2+^ active site in a canonical fashion via its aryl sulfonamide moiety and its linker extending up the active site cleft toward the enzyme surface (Figure 1).

Crystal structures obtained of complete degrader candidates **1**, **2**, and **4** bound to hCAII demonstrated similar binding configurations in the active site but had a diminishing resolution as the linker extended through the active site channel (Figure S4). The cocrystal structure of **1** and hCAII showed well-ordered density through the linker and indicated the hydrolysis of the phthalimide and glutarimide ring of the pomalidomide recruiting group at the surface of the active site channel. The density of the cocrystal structure of **2** and hCAII demonstrated well-ordered binding up to the second oxygen of the PEG linker and indicated that the glutarimide ring of the pomalidomide group has undergone hydrolysis at the surface of the enzyme. The cocrystal structure of degrader **4** and hCAII had well-ordered density through the first two methylene units of the linker but insufficient density to resolve the remainder of the linker or the pomalidomide functionality, suggesting disordered binding of the recruiting group at the enzyme surface. This poor resolution at the hCAII surface is unsurprising, given the absence of pomalidomide binding interactions and presumed disordered surface display of the recruiting group. Structures of the test compound and degrader candidates confirmed the expected binding of the targeting groups to the Zn^2+^ metal ion active site and provide a basis for ternary complex modeling.

**Figure 6:**
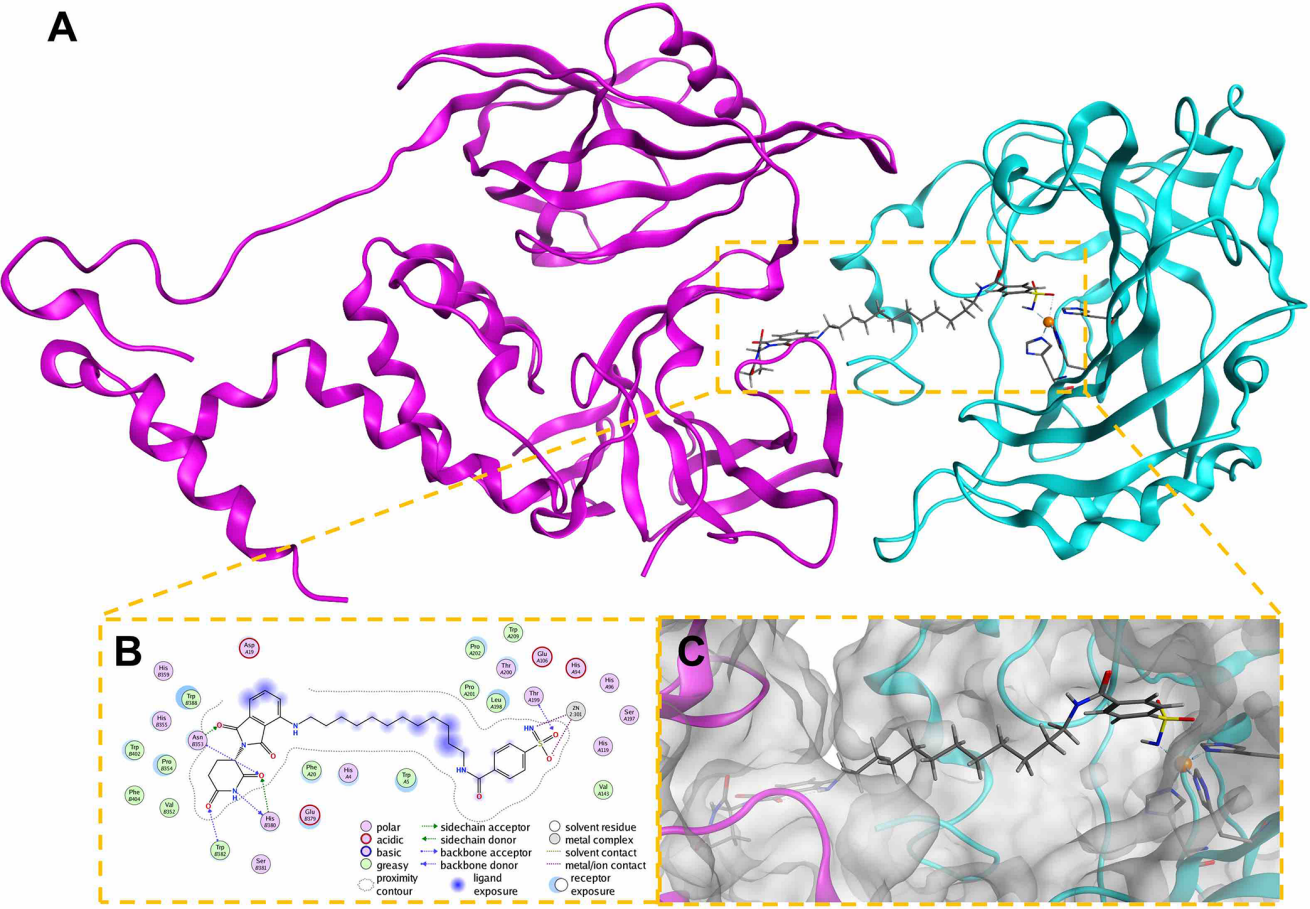
Predicted binding pose of the CRBN·**11**·hCAII ternary complex (CRBN PDB: 4CI3, hCAII PDB: 8EMU). (A) Structure of the CRBN·**11**·hCAII complex with CRBN in magenta, hCAII in cyan, and the structure of **11** in gray. (B) Ligand interaction map of **11** showing interactions in CRBN and hCAII binding pockets and diagram legend. (C) Close-up view of degrader **11** binding pose and placement in protein surface maps.

Complexes of hCAII·**11**·CRBN were modeled in Molecular Operating Environment (MOE) using a method able to successfully generate poses resembling reported ternary-complex structures of active degraders.^51^ Using the solved **13**·hCAII complex (PDB: 8EMU) and previously reported structure of pomalidomide·CRBN (PDB: 4CI3),^52^ an ensemble of ternary complexes of degrader **11** with hCAII and CRBN was successfully modeled. The ternary complex model possessing the lowest total forcefield interaction energy between **11** and both proteins presents the degrader comfortably spanning the two protein active sites with a relatively straight linker pose while maintaining appropriate interactions in the binding pockets of both proteins (Figure 6).

## Conclusions

In summary, we have developed small-molecule degraders of hCAII, PROTACs **4** and **5**, using a *p*-sulfamoyl benzamide targeting group and a pomalidomide E3 ligase recruiter. These molecules were identified from a collection of ten candidates generated using a simple, two-step synthesis. In evaluations of degrader potency, **5** was found to have a greater potency than **4**, with a DC_50_ value of 5 ± 3 nM and *D*_Max_ of 96%. Linker optimization of **5** led to the development of **11**, a degrader displaying a DC_50_ value of 0.5 ± 0.3 nM and *D*_Max_ of 100%, which showed no appreciable “hook-effect” at doses up to 15 μM. Further characterization showed **11** depleting hCAII in as little as 2 h and sustaining degradation activity up to 48 h. Mechanistic studies demonstrated that **11** requires ternary complex formation and proteasomal function to degrade hCAII, suggesting a MoA consistent with previously reported PROTACs.^53^ These results highlight the viability of an MBP-derived degrader development approach in metalloenzyme targets and offer a potent, time-dependent approach to hCAII silencing using small molecules. Future studies of these degraders will explore their potential for isozyme selectivity and activity across different CRBN containing cell lines.

## Experimental Section

### General Synthetic Experimental Details

All solvents and reagents were obtained from commercial sources (Fisher Scientific, VWR Scientific, Sigma-Aldrich, Combi-Blocks) and used without further purification, unless otherwise noted. All reactions were performed under a positive pressure of nitrogen or argon in flame-dried glassware. Reactions were monitored using glass-backed TLC plates impregnated with a fluorescent indicator absorbing at 254 nm. Silica gel chromatography was performed on a CombiFlash R_f_ Teledyne ISCO system using hexane, EtOAc, dichloromethane (DCM), or MeOH as eluents. C_18_ reverse phase chromatography was performed on the same instrument using 0.1% formic acid in methanol, acetonitrile, or water as an eluent. ^1^H and ^13^C NMR spectra were obtained on a Varian (400 MHz) spectrometer, Jeol (400 MHz) spectrometer, or a VX (500 MHz) equipped with XSens cold probe (Varian) spectrometer in the Department of Chemistry and Biochemistry at UC San Diego. The purity of all compounds used in assays and cell studies was determined to be ≥95% by HPLC analysis. Mass spectrometry was performed at the UC San Diego Molecular Mass Spectrometry Facility. HRMS analysis was performed using an Agilent 6230 Accurate-Mass LC-TOFMS located at the UC San Diego Molecular Mass Spectrometry Facility.

### Synthetic Procedure and Compound Characterization

#### General Procedure I: Preparation of Boc-Protected Linker Pomalidomide Conjugates **1a–5a** & **11a**

4-Fluoro-thalidomide (10 mmol) was added to a solution of appropriate mono-Boc protected diamine (10 mmol) and DIPEA (40 mmol) in dry DMSO (10 mL), and the resulting solution was stirred at 90 °C overnight. After cooling to rt, the greenish mixture was dissolved in half-saturated brine (100 mL) and extracted with EtOAC (3 × 50 mL). The combined organic layers were washed with sat. NH_4_Cl, 5% LiCl, and brine (50 mL each), dried over MgSO_4_, filtered, and concentrated in vacuo.

#### General Procedure II: Preparation of PROTAC Degrader Candidates **1–5** and **11**

TFA (130 mmol) was added to a stirring solution of appropriate Boc-protected linker pomalidomide conjugate (500 μmol) in dry DCM (10 mL). After 2 h of stirring at rt, the solvent was removed in vacuo, and the resulting oil was coevaporated with dry DCM (3 × 8 mL). The oily residue was further dried under a high vacuum and used in the next reaction without further purification.

The resultant linker pomalidomide conjugate and NHS-activated 4-sulfamoyl benzoate **12** (1.1 mmol) were dissolved in dry DMF (2.5 mL). After 18 h of stirring at rt, the solvent was removed in vacuo. The residue was then taken up in ethyl acetate (25 mL), poured into a 5% solution of sodium bicarbonate (10 mL), and extracted with ethyl acetate (3 × 25 mL). The combined organic layers were dried over sodium sulfate and filtered, and the solvent was removed in vacuo.

#### General Procedure III: Preparation of Boc-Protected Linker 4-Sulfamoyl Benzamide Conjugates **6a–10a**

NHS-activated 4-sulfamoyl benzoate **12** (550 μmol) and appropriate mono-Boc protected diamine (500 μmol) were dissolved in dry DMF (3.0 mL) at rt. After the solution was stirred for 18 h, the solvent was removed in vacuo. The residue was taken up in ethyl acetate (25 mL), poured into a 5% solution of sodium bicarbonate (10 mL), and extracted with ethyl acetate (3 × 25 mL). The combined organic layers were dried over MgSO_4_ and filtered, and the solvent was removed in vacuo.

#### General Procedure IV: Preparation of HyT Degrader Candidates **6–10**

TFA (130 mmol) was added to a stirring solution of the appropriate Boc-protected linker 4-sulfamoyl benzamide conjugate (500 μmol) in dry DCM (10 mL). After 2 h of stirring at rt, the solvent was removed in vacuo and the resulting oil was coevaporated with dry DCM (3 × 10 mL). The oily residue was further dried under a high vacuum and used in the next reaction without further purification.

DIPEA (2.0 mmol) and HATU (550 μmol) were added to a stirring solution of 1-adamantaneacetic acid (500 μmol) in dry DMF (10 mL). After 5 min of stirring, a solution of the deprotected linker 4-sulfamoyl benzamide conjugate in dry DMF (10 mL) was added. After 18 h of stirring at rt, the solvent was removed in vacuo. The resultant residue was re-dissolved in half saturated brine (100 mL) and extracted EtOAc (3 × 50 mL). The combined organic layers were further washed with saturated NH_4_Cl solution, 5% LiCl solution, and brine (50 mL each). The combined organic layers were dried over MgSO_4_ and filtered, and the solvent was removed in vacuo.

#### *tert*-Butyl(2-(2-((2-(2,6-dioxopiperidin-3-yl)-1,3-dioxoisoindolin-4-yl)amino)ethoxy)ethyl)carbamate (**1a**)

This compound was prepared using General Procedure I and *tert*-butyl(2-(2-aminoethoxy)ethyl)carbamate (170 mg, 0.83 mmol). The crude product was purified by silica column chromatography (gradient of 30–70% EtOAc in Hex) to yield **1a** (242 mg, 65%) as a yellow oil. ^1^H NMR (400 MHz, acetone-*d*_6_): δ 9.95 (s, 1H), 7.57 (dd, *J* = 8.5, 7.1 Hz, 1H), 7.13 (d, *J* = 8.6 Hz, 1H), 7.04 (d, *J* = 7.0 Hz, 1H), 6.61 (t, *J* = 5.7 Hz, 1H), 5.93 (t, *J* = 5.9 Hz, 1H), 5.13–5.02 (m, 1H), 3.72 (t, *J* = 5.4 Hz, 2H), 3.54 (td, *J* = 5.6, 3.8 Hz, 4H), 3.26 (q, *J* = 5.8 Hz, 2H), 3.03–2.88 (m, 1H), 2.85–2.70 (m, 2H), 2.21 (ddt, *J* = 12.3, 5.4, 2.6 Hz, 1H), 1.38 (s, 9H). ^13^C NMR (101 MHz, acetone-*d*_6_): δ 172.8, 170.2, 170.2, 168.2, 156.6, 147.7, 136.9, 133.5, 117.8, 111.5, 111.0, 78.6, 70.5, 69.9, 49.8, 42.9, 40.9, 32.0, 28.6, 23.3. HRMS (ESI): calcd for [C_22_H_28_N_4_O_7_Na]^+^, 483.1850; measured, 483.1846.

#### *tert*-Butyl(2-(2-(2-((2-(2,6-dioxopiperidin-3-yl)-1,3-dioxoisoindolin-4-yl)amino)ethoxy)ethoxy)ethyl)carbamate (**2a**)

Compound **2a** was prepared using General Procedure I and *tert*-butyl(2-(2-(2-aminoethoxy)ethoxy)ethyl)carbamate (270 mg, 1.1 mmol). The crude product was purified by silica column chromatography (gradient of 30–90% EtOAc in Hex) to yield **2a** (198 mg, 36%) as a yellow oil. ^1^H NMR (400 MHz, acetone-*d*_6_): δ 9.96 (br s, 1H), 7.58 (dd, *J* = 8.4, 7.1 Hz, 1H), 7.12 (d, *J* = 8.6 Hz, 1H), 7.04 (d, *J* = 7.1 Hz, 1H), 6.62 (t, *J* = 5.6 Hz, 1H), 5.89 (s, 1H), 5.07 (dd, *J* = 12.6, 5.4 Hz, 1H), 3.74 (t, *J* = 5.3 Hz, 2H), 3.66–3.57 (m, 4H), 3.52 (m, 4H), 3.22 (q, *J* = 5.8 Hz, 2H), 3.03–2.88 (m, 1H), 2.85–2.69 (m, 2H), 2.21 (ddt, *J* = 12.4, 5.5, 2.7 Hz, 1H), 1.38 (s, 9H). ^13^C NMR (101 MHz, acetone-*d*_6_): δ 172.7, 170.3, 168.3, 156.6, 147.7, 136.9, 133.5, 117.8, 111.5, 111.0, 78.6, 71.1, 70.9, 70.7, 70.1, 49.8, 42.9, 41.0, 32.0, 28.6, 23.4. HRMS (ESI): calcd for [C_24_H_32_N_4_O_8_Na]^+^, 527.2112; measured, 527.2110.

#### *tert*-Butyl(2-(2-(2-(2-((2-(2,6-dioxopiperidin-3-yl)-1,3-dioxoisoindolin-4-yl)amino)ethoxy)ethoxy)ethoxy)ethyl)carbamate (**3a**)

Compound **3a** was prepared using General Procedure I and *tert*-butyl(2-(2-(2-(2-aminoethoxy)ethoxy)ethoxy)ethyl)carbamate (225 mg, 0.77 mmol). The crude product was purified by silica column chromatography (gradient of 30–80% EtOAc in Hex) to yield **3a** (170 mg, 40%) as a yellow oil. ^1^H NMR (500 MHz, acetone-*d*_6_): δ 9.98 (s, 1H), 7.58 (dd, *J* = 8.6, 7.1 Hz, 1H), 7.12 (d, *J* = 8.5 Hz, 1H), 7.04 (d, *J* = 7.1 Hz, 1H), 6.61 (t, *J* = 5.7 Hz, 1H), 5.92 (t, *J* = 6.0 Hz, 1H), 5.12–5.03 (m, 1H), 3.74 (t, *J* = 5.3 Hz, 2H), 3.66–3.59 (m, 4H), 3.60–3.51 (m, 6H), 3.47 (t, *J* = 5.8 Hz, 2H), 3.20 (q, *J* = 5.8 Hz, 2H), 3.02–2.90 (m, 2H), 2.83–2.71 (m, 2H), 2.21 (dtd, *J* = 10.6, 5.8, 2.9 Hz, 1H), 1.39 (d, *J* = 3.4 Hz, 9H). ^13^C NMR (126 MHz, acetone-*d*_6_): δ 172.8, 170.2, 170.1, 168.2, 156.5, 147.7, 136.8, 133.4, 117.8, 111.4, 110.9, 78.6, 71.2, 70.8, 70.5, 70.1, 49.8, 42.9, 40.9, 31.9, 28.6, 28.5, 23.3. A single peak in the aliphatic region could not be resolved, presumably due to signal overlap. HRMS (ESI): calcd for [C_28_H_40_N_4_O_9_Na]^+^, 599.2687; measured, 599.2690.

#### *tert*-Butyl(3-(4-(3-((2-(2,6-dioxopiperidin-3-yl)-1,3-dioxoisoindolin-4-yl)amino)propoxy)butoxy)propyl)carbamate (**4a**)

Compound **4a** was prepared using General Procedure I and *tert*-butyl(3-(4-(3-aminopropoxy)butoxy)propyl)carbamate (250 mg, 0.82 mmol). The crude product was purified by silica column chromatography (gradient of 20–70% EtOAc in Hex) to yield **4a** (182 mg, 40%) as a yellow oil. ^1^H NMR (400 MHz, acetone-*d*_6_): δ 9.95 (s, 1H), 7.58 (dd, *J* = 8.5, 7.1 Hz, 1H), 7.10 (d, *J* = 8.6 Hz, 1H), 7.02 (d, *J* = 7.0 Hz, 1H), 6.62 (t, *J* = 5.9 Hz, 1H), 5.94 (s, 1H), 5.06 (dd, *J* = 12.6, 5.4 Hz, 1H), 3.55 (t, *J* = 5.8 Hz, 2H), 3.43 (m, 8H), 3.13 (q, *J* = 6.6 Hz, 2H), 3.02–2.86 (m, 1H), 2.84–2.69 (m, 2H), 2.21 (ddt, *J* = 12.5, 5.5, 2.7 Hz, 1H), 1.92 (p, *J* = 6.3 Hz, 2H), 1.75–1.50 (m, 6H), 1.39 (s, 9H). ^13^C NMR (101 MHz, acetone-*d*_6_): δ 172.7, 170.2, 170.2, 168.3, 156.6, 147.8, 136.9, 133.6, 117.5, 111.2, 110.8, 78.3, 71.3, 71.2, 69.2, 69.1, 49.8, 41.0, 38.8, 32.0, 30.9, 30.1, 28.6, 27.3, 27.2, 23.4. HRMS (ESI): calcd for [C_28_H_40_N_4_O_8_Na]^+^, 583.2738; measured, 583.2731.

#### *tert*-Butyl(3-(2-(2-(3-((2-(2,6-dioxopiperidin-3-yl)-1,3-dioxoisoindolin-4-yl)amino)propoxy)ethoxy)ethoxy)propyl)carbamate (**5a**)

Compound **5a** was prepared using General Procedure I and *tert*-butyl(3-(2-(2-(3-aminopropoxy)ethoxy)ethoxy)propyl)carbamate (200 mg, 0.62 mmol). The crude product was purified by silica column chromatography (gradient of 50–80% EtOAc in Hex) to yield **5a** (110 mg, 31%) as a yellow oil. ^1^H NMR (400 MHz, acetone-*d*_6_): δ 9.93 (s, 1H), 7.58 (dd, *J* = 8.9, 7.3 Hz, 1H), 7.10 (d, *J* = 8.6 Hz, 1H), 7.02 (d, *J* = 7.1 Hz, 1H), 6.62 (t, *J* = 5.9 Hz, 1H), 5.94 (t, *J* = 5.8 Hz, 1H), 5.11–5.02 (m, 1H), 3.66–3.56 (m, 9H), 3.55–3.51 (m, 2H), 3.47 (q, *J* = 6.4 Hz, 4H), 3.14 (q, *J* = 6.5 Hz, 2H), 3.04–2.88 (m, 1H), 2.86–2.69 (m, 2H), 2.20 (ddt, *J* = 12.5, 5.5, 2.7 Hz, 1H), 1.93 (m, 2H), 1.75–1.65 (m, 2H), 1.39 (s, 9H). ^13^C NMR (101 MHz, acetone-*d*_6_): δ 172.7, 170.2, 170.1, 168.3, 156.6, 147.8, 136.9, 133.6, 117.5, 111.1, 110.7, 78.3, 71.1, 71.1, 71.1, 70.9, 69.5, 69.4, 49.8, 40.9, 38.7, 32.0, 30.8, 30.1, 28.6, 23.4. HRMS (ESI): calcd for [C_28_H_40_N_4_O_9_Na]^+^, 599.2687; measured, 599.2690.

#### *N*-(2-(2-((2-(2,6-Dioxopiperidin-3-yl)-1,3-dioxoisoindolin-4-yl)amino)ethoxy)ethyl)-4-sulfamoylbenzamide (**1**)

Compound **1** was prepared using General Procedure II and Boc-protected linker-pomalidomide candidate **1a** (182 mg, 0.325 mmol). The crude product was purified by silica column chromatography (gradient of 0–10% MeOH in DCM) to yield **1** (95 mg, 45%) as a green oil. ^1^H NMR (500 MHz, acetone-*d*_6_): δ 9.92 (s, 1H), 8.00 (d, *J* = 8.6 Hz, 2H), 7.93 (d, *J* = 8.4 Hz, 2H), 7.58–7.49 (m, 1H), 7.11 (d, *J* = 8.4 Hz, 1H), 7.00 (d, *J* = 7.0 Hz, 1H), 6.70 (s, 2H), 6.62 (t, *J* = 5.7 Hz, 1H), 5.06 (dd, *J* = 12.7, 5.5 Hz, 1H), 3.77 (t, *J* = 5.2 Hz, 2H), 3.71 (t, *J* = 5.5 Hz, 2H), 3.61 (q, *J* = 5.5 Hz, 2H), 3.54 (q, *J* = 5.4 Hz, 3H), 3.02–2.87 (m, 1H), 2.82–2.69 (m, 2H), 2.24–2.16 (m, 1H). ^13^C NMR (126 MHz, acetone-*d*_6_): δ 172.9, 170.4, 170.3, 168.3, 166.5, 147.8, 147.1, 138.8, 136.9, 133.5, 128.7, 126.9, 117.9, 111.6, 110.9, 70.0, 49.9, 43.0, 40.5, 32.0, 23.4. A single peak in the aliphatic region could not be resolved, presumably due to signal overlap. HRMS (ESI): calcd for [C_24_H_25_N_5_O_8_SNa]^+^, 566.1316; measured, 566.1313.

#### *N*-(2-(2-((2-(2,6-Dioxopiperidin-3-yl)-1,3-dioxoisoindolin-4-yl)amino)ethoxy)ethyl)-4-sulfamoylbenzamide (**2**)

Compound **2** was prepared using General Procedure II and Boc-protected linker-pomalidomide candidate **2a** (149 mg, 0.345 mmol). The crude product was purified by silica column chromatography (gradient of 0–10% MeOH in DCM) to yield **2** (22 mg, 22%) as a green oil. ^1^H NMR (500 MHz, acetone-*d*_6_): δ 9.93 (s, 1H), 8.00 (d, *J* = 8.2 Hz, 1H), 7.92 (d, *J* = 8.3 Hz, 2H), 7.88 (s, 1H), 7.57 (dd, *J* = 8.6, 7.0 Hz, 1H), 7.08 (d, *J* = 8.6 Hz, 1H), 7.03 (d, *J* = 7.1 Hz, 1H), 6.70 (s, 2H), 6.61 (t, *J* = 5.7 Hz, 1H), 5.07 (dd, *J* = 12.6, 5.5 Hz, 1H), 3.74 (t, *J* = 5.3 Hz, 2H), 3.65 (d, *J* = 5.8 Hz, 6H), 3.57 (q, *J* = 5.6 Hz, 2H), 3.50 (q, *J* = 5.4 Hz, 2H), 3.00–2.88 (m, 1H), 2.81–2.69 (m, 2H), 2.24–2.15 (m, 1H). ^13^C NMR (126 MHz, acetone-*d*_6_): δ 172.7, 170.4, 168.3, 166.3, 147.8, 147.2, 138.9, 137.0, 133.6, 128.6, 126.9, 117.8, 111.6, 111.1, 71.2, 71.0, 70.2, 70.0, 49.9, 43.0, 40.6, 32.0, 30.6, 23.4. HRMS (ESI): calcd for [C_26_H_29_N_5_O_9_SNa]^+^, 610.1578; measured, 610.1578.

#### *N*-(2-(2-(2-(2-((2-(2,6-Dioxopiperidin-3-yl)-1,3-dioxoisoindolin-4-yl)amino)ethoxy)ethoxy)ethoxy)ethyl)-4-sulfamoylbenzamide (**3**)

Compound **3** was prepared using General Procedure II and Boc-protected linker-pomalidomide candidate **3a** (170 mg, 0.310 mmol). The crude product was purified by silica column chromatography (gradient of 0–10% MeOH in DCM) to yield **3** (88 mg, 45%) as a green oil. ^1^H NMR (500 MHz, acetone-*d*_6_): δ 10.01 (s, 1H), 8.01 (d, *J* = 8.5 Hz, 2H), 7.94 (d, *J* = 8.5 Hz, 2H), 7.56 (dd, *J* = 8.6, 7.1 Hz, 1H), 7.08 (d, *J* = 8.5 Hz, 1H), 7.02 (d, *J* = 7.0 Hz, 1H), 6.76 (s, 2H), 6.58 (t, *J* = 5.7 Hz, 1H), 5.09 (dd, *J* = 12.5, 5.4 Hz, 1H), 3.70 (t, *J* = 5.3 Hz, 2H), 3.66–3.54 (m, 12H), 3.49 (q, *J* = 5.5 Hz, 2H), 3.02–2.91 (m, 1H), 2.85–2.70 (m, 2H), 2.26–2.17 (m, 1H). ^13^C NMR (126 MHz, acetone-*d*_6_): δ 172.9, 170.4, 170.2, 168.2, 166.4, 147.6, 147.1, 138.7, 136.9, 133.4, 128.6, 126.9, 117.8, 111.5, 110.8, 71.1, 71.1, 71.1, 70.8, 70.0, 70.0, 49.8, 42.8, 40.6, 31.9, 23.3. HRMS (ESI): calcd for [C_28_H_33_N_5_O_10_SNa]^+^, 654.1840; measured, 654.1838.

#### *N*-(3-(4-(3-((2-(2,6-Dioxopiperidin-3-yl)-1,3-dioxoisoindolin-4-yl)amino)propoxy)butoxy)propyl)-4-sulfamoylbenzamide (**4**)

Compound **4** was prepared using General Procedure II and Boc-protected linker-pomalidomide candidate **4a** (182 mg, 0.325 mmol). The crude product was purified by silica column chromatography (gradient of 0–10% MeOH in DCM) to yield **4** (95 mg, 45%) as a green oil. ^1^H NMR (500 MHz, acetone-*d*_6_): δ 9.98 (s, 1H), 8.01 (d, *J* = 8.5 Hz, 2H), 7.95 (d, *J* = 8.5 Hz, 2H), 7.58 (dd, *J* = 8.5, 7.0 Hz, 1H), 7.08 (d, *J* = 8.6 Hz, 1H), 7.02 (d, *J* = 7.1 Hz, 1H), 6.75 (s, 2H), 6.63 (t, *J* = 5.8 Hz, 1H), 5.08 (dd, *J* = 12.6, 5.4 Hz, 1H), 3.57–3.39 (m, 12H), 3.02–2.90 (m, 1H), 2.84–2.71 (m, 2H), 2.21 (ddt, *J* = 12.7, 5.5, 2.3 Hz, 1H), 1.89 (dp, *J* = 32.4, 6.4 Hz, 4H), 1.66–1.60 (m, 4H). ^13^C NMR (126 MHz, acetone-*d*_6_): δ 172.8, 170.3, 170.1, 168.3, 166.1, 147.7, 147.1, 139.0, 136.9, 133.6, 128.5, 126.9, 117.5, 111.2, 110.7, 71.4, 71.3, 69.3, 69.1, 49.8, 41.0, 38.4, 32.0, 30.3, 30.0, 27.3, 27.2, 23.4. HRMS (ESI): calcd for [C_30_H_37_N_5_O_10_SNa]^+^, 666.2204; measured, 666.2199.

#### *N*-(3-(2-(2-(3-((2-(2,6-Dioxopiperidin-3-yl)-1,3-dioxoisoindolin-4-yl)amino)propoxy)ethoxy)ethoxy)propyl)-4-sulfamoylbenzamide (**5**)

Compound **5** was prepared using General Procedure II and Boc-protected linker-pomalidomide candidate **5a** (110 mg, 0.191 mmol). The crude product was purified by silica column chromatography (gradient of 0–5% MeOH in DCM) to yield **5** (37 mg, 30%) as a green oil. ^1^H NMR (500 MHz, acetone-*d*_6_): δ 9.96 (s, 1H), 8.01 (d, *J* = 8.8 Hz, 2H), 7.95 (d, *J* = 8.5 Hz, 2H), 7.57 (dd, *J* = 8.6, 7.1 Hz, 1H), 7.09 (d, *J* = 8.6 Hz, 1H), 7.01 (d, *J* = 7.1 Hz, 1H), 6.74 (s, 2H), 6.62 (t, *J* = 5.8 Hz, 1H), 5.11–5.04 (m, 1H), 3.67–3.40 (m, 16H), 3.02–2.90 (m, 1H), 2.83–2.70 (m, 2H), 2.25–2.16 (m, 1H), 1.86 (dp, *J* = 25.3, 6.2 Hz, 4H). ^13^C NMR (126 MHz, acetone-*d*_6_): δ 172.8, 170.3, 170.1, 168.3, 166.0, 147.8, 147.1, 139.0, 136.9, 133.5, 128.6, 126.9, 117.6, 111.1, 110.7, 71.1, 71.0, 71.0, 70.9, 69.9, 69.5, 49.8, 40.9, 38.6, 32.0, 30.0, 29.8, 23.4. HRMS (ESI): calcd for [C_30_H_37_N_5_O_10_SNa]^+^, 682.2153; measured, 682.2151.

#### *tert*-Butyl(2-(2-(4-sulfamoylbenzamido)ethoxy)ethyl)carbamate (**6a**)

Compound **6a** was prepared using General Procedure III and *tert*-butyl(2-(2-aminoethoxy)ethyl)carbamate (150 mg, 0.734 mmol). The crude product was purified by silica column chromatography (gradient of 0–10% MeOH in DCM) to yield **6a** (240 mg, 84%) as a clear solid. ^1^H NMR (400 MHz, acetone-*d*_6_): δ 8.02 (m, 3H), 7.94 (d, *J* = 8.3 Hz, 2H), 6.78 (s, 2H), 6.03 (t, *J* = 5.7 Hz, 1H), 3.66–3.48 (m, 6H), 3.22 (q, *J* = 5.7 Hz, 2H), 1.37 (s, 9H). ^13^C NMR (101 MHz, acetone-*d*_6_): δ 166.6, 156.8, 147.2, 138.8, 128.7, 126.9, 78.7, 70.4, 69.9, 41.0, 40.5, 28.6. HRMS (ESI): calcd for [C_16_H_25_N_3_O_6_SNa]^+^, 410.1356; measured, 410.1359.

#### *tert*-Butyl(2-(2-(2-(4-sulfamoylbenzamido)ethoxy)ethoxy)ethyl)carbamate (**7a**)

Compound **7a** was prepared using General Procedure III and *tert*-butyl(2-(2-(2-aminoethoxy)ethoxy)ethyl)carbamate (125 mg, 0.503 mmol). The crude product was purified by silica column chromatography (gradient of 0–10% MeOH in DCM) to yield **7a** (109 mg, 50%) as a clear solid. ^1^H NMR (500 MHz, acetone-*d*_6_): δ 8.04 (d, *J* = 8.4 Hz, 2H), 7.96 (d, *J* = 8.5 Hz, 2H), 6.75 (s, 2H), 5.97 (s, 1H), 3.64 (t, *J* = 5.3 Hz, 2H), 3.58 (m, 6H), 3.48 (t, *J* = 5.8 Hz, 2H), 3.19 (q, *J* = 5.8 Hz, 2H), 1.39 (s, 9H). ^13^C NMR (126 MHz, acetone-*d*_6_): δ 166.3, 156.7, 147.2, 138.9, 128.6, 126.9, 78.7, 70.9, 70.8, 70.6, 70.1, 41.0, 40.6, 28.6. HRMS (ESI): calcd for [C_18_H_29_N_3_O_7_SNa]^+^, 454.1618; measured, 454.1619.

#### *tert*-Butyl(1-oxo-1-(4-sulfamoylphenyl)-5,8,11-trioxa-2-azatridecan-13-yl)carbamate (**8a**)

Compound **8a** was prepared using General Procedure III and *tert*-butyl(2-(2-(2-(2-aminoethoxy)ethoxy)ethoxy)ethyl)carbamate (150 mg, 0.513 mmol). The crude product was purified by silica column chromatography (gradient of 0–10% MeOH in DCM) to yield **8a** (168 mg, 69%) as a clear solid. ^1^H NMR (400 MHz, acetone-*d*_6_): δ 8.02 (d, *J* = 8.3 Hz, 3H), 7.95 (d, *J* = 8.4 Hz, 2H), 6.79 (s, 2H), 5.98 (t, *J* = 6.0 Hz, 1H), 3.71–3.51 (m, 12H), 3.45 (t, *J* = 5.8 Hz, 2H), 3.18 (q, *J* = 5.8 Hz, 2H), 1.38 (s, 9H). ^13^C NMR (101 MHz, acetone-*d*_6_): δ 166.4, 156.7, 147.2, 138.8, 128.7, 126.9, 78.7, 71.1, 71.0, 70.9, 70.8, 70.5, 70.0, 40.9, 40.6, 28.6. HRMS (ESI): calcd for [C_20_H_33_N_3_O_8_SNa]^+^, 498.1881; measured, 498.1880.

#### *tert*-Butyl(3-(4-(3-(4-sulfamoylbenzamido)propoxy)butoxy)propyl)carbamate (**9a**)

Compound **9a** was prepared using General Procedure III and *tert*-butyl(3-(4-(3-aminopropoxy)butoxy)propyl)carbamate (150 mg, 0.493 mmol). The crude product was purified by silica column chromatography (gradient of 0–10% MeOH in DCM) to yield **9a** (178 mg, 74%) as a clear solid. ^1^H NMR (400 MHz, acetone-*d*_6_): δ 8.00 (d, *J* = 8.5 Hz, 2H), 7.95 (d, *J* = 8.5 Hz, 2H), 6.76 (s, 2H), 6.04–5.86 (m, 1H), 3.55–3.35 (m, 11H), 3.13 (td, *J* = 6.9, 5.8 Hz, 2H), 1.86 (p, *J* = 6.5 Hz, 2H), 1.70 (p, *J* = 6.5 Hz, 2H), 1.60 (dq, *J* = 5.9, 2.8 Hz, 4H), 1.38 (s, 9H). ^13^C NMR (101 MHz, acetone-*d*_6_): δ 166.2, 156.6, 147.2, 139.1, 128.5, 126.9, 78.4, 71.3, 71.1, 69.4, 69.1, 38.8, 38.5, 30.9, 30.3, 28.6, 27.3, 27.3. HRMS (ESI): calcd for [C_22_H_37_N_3_O_7_SNa]^+^, 510.2244; measured, 510.2243.

#### *tert*-Butyl(1-oxo-1-(4-sulfamoylphenyl)-6,9,12-trioxa-2-azapentadecan-15-yl)carbamate (**10a**)

Compound **10a** was prepared using General Procedure III and *tert*-butyl(3-(2-(2-(3-aminopropoxy)ethoxy)ethoxy)propyl)carbamate (135 mg, 0.422 mmol). The crude product was purified by silica column chromatography (gradient of 0–10% MeOH in DCM) to yield **10a** (98 mg, 46%) as a clear solid. ^1^H NMR (500 MHz, acetone-*d*_6_): δ 8.06–8.04 (m, 1H), 8.02 (d, *J* = 8.5 Hz, 2H), 7.98–7.93 (m, 2H), 6.81 (s, 2H), 6.03 (d, *J* = 6.1 Hz, 1H), 3.62–3.55 (m, 8H), 3.53–3.47 (m, 4H), 3.44 (t, *J* = 6.2 Hz, 2H), 3.11 (q, *J* = 6.8 Hz, 2H), 1.85 (p, *J* = 6.4 Hz, 2H), 1.67 (p, *J* = 6.5 Hz, 2H), 1.38 (s, 9H). ^13^C NMR (126 MHz, acetone-*d*_6_): δ 166.1, 156.6, 147.1, 138.9, 128.5, 126.9, 78.4, 71.0, 71.0, 70.8, 70.8, 69.9, 69.4, 38.6, 38.5, 30.7, 30.1, 28.6. HRMS (ESI): calcd for [C_22_H_37_N_3_O_8_SNa]^+^, 526.2194; measured, 526.2191.

#### *N*-(2-(2-(2-((3*r*,5*r*,7*r*)-Adamantan-1-yl)acetamido)ethoxy)ethyl)-4-sulfamoylbenzamide (**6**)

Compound **6** was prepared using General Procedure IV and Boc-protected linker 4-SBA conjugate **6a** (240 mg, 0.619 mmol). The crude product was purified by silica column chromatography (gradient of 0–10% MeOH in DCM), followed by reverse phase C_18_ chromatography (gradient of 0–100% MeOH in H_2_O), to yield **6** (60 mg, 24%) as a clear oil. ^1^H NMR (400 MHz, methanol-*d*_4_): δ 7.95 (s, 4H), 3.66–3.49 (m, 6H), 3.36–3.32 (m, 2H), 1.88 (s, 5H), 1.69 (d, *J* = 12.0 Hz, 3H), 1.59 (dd, *J* = 14.3, 2.6 Hz, 9H). ^13^C NMR (101 MHz, methanol-*d*_4_): δ 174.0, 168.9, 147.8, 138.9, 129.1, 127.3, 70.6, 70.3, 51.8, 43.7, 41.1, 40.2, 37.9, 33.8, 30.1. HRMS (ESI): calcd for [C_23_H_34_N_3_O_5_S]^+^, 464.2214; measured, 464.2211.

#### *N*-(2-(2-(2-(2-((3*r*,5*r*,7*r*)-Adamantan-1-yl)acetamido)ethoxy)ethoxy)ethyl)-4-sulfamoylbenzamide (**7**)

Compound **7** was prepared using General Procedure IV and Boc-protected linker 4-SBA conjugate **7a** (109 mg, 0.253 mmol). The crude product was purified by silica column chromatography (gradient of 0–10% MeOH in DCM), followed by reverse phase C_18_ chromatography (gradient of 0–100% MeOH in H_2_O), to yield **7** (45 mg, 35%) as a clear oil. ^1^H NMR (500 MHz, acetone-*d*_6_): δ 8.13–8.07 (2, 1H), 8.07–8.01 (m, 2H), 7.99–7.93 (m, 2H), 7.12 (s, 1H), 6.83 (s, 2H), 3.64 (t, *J* = 5.4 Hz, 2H), 3.62–3.53 (m, 6H), 3.48 (t, *J* = 5.7 Hz, 2H), 3.28 (q, *J* = 5.7 Hz, 2H), 1.90 (s, 5H), 1.68 (m, 3H), 1.61 (m, 9H). ^13^C NMR (126 MHz, acetone-*d*_6_): δ 171.4, 166.5, 147.2, 138.7, 128.6, 126.9, 71.0, 70.9, 70.5, 70.0, 51.4, 43.2, 40.6, 39.6, 37.5, 33.2, 29.5. HRMS (ESI): calcd for [C_25_H_37_N_3_O_6_SNa]^+^, 530.2295; measured, 530.2292.

#### *N*-(1-((3*r*,5*r*,7*r*)-Adamantan-1-yl)-2-oxo-6,9,12-trioxa-3-azatetradecan-14-yl)-4-sulfamoylbenzamide (**8**)

Compound **8** was prepared using General Procedure IV and Boc-protected linker 4-SBA conjugate **8a** (168 mg, 0.447 mmol). The crude product was purified by silica column chromatography (gradient of 0–10% MeOH in DCM), followed by reverse phase C_18_ chromatography (gradient of 0–100% MeOH in H_2_O), to yield **8** (85 mg, 34%) as a clear solid. ^1^H NMR (400 MHz, chloroform-*d*_1_): δ 7.84 (s, 4H), 7.59 (m, 1H), 6.47 (s, 2H), 6.32 (m, 1H), 3.77–3.61 (m, 6H), 3.55–3.28 (m, 10H), 1.90 (m, 5H), 1.66 (m, 3H), 1.57 (m, 9H). ^13^C NMR (126 MHz, chloroform-*d*_1_): δ 171.8, 166.3, 145.2, 138.0, 128.1, 126.2, 70.6, 70.5, 70.4, 70.2, 70.1, 51.6, 42.7, 40.2, 39.2, 36.8, 32.8, 28.7. A single peak in the aliphatic region could not be resolved, presumably due to signal overlap. HRMS (ESI): calcd for [C_27_H_41_N_3_O_7_SNa]^+^, 574.2557; measured, 574.2552.

#### *N*-(3-(4-(3-(2-((3*r*,5*r*,7*r*)-Adamantan-1-yl)acetamido)propoxy)butoxy)propyl)-4-sulfamoylbenzamide (**9**)

Compound **9** was prepared using General Procedure IV and Boc-protected linker 4-SBA conjugate **9a** (178 mg, 0.355 mmol). The crude product was purified by silica column chromatography (gradient of 0–10% MeOH in DCM), followed by reverse phase C_18_ chromatography (gradient of 0–100% MeOH in H_2_O), to yield **9** (161 mg, 81%) as a clear solid. ^1^H NMR (400 MHz, chloroform-*d*_1_): δ 7.93 (d, *J* = 8.1 Hz, 2H), 7.83 (d, *J* = 8.1 Hz, 2H), 7.56 (t, *J* = 5.0 Hz, 1H), 6.28 (s, 1H), 5.95 (s, 2H), 3.64 (t, *J* = 5.3 Hz, 2H), 3.59 (q, *J* = 5.5 Hz, 2H), 3.48 (q, *J* = 5.9 Hz, 4H), 3.41 (t, *J* = 5.9 Hz, 2H), 3.32 (q, *J* = 6.4 Hz, 2H), 1.96–1.83 (m, 7H), 1.76 (p, *J* = 6.3 Hz, 2H), 1.70–1.47 (m, 16H). ^13^C NMR (101 MHz, chloroform-*d*_1_): δ 171.9, 165.7, 145.2, 138.3, 127.7, 126.7, 71.6, 71.6, 70.9, 69.6, 51.6, 42.7, 40.3, 38.1, 36.8, 32.9, 29.5, 28.7, 28.6, 27.2, 26.8. HRMS (ESI): calcd for [C_29_H_46_N_3_O_6_S]^+^, 564.3102; measured, 564.3101.

#### *N*-(1-((3*r*,5*r*,7*r*)-Adamantan-1-yl)-2-oxo-7,10,13-trioxa-3-azahexadecan-16-yl)-4-sulfamoylbenzamide (**10**)

Compound **10** was prepared using General Procedure IV and Boc-protected linker 4-SBA conjugate **10a** (150 mg, 0.290 mmol). The crude product was purified by silica column chromatography (gradient of 0–10% MeOH in DCM), followed by reverse phase C_18_ chromatography (gradient of 0–100% MeOH in H_2_O), to yield **10** (100 mg, 60%) as a clear solid. ^1^H NMR (500 MHz, acetone-*d*_6_): δ 8.13–8.09 (m, 1H), 8.05–8.01 (m, 2H), 7.96 (d, *J* = 8.4 Hz, 2H), 7.14 (d, *J* = 5.9 Hz, 1H), 6.89 (d, *J* = 2.0 Hz, 2H), 3.62–3.55 (m, 8H), 3.52–3.48 (m, 4H), 3.45 (t, *J* = 6.2 Hz, 2H), 3.21 (q, *J* = 6.5 Hz, 2H), 1.92–1.81 (m, 7H), 1.73–1.64 (m, 5H), 1.60 (d, *J* = 3.2 Hz, 9H). ^13^C NMR (126 MHz, acetone-*d*_6_): δ 171.2, 166.1, 147.2, 138.8, 128.6, 126.9, 71.0, 71.0, 70.8, 70.8, 69.9, 69.5, 51.5, 43.2, 38.6, 37.5, 37.2, 33.2, 30.5, 29.5. A single peak in the aliphatic region could not be resolved, presumably due to signal overlap. HRMS (ESI): calcd for [C_29_H_46_N_3_O_7_S]^+^, 580.3051; measured, 580.3046.

#### *tert*-Butyl(12-((2-(2,6-dioxopiperidin-3-yl)-1,3-dioxoisoindolin-4-yl)amino)dodecyl)carbamate (**11a**)

Compound **11a** was prepared using General Procedure I and *tert*-butyl(12-aminododecyl)carbamate (100 mg, 0.33 mmol). The crude product was purified by silica column chromatography (gradient of 20–50% EtOAc in Hex) to yield **11a** (170 mg, 40%) as a yellow oil. ^1^H NMR (400 MHz, acetonitrile-*d*_3_): δ 8.94 (s, 1H), 7.53 (dd, *J* = 8.5, 7.2 Hz, 1H), 7.01 (d, *J* = 7.8 Hz, 2H), 6.31 (t, *J* = 5.9 Hz, 1H), 5.24 (s, 1H), 4.93 (dd, *J* = 12.2, 5.4 Hz, 1H), 3.28 (q, *J* = 6.7 Hz, 2H), 2.98 (q, *J* = 6.7 Hz, 2H), 2.84–2.58 (m, 3H), 2.09 (m, 1H), 1.62 (p, *J* = 7.0 Hz, 2H), 1.32 (m, *J* = 47.8 Hz, 27H). ^13^C NMR (101 MHz, acetone-*d*_6_): δ 172.72, 170.33, 170.23, 168.27, 156.63, 147.81, 136.93, 133.61, 117.55, 111.22, 110.78, 78.24, 49.83, 43.12, 41.06, 32.00, 30.85, 30.33, 30.29, 29.96, 28.66, 27.61, 27.51, 23.41. Four peaks in the aliphatic region could not be resolved, presumably due to signal overlap. HRMS (ESI): calcd for [C_30_H_44_N_4_O_6_Na]^+^, 579.3153; measured, 579.3148.

#### *N*-(12-((2-(2,6-Dioxopiperidin-3-yl)-1,3-dioxoisoindolin-4-yl)amino)dodecyl)-4-sulfamoylbenzamide (**11**)

Compound **11** was prepared using General Procedure II, Boc-protected linker-pomalidomide candidate **11a** (46 mg, 81 μmol), and NHS-activated ester **12** (50 mg, 0.17 mmol). The crude product was purified by silica column chromatography (gradient of 0–10% MeOH in DCM) to yield **11** (33 mg, 64%) as a green oil. ^1^H NMR (500 MHz, DMSO-*d*_6_): δ 11.13 (s, 1H), 8.64 (t, *J* = 5.6 Hz, 1H), 8.01–7.94 (m, 2H), 7.91–7.84 (m, 2H), 7.57 (dd, *J* = 8.6, 7.1 Hz, 1H), 7.50 (s, 2H), 7.09 (d, *J* = 8.6 Hz, 1H), 7.01 (d, *J* = 7.0 Hz, 1H), 6.54 (t, *J* = 6.0 Hz, 1H), 5.05 (dd, *J* = 12.8, 5.4 Hz, 1H), 3.26 (dq, *J* = 15.6, 6.7 Hz, 4H), 2.88 (ddd, *J* = 16.9, 13.9, 5.4 Hz, 1H), 2.62–2.51 (m, 2H), 2.01 (ddp, *J* = 9.8, 4.4, 1.9 Hz, 1H), 1.53 (dp, *J* = 21.3, 7.0 Hz, 4H), 1.27 (d, *J* = 25.5 Hz, 16H). ^13^C NMR (101 MHz, DMSO-*d*_66_): δ 172.9, 170.2, 169.0, 167.4, 165.1, 146.5, 146.1, 137.6, 136.3, 132.2, 127.8, 125.6, 117.2, 110.4, 109.0, 48.5, 41.8, 31.0, 29.0, 28.8, 28.7, 26.5, 26.3, 25.3, 22.2. Five peaks in the aliphatic region could not be resolved, presumably due to signal overlap. HRMS (ESI): calcdHRMS (ESI): calcd for [C_32_H_42_N_5_O_7_S]^+^, 640.2799; measured, 640.2802.

#### 2,5-Dioxopyrrolidin-1-yl 4-Sulfamoylbenzoate (**12**)

N-Hydroxysuccinimide (0.86 g, 7.5 mmol) was added to a stirring solution of 4-sufamoyl benzoic acid (1.5 g, 7.5 mmol) in dry DMF (20 mL) at 0 °C. EDC–HCl (7.5 mmol) was then added to the reaction mixture at the same temperature. After stirring for 18 h at rt, the solution was dried. The resultant residue was re-dissolved in EtOAc (50 mL) and washed with H_2_O (3 × 30 mL), sodium bicarbonate solution (30 mL), and brine (3 × 10 mL). The organic layer was dried over MgSO_4_, filtered, and concentrated in vacuo. The final product was isolated to yield **12** (1.8 g, 81%) as a white powder. ^1^H NMR (400 MHz, DMSO-*d*_66_): δ 8.30 (d, *J* = 8.1 Hz, 2H), 8.07 (d, *J* = 8.1 Hz, 2H), 7.71 (s, 2H), 2.91 (s, 4H). ^13^C NMR (101 MHz, DMSO-*d*_66_): δ 170.3, 161.1, 149.9, 131.0, 127.2, 126.8, 25.6. HRMS (ESI): calcd for [C_11_H_9_N_2_O_6_S]^−^, 297.0187; measured, 297.0186.

#### *N*-(2-Methoxyethyl)-4-sulfamoylbenzamide (**13**)

2-Methoxyethan-1-amine (25 mg, 0.33 mmol) and NHS (0.11 g, 0.37 mmol) were dissolved in DMF (2 mL) at rt. After stirring for 22 h, the solution was dried in vacuo. The resulting residue was taken up in ethyl acetate (2 mL), poured into a 5% solution of sodium bicarbonate (2 mL), and extracted with ethyl acetate (3 × 2 mL). The combined organic layers were dried over sodium sulfate and filtered, and the solvent was removed in vacuo. The crude product was purified by silica column chromatography (gradient of 0–5% MeOH in DCM) to yield **13** (52 mg, 60%) as a white solid. ^1^H NMR (400 MHz, acetone-*d*_6_): δ 8.08–8.00 (m, 2H), 7.99–7.92 (m, 2H), 6.73 (d, *J* = 4.7 Hz, 1H), 3.61–3.48 (m, 4H), 3.30 (s, 3H). ^13^C NMR (101 MHz, acetone-*d*_6_): δ 206.3, 166.3, 147.3, 138.9, 128.6, 126.9, 71.6, 58.6, 40.4. HRMS (ESI): calcd for [C_10_H_13_N_2_O_4_S]^−^, 257.0602; measured, 257.0603.

#### 4-Fluoro-2-(1-methyl-2,6-dioxopiperidin-3-yl)isoindoline-1,3-dione (**14**)

To a stirring solution of 2-(2,6-dioxopiperidin-3-yl)-4-fluoroisoindoline-1,3-dione (150 mg, 543 μmol) in anhydrous DMF (1 mL) were added MeI (96 mg, 679 μmol) and K_2_CO_3_ (94 mg, 679 μmol). After 12 h of stirring at rt, the stirring suspension was diluted with water (5.0 mL) to precipitate the product. This mixture was then filtered and washed with water (3 × 2.5 mL) to yield **14** as a white solid (117 mg, 74%). ^1^H NMR (400 MHz, acetone-*d*_6_): δ 7.98–7.93 (m, 1H), 7.82–7.69 (m, 2H), 5.22 (dd, *J* = 13.1, 5.4 Hz, 1H), 3.02 (s, 3H), 3.00–2.87 (m, 1H), 2.80–2.74 (m, 1H), 2.59–2.48 (m, 1H), 2.11–2.04 (m, 1H). ^13^C NMR (101 MHz, acetone-*d*_6_): δ 171.8, 169.5, 166.2 (d, *J* = 28 Hz), 164.0, 158.2, 155.6, 138.2 (d, *J* = 78 Hz), 133.5, 123.2 (d, *J* = 195 Hz), 120.2 (d, *J* = 33 Hz), 117.1 (d, *J* = 125 Hz), 49.7, 31.1, 26.7, 21.1. HRMS (ESI): calcd for [C_14_H_12_FN_2_O_4_]^+^, 291.0776; measured, 291.0772.

#### 2,5-Dioxopyrrolidin-1-yl 4-(Methylsulfonyl)benzoate (**15**)

N-Hydroxysuccinimide (287 mg, 2.5 mmol) was added to a stirring solution of 4-(methylsulfonyl)benzoic acid (500 mg, 2.5 mmol) in dry DMF (6.5 mL) at 0 °C. EDC–HCl (2.5 mmol) was then added to the reaction mixture at the same temperature. After stirring for 18 h at rt, the solution was dried in vacuo. The resultant residue was re-dissolved in EtOAc (50 mL) and washed with H_2_O (3 × 30 mL), sodium bicarbonate solution (30 mL), and brine (3 × 10 mL). The organic layer was dried over MgSO_4_, filtered, and concentrated in vacuo. The final product was isolated to yield **15** (369 mg, 50%) as a white solid. ^1^H NMR (500 MHz, acetone-*d*_6_): δ 8.42–8.33 (m, 2H), 8.26–8.17 (m, 2H), 3.25 (s, 3H), 2.99 (s, 4H). ^13^C NMR (126 MHz, acetone-*d*_6_): δ 170.4, 161.9, 147.7, 131.9, 130.5, 129.1, 43.9, 26.4. Mass spectrometry data for this compound could not be obtained.

#### *N*-(3-(4-(3-((2-(2,6-Dioxopiperidin-3-yl)-1,3-dioxoisoindolin-4-yl)amino)propoxy)butoxy)propyl)-4-(methylsulfonyl)benzamide (**16**)

Compound **16** was prepared using General Procedure II, Boc-protected linker-pomalidomide candidate **4a** (57 mg, 99 μmol), and **15** (62 mg, 21 μmol). The crude product was purified by silica column chromatography (gradient of 0–10% MeOH in DCM) to yield **16** (48 mg, 75%) as a green oil. ^1^H NMR (400 MHz, acetone-*d*_6_): δ 9.94 (s, 1H), 8.11–8.06 (m, 2H), 8.04–8.00 (m, 2H), 7.98 (s, 1H), 7.58 (dd, *J* = 8.5, 7.1 Hz, 1H), 7.09 (d, *J* = 8.5 Hz, 1H), 7.02 (d, *J* = 7.1 Hz, 1H), 6.62 (t, *J* = 5.8 Hz, 1H), 5.10–5.03 (m, 1H), 3.58–3.38 (m, 12H), 3.16 (s, 3H), 3.04–2.89 (m, 1H), 2.84–2.69 (m, 2H), 2.21 (ddt, *J* = 12.5, 5.5, 2.7 Hz, 1H), 1.89 (dp, *J* = 24.2, 6.4 Hz, 4H), 1.64 (pd, *J* = 2.9, 1.3 Hz, 4H). ^13^C NMR (101 MHz, acetone-*d*_6_): δ 172.744, 170.276, 170.174, 168.291, 165.890, 147.808, 144.241, 140.584, 136.901, 133.639, 128.863, 128.242, 117.482, 111.174, 110.806, 71.428, 71.322, 69.395, 69.175, 49.815, 44.131, 41.056, 38.535, 31.994, 30.357, 27.375, 27.234, 23.413. One peak in the aliphatic region could not be resolved, presumably due to signal overlap. HRMS (ESI): calcd for [C_31_H_39_N_4_O_9_S]^+^, 643.2432; measured, 643.2433.

#### *tert*-Butyl(3-(4-(3-((2-(1-methyl-2,6-dioxopiperidin-3-yl)-1,3-dioxoisoindolin-4-yl)amino)propoxy)butoxy)propyl)carbamate (**17a**)

Compound **17a** was prepared using General Procedure I, *tert*-butyl(3-(4-(3-aminopropoxy)butoxy)propyl)carbamate (111 mg, 365 μmol), and **14** (106 mg, 365 μmol). The crude product was purified by silica column chromatography (gradient of 20–60% EtOAc in Hex) to yield **17a** (80 mg, 38%) as a yellow oil. ^1^H NMR (400 MHz, acetone-*d*_6_): δ 7.58 (dd, *J* = 8.6, 7.1 Hz, 1H), 7.09 (d, *J* = 8.6 Hz, 1H), 7.03 (d, *J* = 7.0 Hz, 1H), 6.61 (t, *J* = 5.8 Hz, 1H), 5.92 (s, 1H), 5.08 (dd, *J* = 13.0, 5.4 Hz, 1H), 3.55 (t, *J* = 5.8 Hz, 2H), 3.50–3.36 (m, 9H), 3.13 (q, *J* = 6.6 Hz, 2H), 3.09 (s, 3H), 3.07–2.82 (m, 2H), 2.74 (dtd, *J* = 13.9, 12.8, 4.6 Hz, 1H), 2.18 (dtd, *J* = 13.0, 5.3, 2.7 Hz, 1H), 1.99–1.87 (m, 2H), 1.70 (p, *J* = 6.5 Hz, 2H), 1.63 (tdd, *J* = 5.0, 2.6, 1.3 Hz, 4H), 1.39 (s, 9H). ^13^C NMR (101 MHz, acetone-*d*_6_): δ 172.213, 170.384, 170.178, 168.281, 156.557, 147.812, 136.892, 133.625, 117.464, 111.171, 110.799, 78.297, 71.415, 71.197, 69.170, 69.084, 50.377, 40.991, 38.805, 32.245, 30.927, 30.124, 28.622, 27.321, 27.210, 27.034, 22.659. HRMS (ESI): calcd for [C_29_H_43_N_4_O_8_]^+^, 575.3075; measured, 575.3078.

#### *N*-(3-(4-(3-((2-(1-Methyl-2,6-dioxopiperidin-3-yl)-1,3-dioxoisoindolin-4-yl)amino)propoxy)butoxy)propyl)-4-sulfamoylbenzamide (**17**)

Compound **17** was prepared using General Procedure II and Boc-protected linker-pomalidomide candidate **17a** (80 mg, 0.14 mmol). The crude product was purified by silica column chromatography (gradient of 0–5% MeOH in DCM) to yield **17** (38 mg, 42%) as a green oil. ^1^H NMR (400 MHz, acetone-*d*_6_): δ 8.03–7.98 (m, 2H), 7.97–7.90 (m, 3H), 7.57 (dd, *J* = 8.6, 7.1 Hz, 1H), 7.08 (d, *J* = 8.6 Hz, 1H), 7.04–6.98 (m, 1H), 6.74 (s, 2H), 6.61 (t, *J* = 5.8 Hz, 1H), 5.08 (dd, *J* = 13.0, 5.4 Hz, 1H), 4.05 (q, *J* = 7.1 Hz, 1H), 3.57–3.37 (m, 12H), 3.08 (s, 3H), 3.04–2.81 (m, 2H), 2.80–2.64 (m, 1H), 2.18 (dtd, *J* = 13.1, 5.3, 2.7 Hz, 1H), 1.88 (dp, *J* = 25.9, 6.3 Hz, 4H), 1.63 (dp, *J* = 5.0, 1.5 Hz, 4H). ^13^C NMR (101 MHz, acetone-*d*_6_): δ 172.3, 170.4, 170.2, 168.3, 166.1, 147.8, 147.1, 139.1, 136.9, 133.6, 128.5, 126.9, 117.5, 111.2, 110.7, 71.4, 71.3, 69.4, 69.1, 50.4, 41.0, 38.5, 32.2, 30.3, 27.3, 27.2, 27.0, 26.0, 22.6. HRMS (ESI): calcd for [C_31_H_39_N_5_O_9_SNa]^+^, 680.2361; measured, 680.2359.

#### *N*-(3-(2-(2-(3-((2-(2,6-Dioxopiperidin-3-yl)-1,3-dioxoisoindolin-4-yl)amino)propoxy)ethoxy)ethoxy)propyl)-4-(methylsulfonyl)benzamide (**18**)

Compound **18** was prepared using General Procedure II, Boc-protected linker-pomalidomide candidate **5a** (89 mg, 150 μmol), and **15** (94 mg, 320 μmol). The crude product was purified by silica column chromatography (gradient of 0–5% MeOH in DCM) to yield **18** (8 mg, 8%) as a green oil. ^1^H NMR (500 MHz, acetone-*d*_6_): δ 8.04–7.98 (m, 2H), 7.97–7.91 (m, 2H), 7.63–7.54 (m, 1H), 7.10 (d, *J* = 8.6 Hz, 1H), 7.01 (dd, *J* = 7.1, 0.6 Hz, 1H), 6.74 (d, *J* = 5.4 Hz, 2H), 6.62 (t, *J* = 5.8 Hz, 1H), 5.08 (dd, *J* = 13.0, 5.4 Hz, 1H), 3.63 (ddd, *J* = 5.3, 4.3, 3.1 Hz, 4H), 3.60–3.52 (m, 8H), 3.51–3.41 (m, 4H), 3.08 (s, 3H), 3.06–2.81 (m, 2H), 2.79–2.65 (m, 1H), 2.18 (dtd, *J* = 12.7, 5.3, 2.7 Hz, 1H), 1.95–1.80 (m, 4H). ^13^C NMR (101 MHz, acetone-*d*_6_): δ 171.4, 169.6, 169.3, 167.5, 165.1, 138.2, 136.1, 132.7, 127.7, 126.1, 126.1, 116.7, 116.7, 110.3, 109.9, 70.3, 70.2, 70.2, 70.0, 69.1, 68.6, 49.5, 40.0, 37.8, 31.4, 29.3, 29.2, 26.2, 21.8. HRMS (ESI): calcd for [C_31_H_38_N_4_O_10_SNa]^+^, 681.2201; measured, 681.2197.

#### *tert*-Butyl(3-(2-(2-(3-((2-(1-methyl-2,6-dioxopiperidin-3-yl)-1,3-dioxoisoindolin-4-yl)amino)propoxy)ethoxy)ethoxy)propyl)carbamate (**19a**)

Compound **19a** was prepared using General Procedure I, *tert*-butyl(3-(2-(2-(3-aminopropoxy)ethoxy)ethoxy)propyl)carbamate (147 mg, 458 μmol), and **14** (147 mg, 458 μmol). The crude product was purified by silica column chromatography (gradient of 20–80% EtOAc in Hex) to yield **19a** (93 mg, 34%) as a yellow oil. ^1^H NMR (400 MHz, acetone-*d*_6_): δ 7.59 (dd, *J* = 8.6, 7.1 Hz, 1H), 7.12 (d, *J* = 8.6 Hz, 1H), 7.02 (dd, *J* = 7.1, 0.6 Hz, 1H), 6.62 (t, *J* = 5.9 Hz, 1H), 5.94 (s, 1H), 5.08 (dd, *J* = 13.0, 5.4 Hz, 1H), 3.66–3.56 (m, 8H), 3.50 (dtd, *J* = 19.1, 5.9, 3.2 Hz, 6H), 3.13 (q, *J* = 6.6 Hz, 2H), 3.09 (s, 3H), 2.99 (ddd, *J* = 17.4, 13.8, 5.2 Hz, 1H), 2.88 (ddd, *J* = 17.4, 4.6, 2.8 Hz, 1H), 2.74 (dtd, *J* = 13.8, 12.8, 4.6 Hz, 1H), 2.23–2.12 (m, 1H), 1.92 (p, *J* = 6.3 Hz, 2H), 1.76–1.64 (m, 2H), 1.39 (d, *J* = 2.2 Hz, 9H). ^13^C NMR (101 MHz, acetone-*d*_6_): δ 172.2, 170.4, 170.2, 168.3, 156.6, 147.8, 136.9, 133.6, 117.6, 111.2, 110.7, 78.3, 71.2, 71.1, 71.1, 70.9, 69.6, 69.5, 50.4, 40.9, 38.7, 32.2, 30.8, 30.1, 28.6, 27.0, 22.6. HRMS (ESI): calcd for [C_29_H_43_N_4_O_9_]^+^, 591.3025; measured, 591.3021.

#### *N*-(3-(2-(2-(3-((2-(1-Methyl-2,6-dioxopiperidin-3-yl)-1,3-dioxoisoindolin-4-yl)amino)propoxy)ethoxy)ethoxy)propyl)-4-sulfamoylbenzamide (**19**)

Compound **19** was prepared using General Procedure II and Boc-protected linker-pomalidomide candidate **19a** (95 mg, 0.16 mmol). The crude product was purified by silica column chromatography (gradient of 0–10% MeOH in DCM) to yield **17** (13 mg, 12%) as a green oil. ^1^H NMR (500 MHz, acetone-*d*_6_): δ 8.03–8.00 (m, 2H), 7.96–7.94 (m, 2H), 7.58 (ddd, *J* = 8.6, 7.1, 0.6 Hz, 1H), 7.10 (d, *J* = 8.6 Hz, 1H), 7.01 (dd, *J* = 7.1, 0.6 Hz, 1H), 6.74 (d, *J* = 5.4 Hz, 1H), 6.62 (t, *J* = 5.8 Hz, 1H), 5.08 (dd, *J* = 13.0, 5.4 Hz, 1H), 3.63 (ddd, *J* = 5.3, 4.3, 3.1 Hz, 4H), 3.59–3.52 (m, 8H), 3.51–3.43 (m, 4H), 3.08 (s, 3H), 3.06–2.81 (m, 3H), 2.73 (dtd, *J* = 13.9, 12.9, 4.6 Hz, 1H), 2.18 (dtd, *J* = 12.7, 5.3, 2.7 Hz, 1H), 1.93–1.81 (m, 4H). ^13^C NMR (126 MHz, acetone-*d*_6_): δ 172.3, 170.4, 170.2, 168.3, 165.9, 139.1, 137.0, 133.6, 128.6, 126.9, 126.9, 117.6, 117.5, 111.2, 110.7, 71.1, 71.1, 71.1, 70.9, 70.0, 69.5, 50.4, 40.9, 38.6, 32.2, 30.2, 30.1, 27.0, 22.6. HRMS (ESI): calcd for [C_31_H_39_N_5_O_10_SNa]^+^, 696.2310; measured, 696.2313.

### Chemical Reagents for Biological Assays, Tissue Culture, and Western Blot Analysis

*p*-Nitrophenyl acetate (N8130), HEPES (H3375), β-mercaptoethanol (BME, M6250), phenylmethylsulfonyl fluoride (P7626), lenalidomide (SML2283), acetazolamide (A6011), and MG-132 readymade solution (M7449) were purchased from Sigma-Aldrich. iBright prestained protein ladder (LC5615) was purchase from Thermo Fisher Scientific. Primary antibodies (hCAII Rabbit mAb, #124687 and β-actin Rabbit pAb, #8227) and secondary antibodies (Goat Anit-Rabbit IgC Cy5 preadsorbed, #97077) were purchased from Abcam.

### hCAII Assay

Recombinant hCAII was expressed and purified according to the protocol reported in the Supporting Information. Assays were carried out in clear-bottom Corning 96-well polystyrene plates (Costar, 3370). Wells were prepared to a final volume of 100 μL per well, including buffer (50 mM HEPES, pH 8.0), enzyme (hCAII, 40 nM), inhibitor (varying concentrations), and substrate (*p*-nitrophenyl acetate, 500 μM). Inhibitors and protein were preincubated for 10 min at rt, and the substrate was added to the reaction mixture immediately before reading. The change in absorbance at 405 nM was monitored for 20 min at 1 min intervals using a BioTek Synergy H4 plate reader, and the rates of the reaction over the first 15 min were determined. The rate of absorbance for each replicate was corrected for substrate autohydrolysis and inherent inhibitor absorbance by subtracting the rate of absorbance from a blank sample lacking enzyme but containing the inhibitor and substrate. Samples lacking inhibitor but containing enzyme and substrate were used to define 100% hCAII activity. Samples lacking inhibitor and enzyme were used to define 0% hCAII activity. IC_50_ was determined using non-linear regression with a variable slope on the GraphPad Prism software.

### Cell Lines and Culture Methods

HEK293 cells were cultured in Dulbecco’s modified Eagle’s medium (DMEM, Gibco, 4.5 g/L glucose) supplemented with 10% fetal bovine serum (FBS), 1% sodium pyruvate, and 1% penicillin/streptomycin. Cells were grown at 37 °C in a humidified 5% CO_2_ atmosphere and regularly tested for mycoplasma.

### Western Blot Analysis

After reaching 90% confluency, HEK293 cells were trypsinized, counted, and plated with 2 mL media at 2 × 10^6^ cells per well on a tissue-culture-treated, 6-well plate. At the appropriate number of hours before reaching ∼90% confluency, cells were treated with DMSO solutions of the appropriate compounds (final concentration 0.5% DMSO per well). Upon reaching 90% confluency, typically 72 h, culture media were removed and the cells were washed with cold Dulbecco’s phosphate-buffered saline (DPBS). Lysis buffer, composed of cell lysis buffer (Cell Signaling Technology, 9803), protease inhibitor cocktail (Cell Signaling Technology, 5872), and 1 mM phenylmethylsulfonylfluoride, was added to each well, and the plates were rocked at 4 °C for 20 min. Cells were then scraped from the bottom of the plates, and the supernatant was collected after spinning down at 14,000*g* at 4 °C for 10 min and frozen at −80 °C. Lysates were thawed on ice, and the total protein concentration was measured using a Pierce bicinchoninic acid protein assay (Thermo Fisher Scientific, 23225). SDS-PAGE samples were prepared by combining 30–50 μg of the total protein and sample buffer composed of a non-reducing, fluorescent compatible sample buffer (Thermo Fisher Scientific, LC2570) with a 2.5% final concentration of BME. Following heating at 98 °C for 5 min and cooling to room temperature, the prepared samples were electrophoretically separated on a 4–20% TGX gel (Bio-Rad, 4568095) and transferred to a PVDF membrane (Amersham Hybond P 0.45 μm, 106000019) in a tris-glycine transfer buffer (Thermo Fisher Scientific, LC3675) with a 20% final MeOH concentration. The membrane was rocked at 4 °C with fluorescent blocking buffer (Thermo Fisher Scientific, 37565) for 1 h and then incubated with primary antibodies (hCAII Rabbit mAb, 1:1000 and β-actin Rabbit pAb, 1:2000 in fluorescent blocking buffer) at 4 °C overnight. The membrane was washed three times with tris-buffered saline-Tween 20 (TBS-T, 0.1% w/v Tween 20) before rocking with secondary antibodies (Goat Anit-Rabbit IgC Cy5 preadsorbed, 1:2000 in fluorescent blocking buffer) for 1 h at rt. Following another round of three TBS-T washes, the membrane was briefly rinsed with deionized water and imaged on an Amersham Typhoon Biomolecular Imager. The immunoblot was then analyzed using the ImageJ software as an 8-bit image. The brightness and contrast of the image were adjusted, and the integrated density was calculated for each protein band using the gel lane analysis tool. Relative hCAII expression was calculated by normalizing the hCAII/β-actin ratio in compound-treated wells to the hCAII/β-actin ratio in vehicle (DMSO)-treated wells and indicated as “hCAII Abundance (% DMSO)”.

## Supporting Information Available

Inhibition assay details, complete western blots, protein expression, purification, and crystallization details, crystallography statistics tables, NMR spectra of compounds, and HPLC traces of compounds (PDF)
SMILES file for compounds (CSV)

## Abbreviations

AAZ: acetazolamide
BME: β-mercaptoethanol
Boc: *tert*-butyloxycarbonyl
CRBN: cereblon E3 ubiquitin ligase
DCM: dichloromethane
DMF: dimethyl formamide
DMSO: dimethyl sulfoxide
DMEM: Dulbecco’s modified Eagle’s medium
DPBS: Dulbecco’s phosphate-buffered saline
EtOAc: ethyl acetate
EDC: 1-ethyl-3-(3-dimethylaminopropyl)carbodiimide hydrochloride
FBS: fetal bovine serum
FDA: Food and Drug Administration
DC_50_: half maximal degradation
IC_50_: half maximal inhibitory concentration
HATU: hexafluorophosphate azabenzotriazole tetramethyl uronium
Hex: hexanes
hCA: human carbonic anhydrase
HDAC: human deacetylase
HEK293: human embryonic kidney 293
*D*_Max_: maximum percentage of degradation
MoA: mechanism of action
MeOH: methanol
MBP: metal-binding pharmacophore
DIPEA: *N*,*N*-diisoproylethylamine
NHS: *N*-hydroxysuccinimide
PEG: polyethylene glycol
PVDF: polyvinylidene difluoride
POI: protein of interest
PROTAC: proteolysis targeting chimera
S_N_Ar: nucleophilic aromatic substitution
rt: room temperature
SAR: structure–activity relationship
TPDs: targeted protein degraders
TFA: trifluoroacetic acid
TBS-T: tris-buffered saline-Tween 20
UPS: ubiquitin-proteasome system

## References

[ref1] Esbaugh A. J.; Tufts B. L. The structure and function of carbonic anhydrase isozymes in the respiratory system of vertebrates. Respir. Physiol. Neurobiol. 2006, 154, 185–198. 10.1016/j.resp.2006.03.007. 16679072

[ref2] Occhipinti R.; Boron W. F. Role of Carbonic Anhydrases and Inhibitors in Acid-Base Physiology: Insights from Mathematical Modeling. Int. J. Mol. Sci. 2019, 20, 3841–3871. 10.3390/ijms20153841. 31390837 PMC6695913

[ref3] Al-Samir S.; Papadopoulos S.; Scheibe R. J.; Meißner J. D.; Cartron J. P.; Sly W. S.; Alper S. L.; Gros G.; Endeward V. Activity and distribution of intracellular carbonic anhydrase II and their effects on the transport activity of anion exchanger AE1/SLC4A1. J. Physiol. 2013, 591, 4963–4982. 10.1113/jphysiol.2013.251181. 23878365 PMC3810803

[ref4] Lehenkari P.; Hentunen T. A.; Laitala-Leinonen T.; Tuukkanen J.; Väänänen H. K. Carbonic anhydrase II plays a major role in osteoclast differentiation and bone resorption by effecting the steady state intracellular pH and Ca2+. Exp. Cell Res. 1998, 242, 128–137. 10.1006/excr.1998.4071. 9665810

[ref5] Nocentini A.; Donald W. A.; Supuran C. T. Chapter 8—Human Carbonic Anhydrases: Tissue Distribution, Physiological Role, and Druggability. In Carbonic Anhydrases; Supuran C. T., Nocentini A., Eds.; Academic Press, 2019; pp 151–185.

[ref6] Imtaiyaz Hassan M.; Shajee B.; Waheed A.; Ahmad F.; Sly W. S. Structure, function and applications of carbonic anhydrase isozymes. Bioorg. Med. Chem. 2013, 21, 1570–1582. 10.1016/j.bmc.2012.04.044. 22607884

[ref7] Krishnamurthy V. M.; Kaufman G. K.; Urbach A. R.; Gitlin I.; Gudiksen K. L.; Weibel D. B.; Whitesides G. M. Carbonic Anhydrase as a Model for Biophysical and Physical-Organic Studies of Proteins and Protein–Ligand Binding. Chem. Rev. 2008, 108, 946–1051. 10.1021/cr050262p. 18335973 PMC2740730

[ref8] Aggarwal M.; Boone C. D.; Kondeti B.; McKenna R. Structural annotation of human carbonic anhydrases. J. Enzyme Inhib. Med. Chem. 2013, 28, 267–277. 10.3109/14756366.2012.737323. 23137351

[ref9] Mishra C. B.; Tiwari M.; Supuran C. T. Progress in the development of human carbonic anhydrase inhibitors and their pharmacological applications: Where are we today?. Med. Res. Rev. 2020, 40, 2485–2565. 10.1002/med.21713. 32691504

[ref10] Zamanova S.; Shabana A. M.; Mondal U. K.; Ilies M. A. Carbonic anhydrases as disease markers. Expert Opin. Ther. Pat. 2019, 29, 509–533. 10.1080/13543776.2019.1629419. 31172829 PMC6612586

[ref11] Meldrum N. U.; Roughton F. J. Carbonic anhydrase. Its preparation and properties. J. Physiol. 1933, 80, 113–142. 10.1113/jphysiol.1933.sp003077. 16994489 PMC1394121

[ref12] Krebs H. A. Inhibition of carbonic anhydrase by sulphonamides. Biochem. J. 1948, 43, 525–528. 10.1042/bj0430525. 16748445 PMC1274769

[ref13] Supuran C. T. Carbonic anhydrases: novel therapeutic applications for inhibitors and activators. Nat. Rev. Drug Discovery 2008, 7, 168–181. 10.1038/nrd2467. 18167490

[ref14] Quigley H. A. Glaucoma. Lancet 2011, 377, 1367–1377. 10.1016/s0140-6736(10)61423-7. 21453963

[ref15] Bonardi A.; Nocentini A.; Bua S.; Combs J.; Lomelino C.; Andring J.; Lucarini L.; Sgambellone S.; Masini E.; McKenna R.; Gratteri P.; Supuran C. T. Sulfonamide Inhibitors of Human Carbonic Anhydrases Designed through a Three-Tails Approach: Improving Ligand/Isoform Matching and Selectivity of Action. J. Med. Chem. 2020, 63, 7422–7444. 10.1021/acs.jmedchem.0c00733. 32519851 PMC8008423

[ref16] La Regina G.; Puxeddu M.; Nalli M.; Vullo D.; Gratteri P.; Supuran C. T.; Nocentini A.; Silvestri R. Discovery of New 1,1′-Biphenyl-4-sulfonamides as Selective Subnanomolar Human Carbonic Anhydrase II Inhibitors. ACS Med. Chem. Lett. 2020, 11, 633–637. 10.1021/acsmedchemlett.9b00437. 32435363 PMC7236029

[ref17] Krasavin M.; Korsakov M.; Dorogov M.; Tuccinardi T.; Dedeoglu N.; Supuran C. T. Probing the ‘bipolar’ nature of the carbonic anhydrase active site: Aromatic sulfonamides containing 1,3-oxazol-5-yl moiety as picomolar inhibitors of cytosolic CA I and CA II isoforms. Eur. J. Med. Chem. 2015, 101, 334–347. 10.1016/j.ejmech.2015.06.022. 26160114

[ref18] Noor S. I.; Jamali S.; Ames S.; Langer S.; Deitmer J. W.; Becker H. M. A surface proton antenna in carbonic anhydrase II supports lactate transport in cancer cells. eLife 2018, 7, e35176 10.7554/eLife.35176. 29809145 PMC5986270

[ref19] Becker H. M.; Hirnet D.; Fecher-Trost C.; Sültemeyer D.; Deitmer J. W. Transport Activity of MCT1 Expressed in Xenopus Oocytes Is Increased by Interaction with Carbonic Anhydrase. J. Biol. Chem. 2005, 280, 39882–39889. 10.1074/jbc.m503081200. 16174776

[ref20] Becker M.; Klier M.; Schüler C.; McKenna R.; Deitmer W. Intramolecular proton shuttle supports not only catalytic but also noncatalytic function of carbonic anhydrase II. Proc. Natl. Acad. Sci. U.S.A. 2011, 108, 3071–3076. 10.1073/pnas.1014293108. 21282642 PMC3041061

[ref21] Lai A. C.; Crews C. M. Induced protein degradation: an emerging drug discovery paradigm. Nat. Rev. Drug Discovery 2017, 16, 101–114. 10.1038/nrd.2016.211. 27885283 PMC5684876

[ref22] Békés M.; Langley D. R.; Crews C. M. PROTAC targeted protein degraders: the past is prologue. Nat. Rev. Drug Discovery 2022, 21, 181–200. 10.1038/s41573-021-00371-6. 35042991 PMC8765495

[ref23] Bassi Z. I.; Fillmore M. C.; Miah A. H.; Chapman T. D.; Maller C.; Roberts E. J.; Davis L. C.; Lewis D. E.; Galwey N. W.; Waddington K. E.; Parravicini V.; Macmillan-Jones A. L.; Gongora C.; Humphreys P. G.; Churcher I.; Prinjha R. K.; Tough D. F. Modulating PCAF/GCN5 Immune Cell Function through a PROTAC Approach. ACS Chem. Biol. 2018, 13, 2862–2867. 10.1021/acschembio.8b00705. 30200762

[ref24] Sun X.; Wang J.; Yao X.; Zheng W.; Mao Y.; Lan T.; Wang L.; Sun Y.; Zhang X.; Zhao Q.; Zhao J.; Xiao R.-P.; Zhang X.; Ji G.; Rao Y. A chemical approach for global protein knockdown from mice to non-human primates. Cell Discovery 2019, 5, 10 10.1038/s41421-018-0079-1. 30729032 PMC6361926

[ref25] Bondeson D. P.; Mares A.; Smith I. E. D.; Ko E.; Campos S.; Miah A. H.; Mulholland K. E.; Routly N.; Buckley D. L.; Gustafson J. L.; Zinn N.; Grandi P.; Shimamura S.; Bergamini G.; Faelth-Savitski M.; Bantscheff M.; Cox C.; Gordon D. A.; Willard R. R.; Flanagan J. J.; Casillas L. N.; Votta B. J.; den Besten W.; Famm K.; Kruidenier L.; Carter P. S.; Harling J. D.; Churcher I.; Crews C. M. Catalytic in vivo protein knockdown by small-molecule PROTACs. Nat. Chem. Biol. 2015, 11, 611–617. 10.1038/nchembio.1858. 26075522 PMC4629852

[ref26] Gao H.; Sun X.; Rao Y. PROTAC Technology: Opportunities and Challenges. ACS Med. Chem. Lett. 2020, 11, 237–240. 10.1021/acsmedchemlett.9b00597. 32184950 PMC7073876

[ref27] Hamilton E. P.; Schott A. F.; Nanda R.; Lu H.; Keung C. F.; Gedrich R.; Parameswaran J.; Han H. S.; Hurvitz S. A. ARV-471, an estrogen receptor (ER) PROTACdegrader, combined with palbociclib in advanced ER+/human epidermal growth factor receptor 2-negative (HER2-) breast cancer: Phase 1b cohort (part C) of a phase 1/2 study. J. Clin. Oncol. 2022, 40, TPS1120 10.1200/jco.2022.40.16_suppl.tps1120.

[ref28] Gao X.; Burris III H. A. Iii; Vuky J.; Dreicer R.; Sartor A. O.; Sternberg C. N.; Percent I. J.; Hussain M. H. A.; Rezazadeh Kalebasty A.; Shen J.; Heath E. I.; Abesada-Terk G.; Gandhi S. G.; McKean M.; Lu H.; Berghorn E.; Gedrich R.; Chirnomas S. D.; Vogelzang N. J.; Petrylak D. P. Phase 1/2 study of ARV-110, an androgen receptor (AR) PROTAC degrader, in metastatic castration-resistant prostate cancer (mCRPC). J. Clin. Oncol. 2022, 40, 17 10.1200/jco.2022.40.6_suppl.017.

[ref29] Credille C. V.; Morrison C. N.; Stokes R. W.; Dick B. L.; Feng Y.; Sun J.; Chen Y.; Cohen S. M. SAR Exploration of Tight-Binding Inhibitors of Influenza Virus PA Endonuclease. J. Med. Chem. 2019, 62, 9438–9449. 10.1021/acs.jmedchem.9b00747. 31536340

[ref30] Bondeson D. P.; Smith B. E.; Burslem G. M.; Buhimschi A. D.; Hines J.; Jaime-Figueroa S.; Wang J.; Hamman B. D.; Ishchenko A.; Crews C. M. Lessons in PROTAC Design from Selective Degradation with a Promiscuous Warhead. Cell Chem. Biol. 2018, 25, 78–87. 10.1016/j.chembiol.2017.09.010. 29129718 PMC5777153

[ref31] Yang K.; Song Y.; Xie H.; Wu H.; Wu Y. T.; Leisten E. D.; Tang W. Development of the first small molecule histone deacetylase 6 (HDAC6) degraders. Bioorg. Med. Chem. Lett. 2018, 28, 2493–2497. 10.1016/j.bmcl.2018.05.057. 29871848

[ref32] Alabi S.; Jaime-Figueroa S.; Yao Z.; Gao Y.; Hines J.; Samarasinghe K. T. G.; Vogt L.; Rosen N.; Crews C. M. Mutant-selective degradation by BRAF-targeting PROTACs. Nat. Commun. 2021, 12, 920 10.1038/s41467-021-21159-7. 33568647 PMC7876048

[ref33] Wu H.; Yang K.; Zhang Z.; Leisten E. D.; Li Z.; Xie H.; Liu J.; Smith K. A.; Novakova Z.; Barinka C.; Tang W. Development of Multifunctional Histone Deacetylase 6 Degraders with Potent Antimyeloma Activity. J. Med. Chem. 2019, 62, 7042–7057. 10.1021/acs.jmedchem.9b00516. 31271281

[ref34] Xiao Y.; Wang J.; Zhao L. Y.; Chen X.; Zheng G.; Zhang X.; Liao D. Discovery of histone deacetylase 3 (HDAC3)-specific PROTACs. Chem. Commun. 2020, 56, 9866–9869. 10.1039/d0cc03243c. PMC7654701 32840532

[ref35] Smalley J. P.; Adams G. E.; Millard C. J.; Song Y.; Norris J. K. S.; Schwabe J. W. R.; Cowley S. M.; Hodgkinson J. T. PROTAC-mediated degradation of class I histone deacetylase enzymes in corepressor complexes. Chem. Commun. 2020, 56, 4476–4479. 10.1039/d0cc01485k. PMC7610821 32201871

[ref36] Smalley J. P.; Baker I. M.; Pytel W. A.; Lin L.-Y.; Bowman K. J.; Schwabe J. W. R.; Cowley S. M.; Hodgkinson J. T. Optimization of Class I Histone Deacetylase PROTACs Reveals that HDAC1/2 Degradation is Critical to Induce Apoptosis and Cell Arrest in Cancer Cells. J. Med. Chem. 2022, 65, 5642–5659. 10.1021/acs.jmedchem.1c02179. 35293758 PMC9014412

[ref37] Wu T.; Yoon H.; Xiong Y.; Dixon-Clarke S. E.; Nowak R. P.; Fischer E. S. Targeted protein degradation as a powerful research tool in basic biology and drug target discovery. Nat. Struct. Mol. Biol. 2020, 27, 605–614. 10.1038/s41594-020-0438-0. 32541897 PMC7923177

[ref38] Oh E.; Akopian D.; Rape M. Principles of Ubiquitin-Dependent Signaling. Annu. Rev. Cell Dev. Biol. 2018, 34, 137–162. 10.1146/annurev-cellbio-100617-062802. 30110556

[ref39] Pettersson M.; Crews C. M. PROteolysis TArgeting Chimeras (PROTACs) - Past, present and future. Drug Discovery Today: Technol. 2019, 31, 15–27. 10.1016/j.ddtec.2019.01.002. PMC6578591 31200855

[ref40] Ottis P.; Crews C. M. Proteolysis-Targeting Chimeras: Induced Protein Degradation as a Therapeutic Strategy. ACS Chem. Biol. 2017, 12, 892–898. 10.1021/acschembio.6b01068. 28263557

[ref41] Raina K.; Crews C. M. Targeted protein knockdown using small molecule degraders. Curr. Opin. Chem. Biol. 2017, 39, 46–53. 10.1016/j.cbpa.2017.05.016. 28605671 PMC5584562

[ref42] Cyrus K.; Wehenkel M.; Choi E. Y.; Han H. J.; Lee H.; Swanson H.; Kim K. B. Impact of linker length on the activity of PROTACs. Mol. BioSyst. 2011, 7, 359–364. 10.1039/c0mb00074d. 20922213 PMC3835402

[ref43] Wang H.; Zhuang J.; Raghupathi K. R.; Thayumanavan S. A supramolecular dissociation strategy for protein sensing. Chem. Commun. 2015, 51, 17265–17268. 10.1039/c5cc07408h. PMC4659772 26462172

[ref44] Steinebach C.; Sosič I.; Lindner S.; Bricelj A.; Kohl F.; Ng Y. L. D.; Monschke M.; Wagner K. G.; Krönke J.; Gütschow M. A MedChem toolbox for cereblon-directed PROTACs. MedChemComm 2019, 10, 1037–1041. 10.1039/c9md00185a. 31304001 PMC6596386

[ref45] Boriack P. A.; Christianson D. W.; Kingery-Wood J.; Whitesides G. M. Secondary interactions significantly removed from the sulfonamide binding pocket of carbonic anhydrase II influence inhibitor binding constants. J. Med. Chem. 1995, 38, 2286–2291. 10.1021/jm00013a004. 7608893

[ref46] Seo H.; Jackl M. K.; Kalaj M.; Cohen S. M. Developing Metal-Binding Isosteres of 8-Hydroxyquinoline as Metalloenzyme Inhibitor Scaffolds. Inorg. Chem. 2022, 61, 7631–7641. 10.1021/acs.inorgchem.2c00891. 35507007 PMC9912809

[ref47] Nocentini A.; Supuran C. T. Advances in the structural annotation of human carbonic anhydrases and impact on future drug discovery. Expert Opin. Drug Discovery 2019, 14, 1175–1197. 10.1080/17460441.2019.1651289. 31436118

[ref48] Karlsson M.; Zhang C.; Méar L.; Zhong W.; Digre A.; Katona B.; Sjöstedt E.; Butler L.; Odeberg J.; Dusart P.; Edfors F.; Oksvold P.; von Feilitzen K.; Zwahlen M.; Arif M.; Altay O.; Li X.; Ozcan M.; Mardinoglu A.; Fagerberg L.; Mulder J.; Luo Y.; Ponten F.; Uhlén M.; Lindskog C. A single-cell type transcriptomics map of human tissues. Sci. Adv. 2021, 7, eabh2169 10.1126/sciadv.abh2169. 34321199 PMC8318366

[ref49] Douglass E. F.; Miller C. J.; Sparer G.; Shapiro H.; Spiegel D. A. A Comprehensive Mathematical Model for Three-Body Binding Equilibria. J. Am. Chem. Soc. 2013, 135, 6092–6099. 10.1021/ja311795d. 23544844 PMC3717292

[ref50] Rock K. L.; Gramm C.; Rothstein L.; Clark K.; Stein R.; Dick L.; Hwang D.; Goldberg A. L. Inhibitors of the proteasome block the degradation of most cell proteins and the generation of peptides presented on MHC class I molecules. Cell 1994, 78, 761–771. 10.1016/s0092-8674(94)90462-6. 8087844

[ref51] Drummond M. L.; Henry A.; Li H.; Williams C. I. Improved Accuracy for Modeling PROTAC-Mediated Ternary Complex Formation and Targeted Protein Degradation via New In Silico Methodologies. J. Chem. Inf. Model. 2020, 60, 5234–5254. 10.1021/acs.jcim.0c00897. 32969649

[ref52] Fischer E. S.; Böhm K.; Lydeard J. R.; Yang H.; Stadler M. B.; Cavadini S.; Nagel J.; Serluca F.; Acker V.; Lingaraju G. M.; Tichkule R. B.; Schebesta M.; Forrester W. C.; Schirle M.; Hassiepen U.; Ottl J.; Hild M.; Beckwith R. E. J.; Harper J. W.; Jenkins J. L.; Thomä N. H. Structure of the DDB1-CRBN E3 ubiquitin ligase in complex with thalidomide. Nature 2014, 512, 49–53. 10.1038/nature13527. 25043012 PMC4423819

[ref53] Sakamoto K. M.; Kim K. B.; Kumagai A.; Mercurio F.; Crews C. M.; Deshaies R. J. Protacs: Chimeric molecules that target proteins to the Skp1-Cullin-F box complex for ubiquitination and degradation. Proc. Natl. Acad. Sci. U.S.A. 2001, 98, 8554–8559. 10.1073/pnas.141230798. 11438690 PMC37474

