# Supplementary material for: Development of Human Carbonic Anhydrase II Heterobifunctional Degraders
Source: J Med Chem. 2023 Feb 3;66(4):2789–803. doi: 10.1021/acs.jmedchem.2c01843 (PMC9969396; doi:10.1021/acs.jmedchem.2c01843)
Supplement: Supplementary file 1 — jm2c01843_si_001.pdf [file jm2c01843_si_001.pdf]

Supporting Information

Development of Human Carbonic Anhydrase II Heterobifunctional Degraders

Conor B. O'Herin, Yuta W. Moriuchi, Troy A. Bemis, Alysia J. Kohlbrand, Michael D. Burkart, and Seth M. Cohen**

Department of Chemistry and Biochemistry, University of California, La Jolla, California 92093, United States

Corresponding Authors: Michael D. Burkart (mburkart@ucsd.edu) and Seth M. Cohen (scohen@ucsd.edu)

Table of Contents

hCAII Inhibition Activity of Acetazolamide, Compounds **1-10**, and **13** S3

Full Western Blot Images for Figures 2, 3, and 4 S4

Full Western Blot Images for Figure 5 S5

Full Western Blot Images of Additional Biological Replicates for Figures 3 and 5 S6

Protein Expression and Purification S7

Protein Crystallography S10

Cocrystal Structures of **1**, **2**, **4**, and **13** with hCAII S11

Protein Crystallography Refinement Statistics S12

Ternary Complex Modeling S13

NMR Spectra for Compounds **1-11** and **13** S14

HPLC Trace for Compounds **1-11** and **13** S26

Table S1. hCAII Inhibition Activity of Acetazolamide, Compounds **1-10**, and **13**.

**Compound**	IC_{50} (nM)
**AAZ**	≤ 20 nM
**1**	48 ± 5
**2**	37 ± 5
**3**	41 ± 5
**4**	≤ 20 nM
**5**	≤ 20 nM
**6**	57 ± 5
**7**	≤ 20 nM
**8**	28 ± 4
**9**	35 ± 5
**10**	≤ 20 nM
**13**	58 ± 8

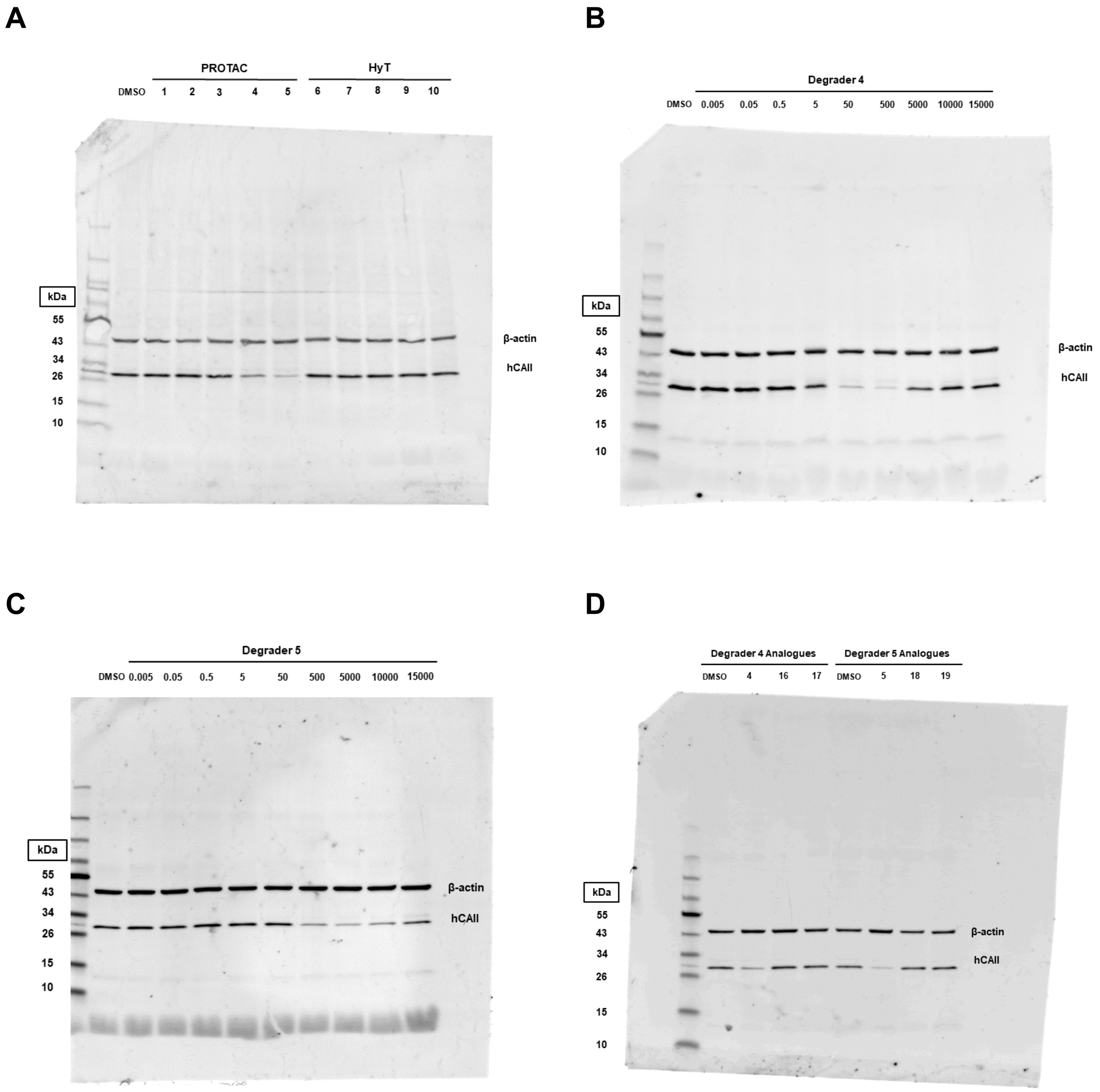

Figure S1. Images of full western blots shown in the main text Figures 2, 3, and 4. Full blots: (A) Figure 2, (B) Figure 3A (*left*), (C) Figure 3A (*right*), (D) Figure 4B.

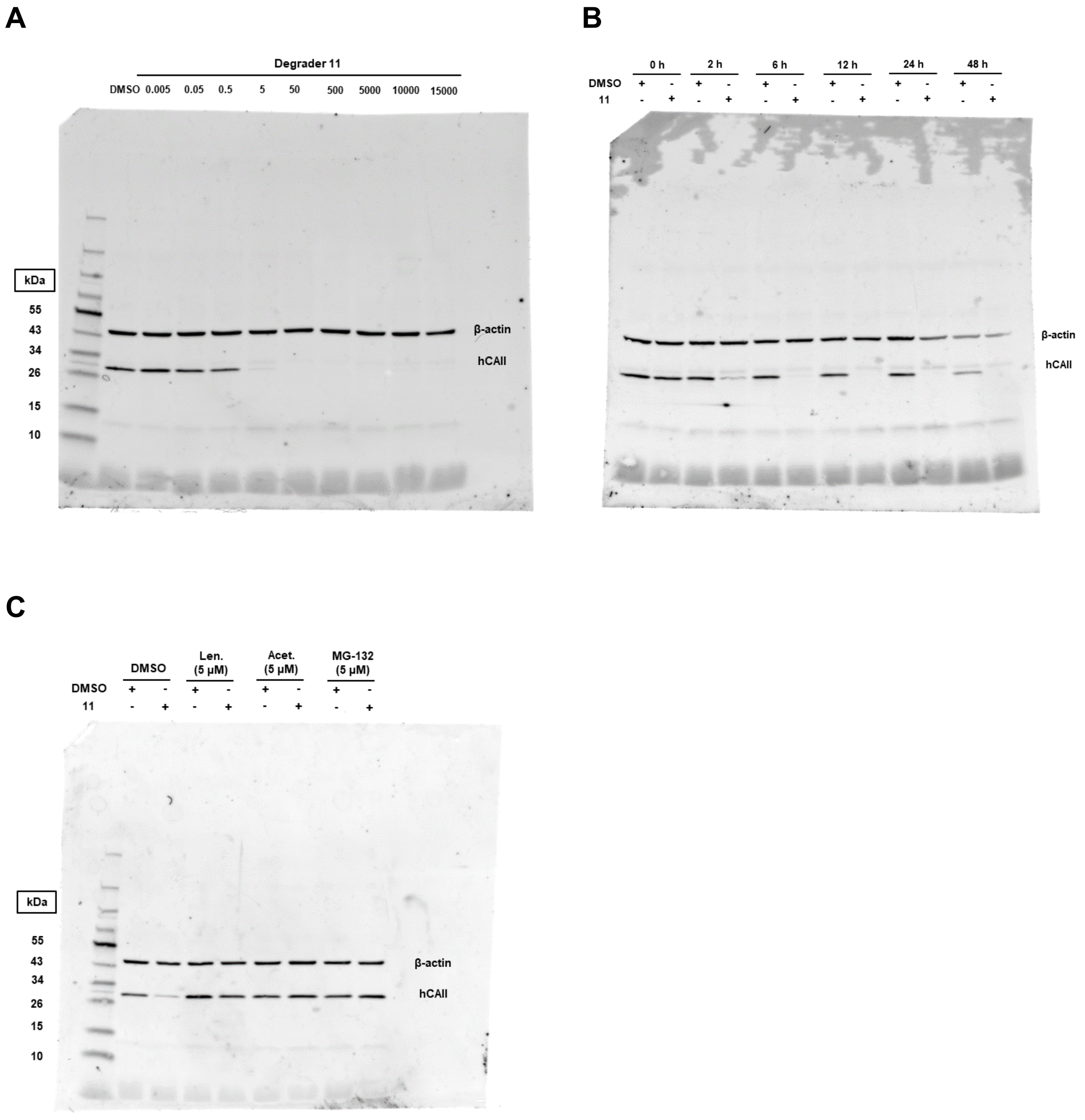

Figure S2. Images of full western blots shown in the main text Figure 5. Full blots: (A) Figure 5A, (B) Figure 5C, (C) Figure 5D.

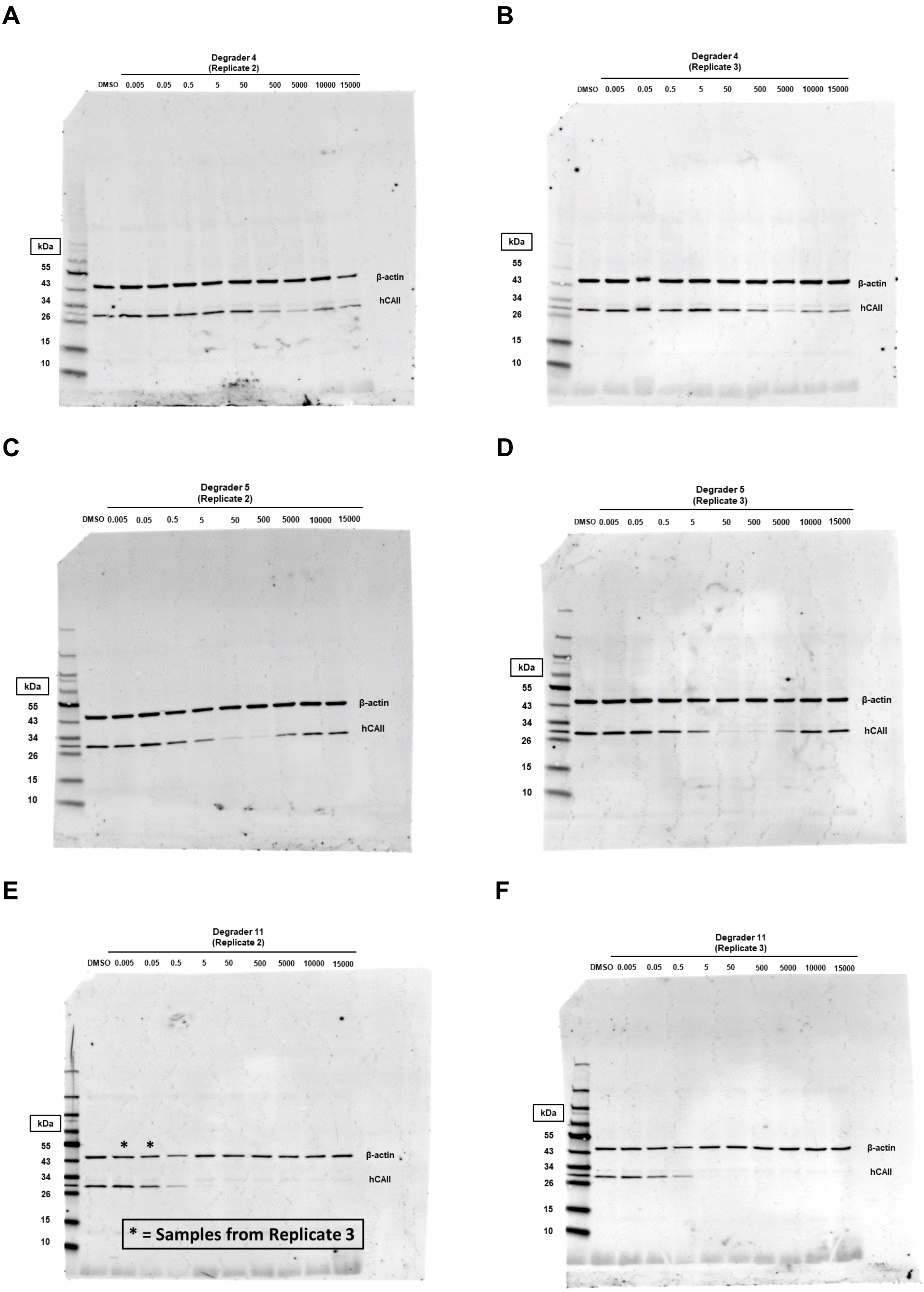

Figure S3. Images of full western blots of second and third biological replicates quantified for dose response curves in Figure 3 and Figure 5. Full blots: (A, B) Figure 3B (*left*), (C, D) Figure 3B (*right*), (E, F) Figure 5B.

Chemical Reagents for Protein Expression & Purification

Mini, EDTA-free protease inhibitor cocktail tablets (Roche, 11836170001), chloramphenicol (Sigma, C0378), ampicillin (Simga, A9393), IPTG (Sigma, I5502), phenylmethylsulfonyl fluoride (Sigma, P7626), *Nα-p*-tosyl-L-arginine methyl ester hydrochloride (Sigma, T4626), *p*-chloromercuribenzoic acid (Sigma, C49607) was purchased from Millipore Sigma.

Protein Expression & Purification

Plasmids encoding human carbonic anhydrase isozyme II and containing a T7 RNA polymerase promoter and an ampicillin resistance gene (pACA) were a generous gift from Carol A. Fierke, University of Michigan Medical School. Plasmids were introduced into BL21 (DE3) *Escherichia coli* cells via heat-shock (45 s at 42 °C) and incubated in sterile SOC medium for 1 h at 37 °C with gentle shaking. The cells were then placed onto agar plates containing 60 μg/mL ampicillin and 34 μg/mL chloramphenicol and incubated overnight at 37 °C. Single colonies were transferred to 30 mL of autoclaved LB medium (10 g/L tryptone, 5 g/L yeast extract, 10 g/L NaCl) containing 100 μg /mL ampicillin and 34 μg /mL chloramphenicol and incubated overnight at 37 °C. Six pre-cultures were used to inoculate 6 L of autoclaved induction media (20 g/L tryptone, 10 g/L yeast extract, 5 g/L NaCl, 0.36 × M9 salts solution, 0.4 % glucose, 60 μM $ZnSO_4$, 100 μg/mL ampicillin and 34 μg/mL chloramphenicol). Cells were shaken (250 rpm) in induction media at 37 °C until OD600 = 0.6 – 0.8. Addition of isopropyl-β-D-thiogalactopyranoside (IPTG, 250 μM final concentration) and $ZnSO_4$ (450 μM final concentration) induced protein expression and the temperature was lowered to 30 °C. The protease inhibitors phenylmethanesulfonyl fluoride (PMSF, 8 μg/mL) and *Nα-p*-tosyl-L-arginine methyl ester hydrochloride (TAME, 1 μg/mL) were added to the induction media after 3 h. The cells were shaken for an additional 3 h after addition of protease inhibitors (for a total of 6 h induction) and pelleted via centrifugation (4,400 rpm at 4 °C) for 15 min. Carbonic

anhydrase expression was confirmed via SDS-PAGE and Coomassie staining. Cell paste was flash frozen and stored at -80 °C prior to lysis.

Cell paste was thawed in batches on ice for 2 h and resuspended in an equal volume of lysis buffer (1% Triton-X, 200 μM $ZnSO_4$, 2 mM DTT, 10-100 μg/mL DNAse-1, 1 mg/mL lysozyme, and 1% glycerol with one mini, EDTA-free protease inhibitor tablet (Roche) per 50 mL added prior to resuspension) to cell pellet. Cells were lysed using a probe sonicator (Fisherbrand model 120) with cycles of 25 second pulses and 59 second rest at 60% amplitude. Cell debris was then pelleted by centrifugation (10,000 rpm at 4°C) for 45 min. The supernatant was decanted from the pellet and dialyzed against 4 L of activity buffer (50 mM Tris-sulfate, pH 8.0, and 0.5 mM $ZnSO_4$).

Cell lysates were slowly mixed with DEAE-Sephacel ion exchange resin (GE-Healthcare, 17-0500-01) equilibrated with activity buffer (50 mM Tris-sulfate, pH 8.0, and 0.5 mM $ZnSO_4$) and 1 mM DTT for 1h at 4 °C. The mixture was filtered using a Nalgene filter flask (0.45 μm) and the resin was washed three times with activity buffer. The eluent was combined and dialyzed in activity buffer overnight at 4 °C. The protein is then purified by affinity chromatography with 25 mL of 4-aminomethylbenzene sulfonamide agarose resin packed in a XK 16/20 column. Briefly, the column was equilibrated with 5 CV of activity buffer (50 mM Tris-sulfate, pH 8.0, and 0.5 mM $ZnSO_4$) or until absorbance reached a steady baseline. Dialyzed protein was loaded to the column from a 150 mL Superloop at a flow rate of 0.5 mL/min. The column was then washed with 5 CV of wash buffer (50 mM Na_2PO_4H, 50 mM KSCN, and 25 mM Tris at pH 8.8). The bound protein was then eluted with 10 CV of elution buffer (200 mM KSCN and 50 mM Na_2PO_4H at pH 5.6) at a flow rate of 2 mL/min collecting 2 mL fractions. hCAII eluted between fractions 13-41. SDS-PAGE analysis showed a band corresponding to hCAII running at ~29 kDa with only one small impurity at ~50 kDa. Pure fractions were pooled and dialyzed against 4 L of activity buffer for 24 h at 4 °C. A portion

of this protein was then either concentrated to 80 nM for activity assays or 20 mg/ml for crystallography and flash frozen. The remaining protein was dialyzed against 4L of DI water for 24 h at 4 °C and mQ water for 24 h at 4 °C and then lyophilized. All protein samples are stored at -80 °C.

Protein Crystallography

Crystals of hCAII were obtained by the hanging-drop vapor diffusion method using 24-well pre-greased plates (Hampton, HR3-171) with siliconized glass slides (Hampton, HR3-231). The protein solution consisted of 20 mg/mL hCAII and 1 mM *p*-chloromercuribenzoic acid in 50 mM Tris-SO_4 (pH 8.0) supplemented with 0.5 mM $ZnSO_4$. Protein solution was incubated on ice with 1 mM compound for 1 h prior to setting the crystallization drops. The precipitant solution contained 2.6-3.0 M $(NH_4)_2SO_4$ in 50 mM Tris-SO_4 (pH 8). Drops consisting of 3 μL of protein solution and 2.5-4.0 μL of precipitant solution were equilibrated at room temperature against 500 μL of precipitant solution. Colorless crystals roughly 0.3 × 0.3 × 0.3 mm in size appeared after 7-10 days. Crystals formation occurred spontaneously, but streak crystal seeding with natural or artificial cat whiskers produced larger and more abundant single crystals.

Collected crystals were cryoprotected with perfluoroether (Hampton, HR2-814) prior to flash freezing and were stored in liquid nitrogen until data collection. All structures include a 4-mercuribenzoic acid ligand bound to Cys206. X-ray diffraction studies were carried out on a Bruker Microfocus Rotating Anode (MicroStar FR-592) X-ray generator with a Bruker APEX II CCD detector at wavelength 1.54178 Å. Data was integrated, scaled, and merged using the Bruker APEX3 software package (Bruker, 2017). All crystals belong to the monoclinic space group $P2_1$. The data were phased by molecular replacement using a previously reported hCAII structure (PDB: 4E49), with water molecules removed, using PHASER. All structures were refined with Phenix version 1.19.2 and model building and visualized using Coot version 0.9.5.

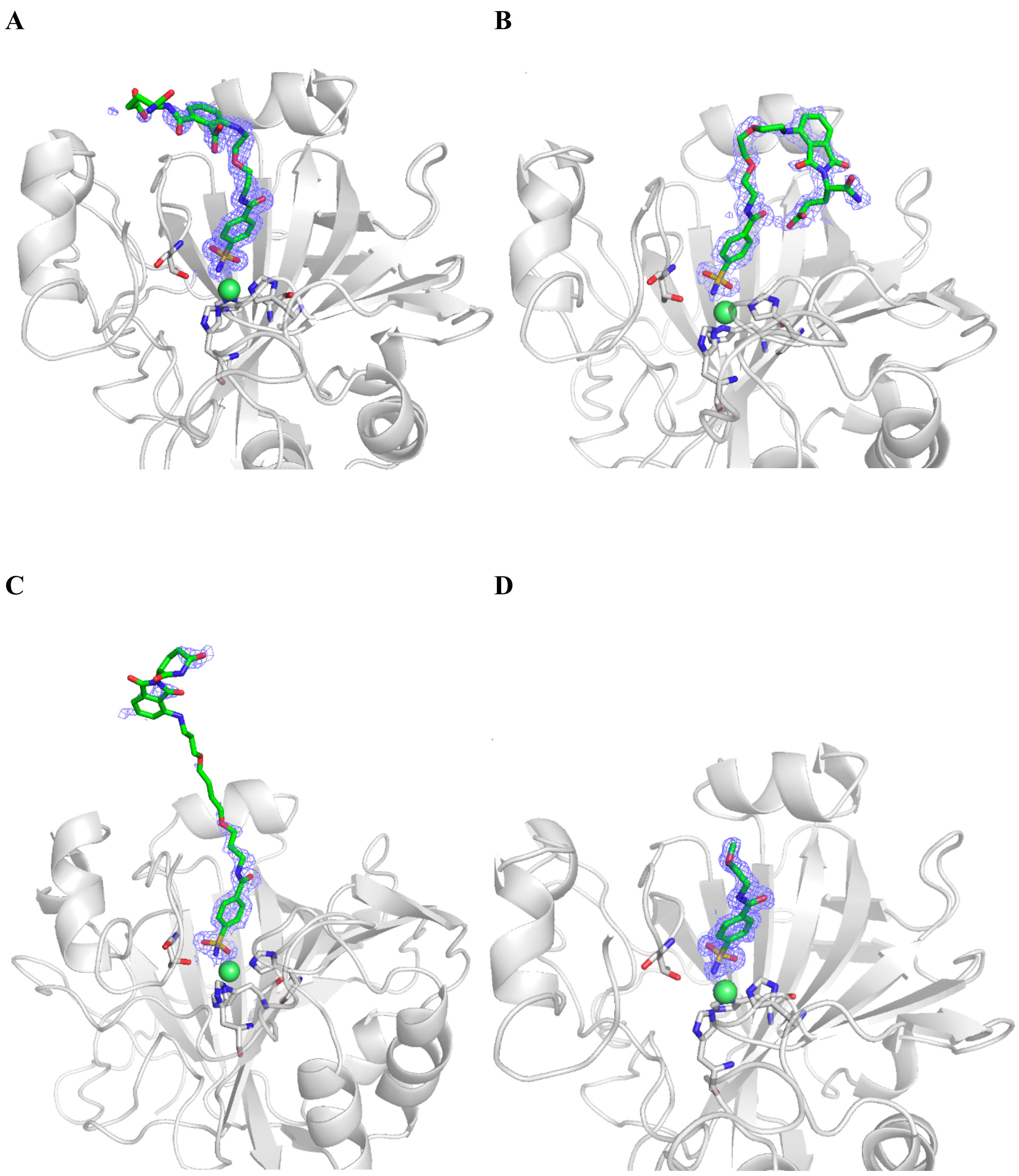

Figure S4. Density Maps of Compounds **1**, **2**, **4**, and **13** cocrystalized with hCAII. Compound **1** (A), **2** (B), **4** (C), and **13** (D).

Table S2. X-ray crystallographic data collection and refinement statistics.

Compound	**1**	**2**	**4**	**13**
**PDB**	8EYL	8EXG	8EXC	8EMU
**Data collection statistics**				
Resolution range	40.64-1.18 (1.21-1.18)	40.91- 1.13 (1.17-1.13)	34.84- 1.90 (1.95-1.93)	40.97-1.13 (1.17-1.13)
Space group	P_12	P_12	P_12	P_12
Cell dimensions a, b, c, (Å) α, β, γ (°)	41.872 41.036 71.269 90 103.954 90	42.207 41.659 71.948 90 104.268 90	41.986 41.222 71.874 90 104.211 90	42.174, 41.37, 71.834, 90, 103.896, 90
Unique reflections	71372	85925	18888	495273
Completeness (%)	99	99	99	96
Mean I/sigma(I)	15 (2.2)	12 (2.0)	11 (2.7)	12 (2.1)
R-merge	0.080 (0.223)	0.091 (0.605)	0.072 (0.256)	0.091 (0.587)
R-measured	0.091 (0.605)	0.100 (0.736)	0.082 (0.320)	0.1 (0.713)
R-work	0.1815 (0.2780)	0.2233 (0.2634)	0.1768 (0.2222)	0.2147 (0.3786)
R-free	0.1932 (0.2993)	0.1872 (0.2715)	0.2267 (0.2718)	0.2325 (0.3601)
RMS(bonds)	0.008	0.006	0.008	0.008
RMS(angles)	1.09	0.87	1.18	1.13
Ramachandran favored (%)	95.69	96.86	95.69	96.86
Ramachandran outliers (%)	0	0	0	0
Average B-factor	24.3	21	24.3	13.7
Redundancy	4.1	5.8	3.9	5.8
CC1/2	0.999 (0.975)	0.999 (0.748)	0.997 (0.928)	0.999 (0.752)

* Metrics for highest resolution shell given in parentheses

Ternary Complex Modeling

Ternary complex modeling was performed in MOE using Method 4B, as described in Drummond, et al,[1] which was accessed as "PROTAC-Modeling Tools in MOE" through the SVL exchange on the Chemical Computing Group website. Receptor-ligand complexes of hCAII bound to compound **13** (PDB: 8EMU) and CRBN bound to pomalidomide (PDB: 4CI3) were prepared in MOE using a "QuickPrep" method. Structures of PROTAC molecules were generated in MOE using the builder function, minimized, and saved as a molecular database file (*.mdb*). Multiple protein-protein docking runs were performed to match hydrophobic patches and protein patch statistics were calculated for docked poses. The 'Conformations:' setting was set to 'LowModeMD' to generate conformations for the PROTAC molecular database, and the remaining options in the 'Conformational Search & Filters Setting' were left at their default values. Final results were clustered according to the reported double-clustering method and a rigid-body minimization was performed to alleviate clashing between PRTOAC and the proteins on all final ternary complexes. The ternary complex model of **11** in complex with hCAII and CRBN in Figure 6 was the pose with the lowest total forcefield interaction energy selected from the most populous double cluster.

NMR Spectra for Compounds 1-11 and 13.

^{1}H NMR of Compound **1**:

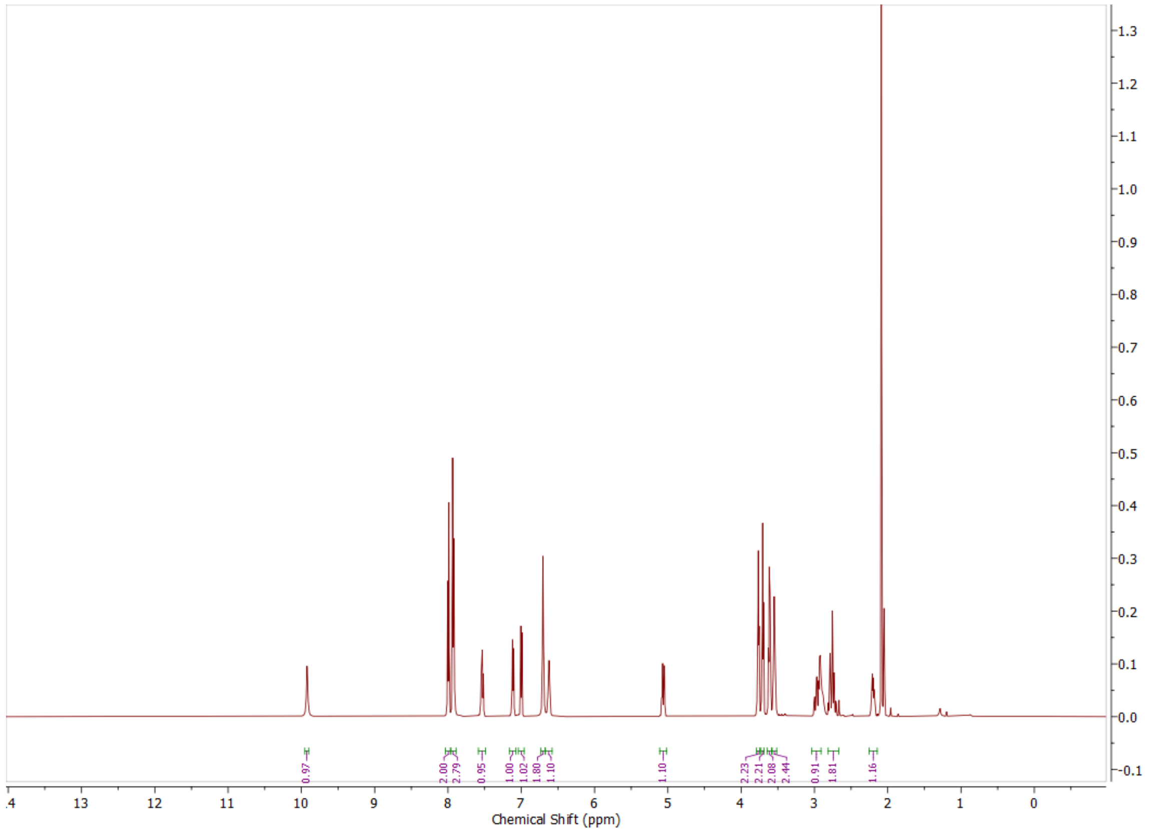

^{13}C NMR of Compound **1**:

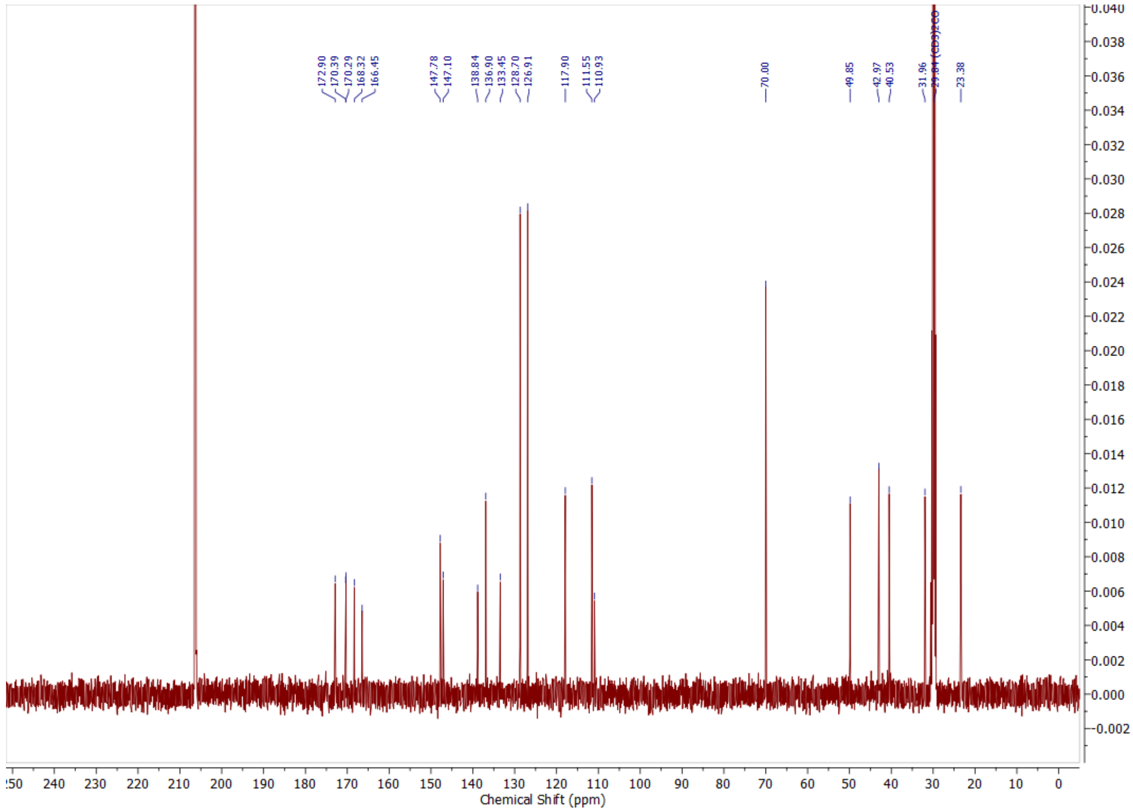

^{1}H NMR of Compound **2**:

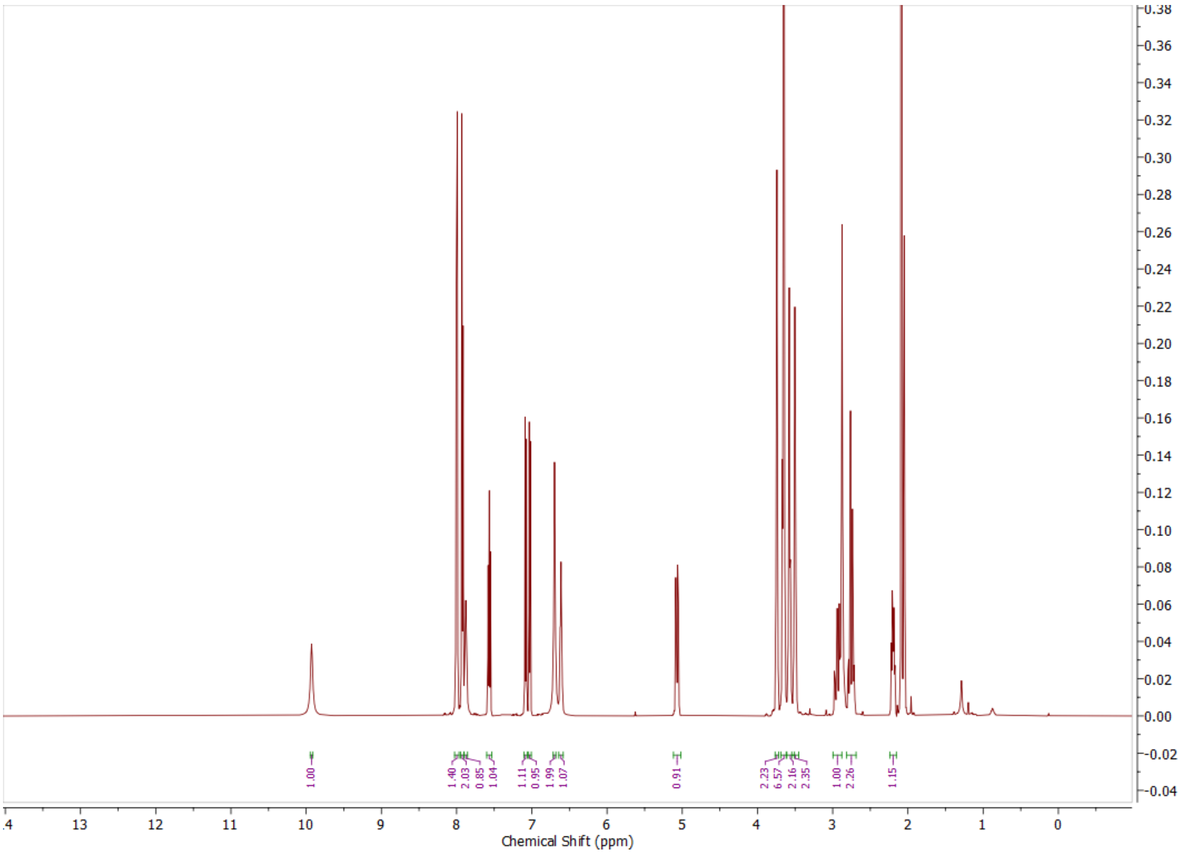

^{13}C NMR of Compound **2**:

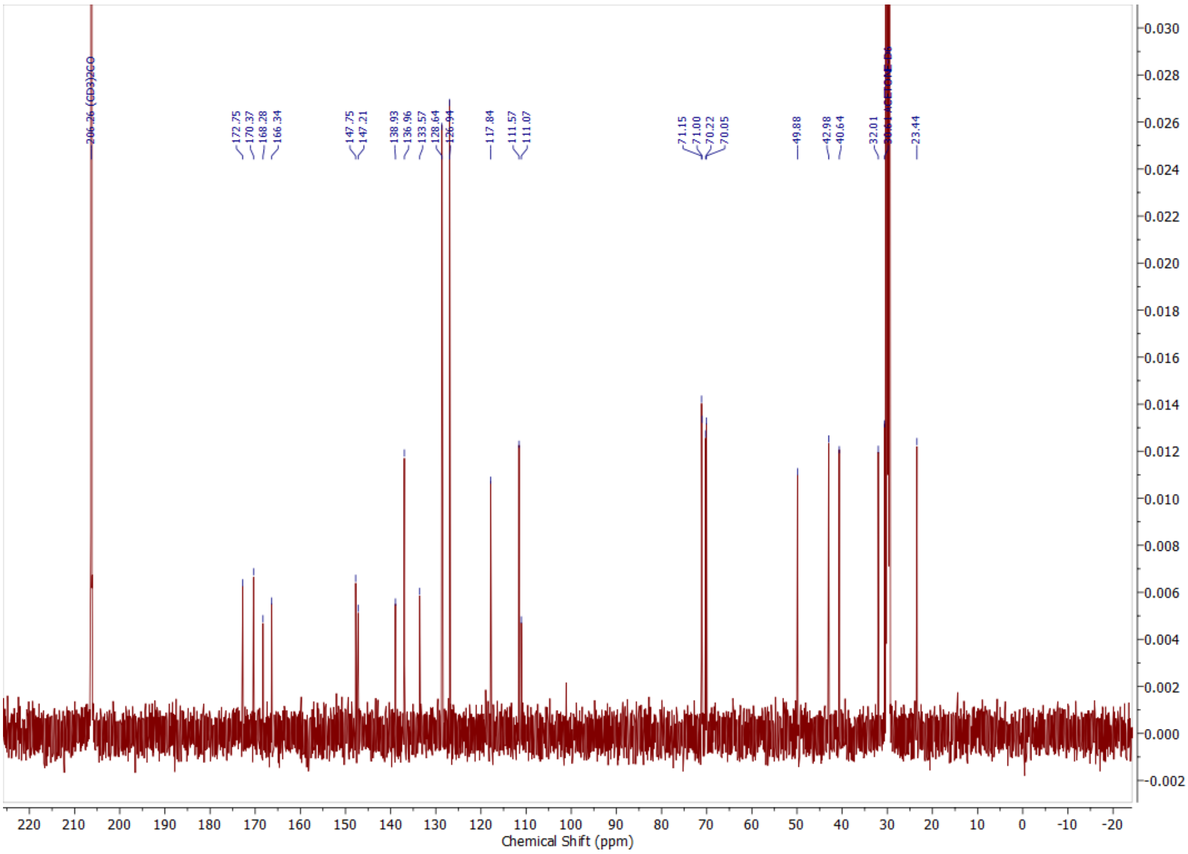

^{1}H NMR of Compound **3**:

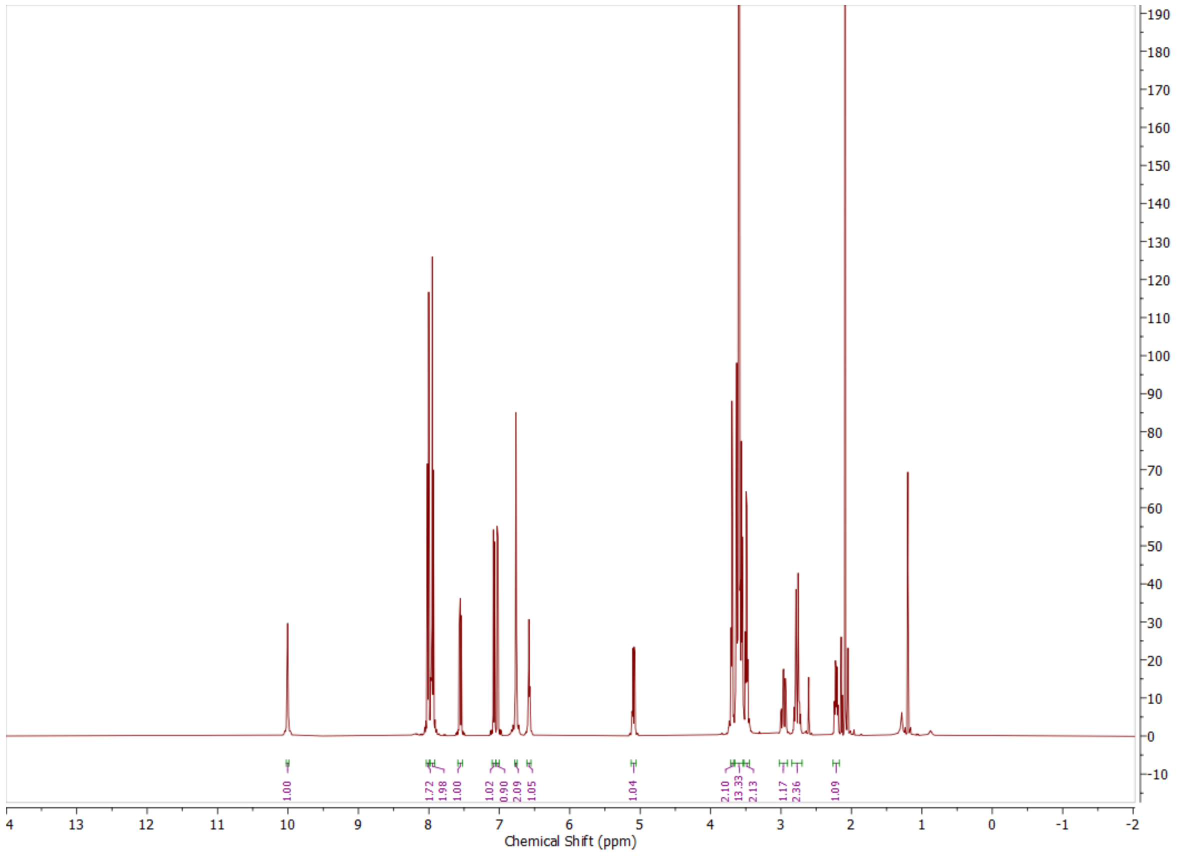

^{13}C NMR of Compound **3**:

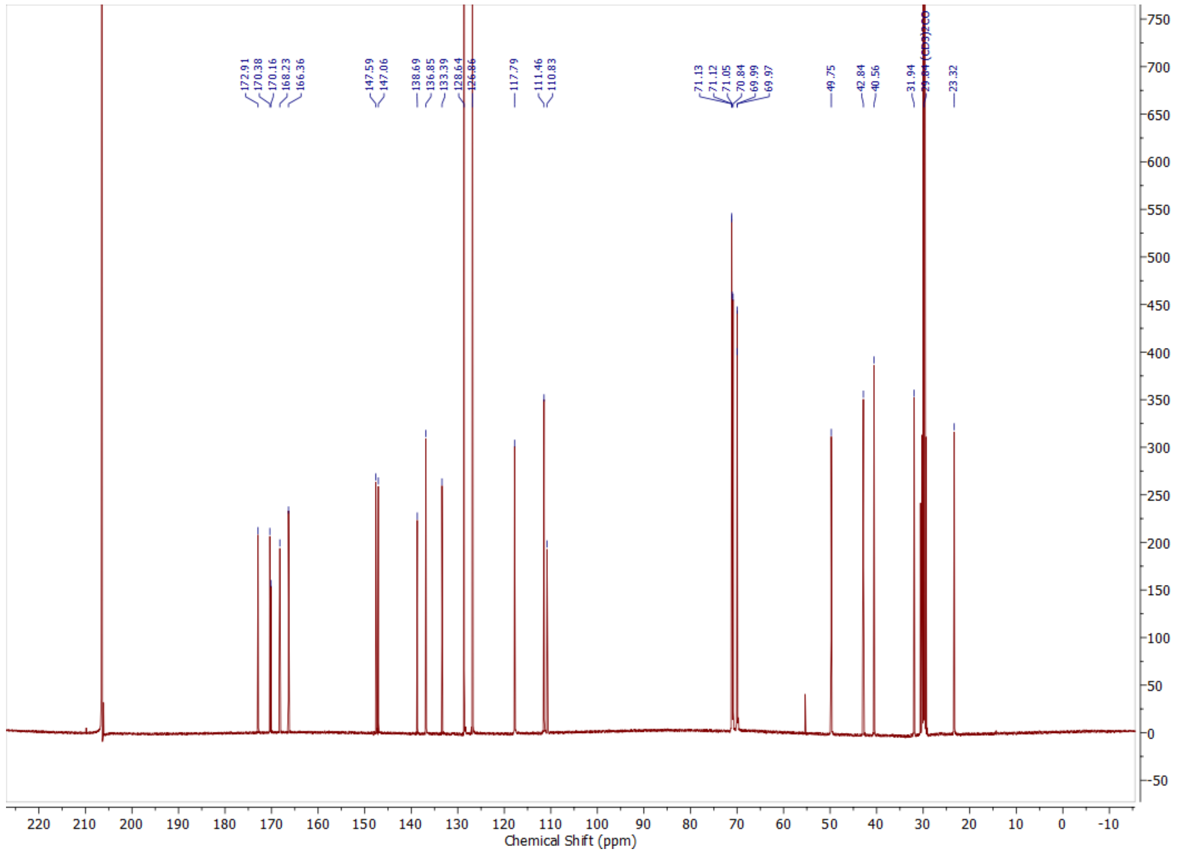

^{1}H NMR of Compound **4**:

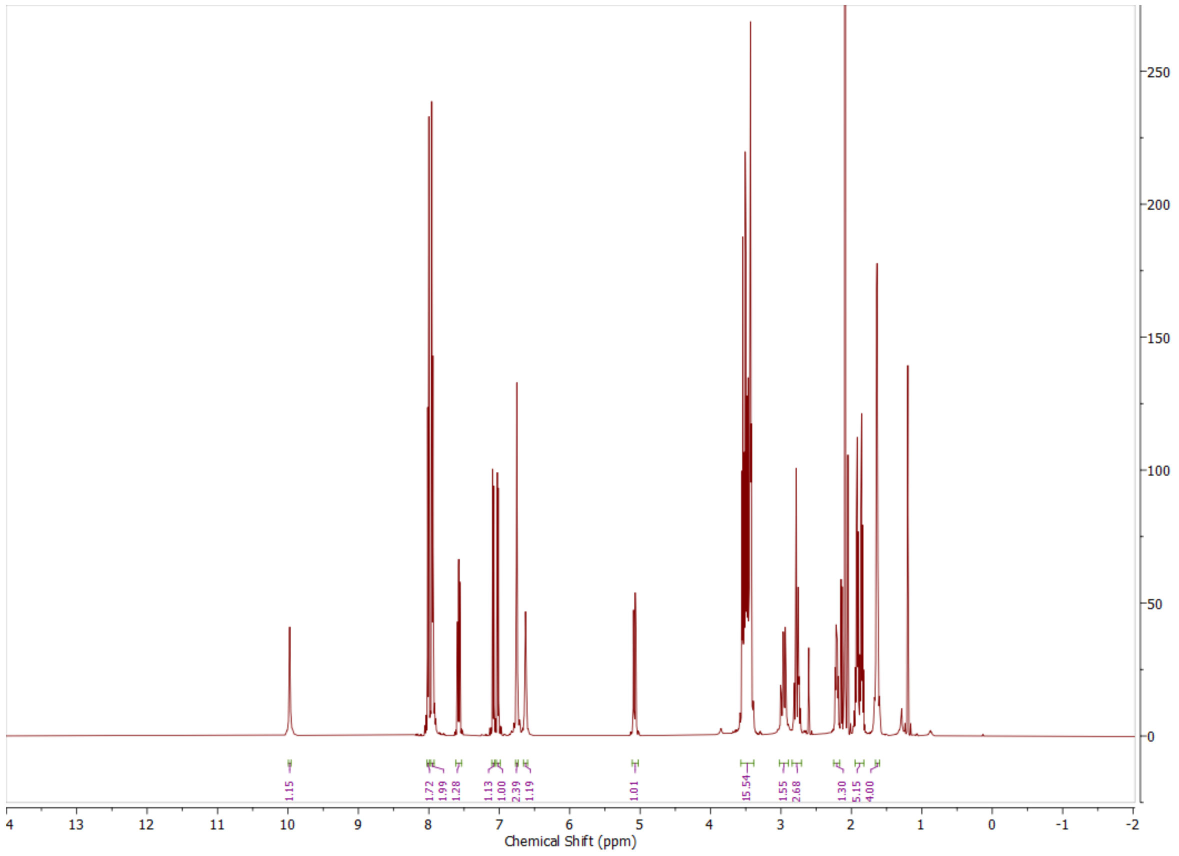

^{13}C NMR of Compound **4**:

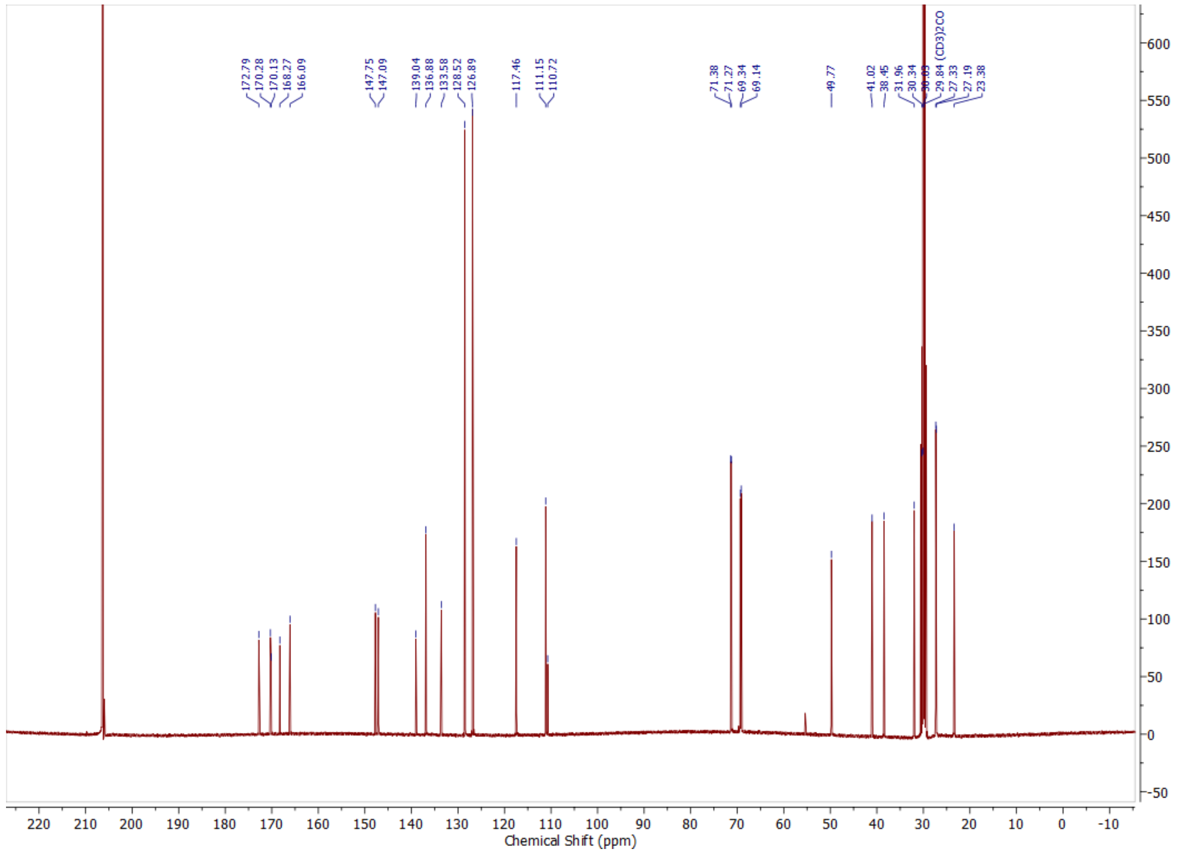

^{1}H NMR of Compound **5**:

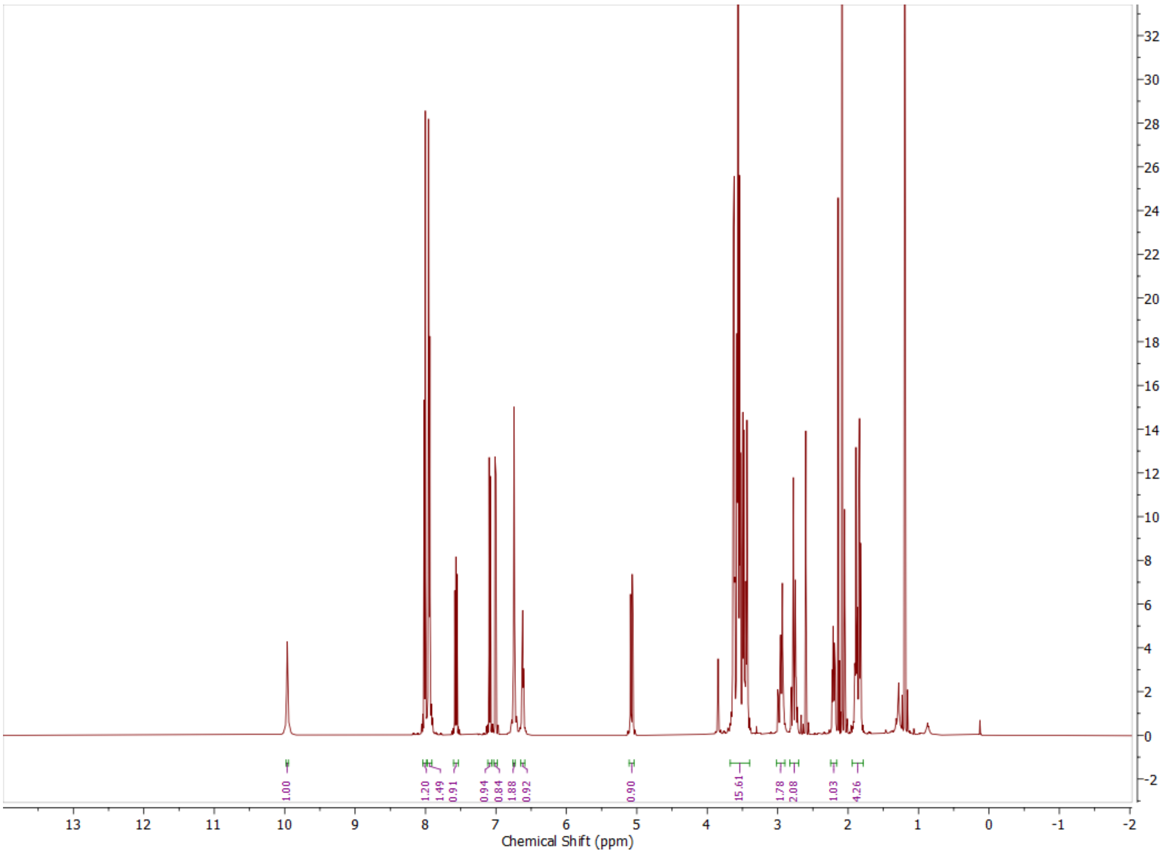

^{13}C NMR of Compound **5**:

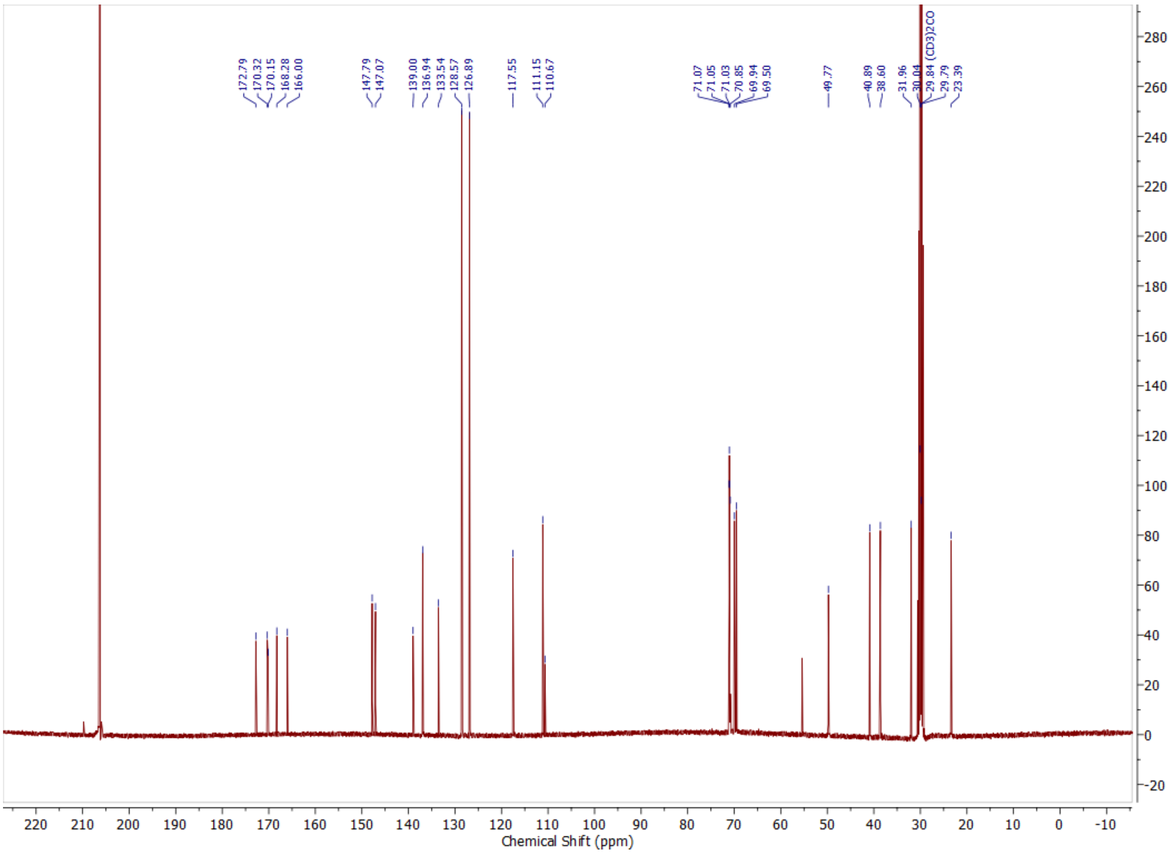

^{1}H NMR of Compound **6**:

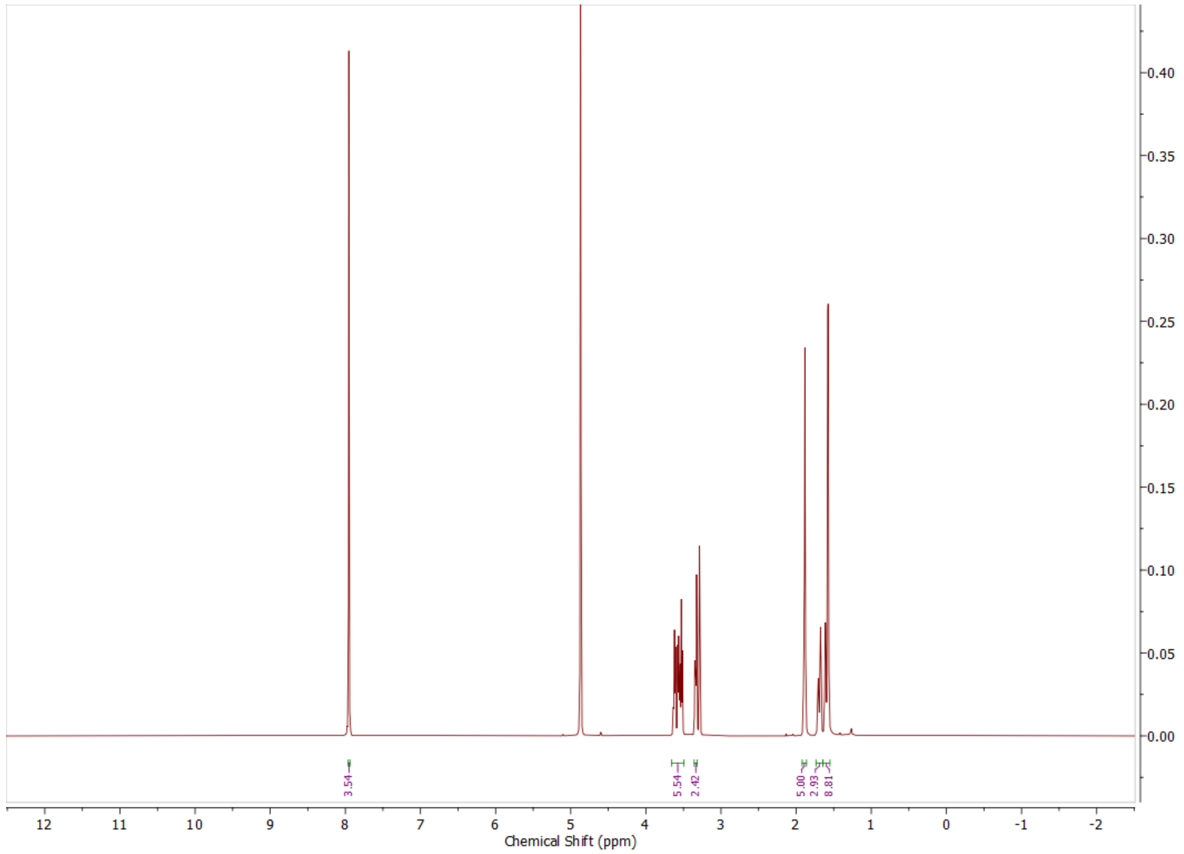

^{13}C NMR of Compound **6**:

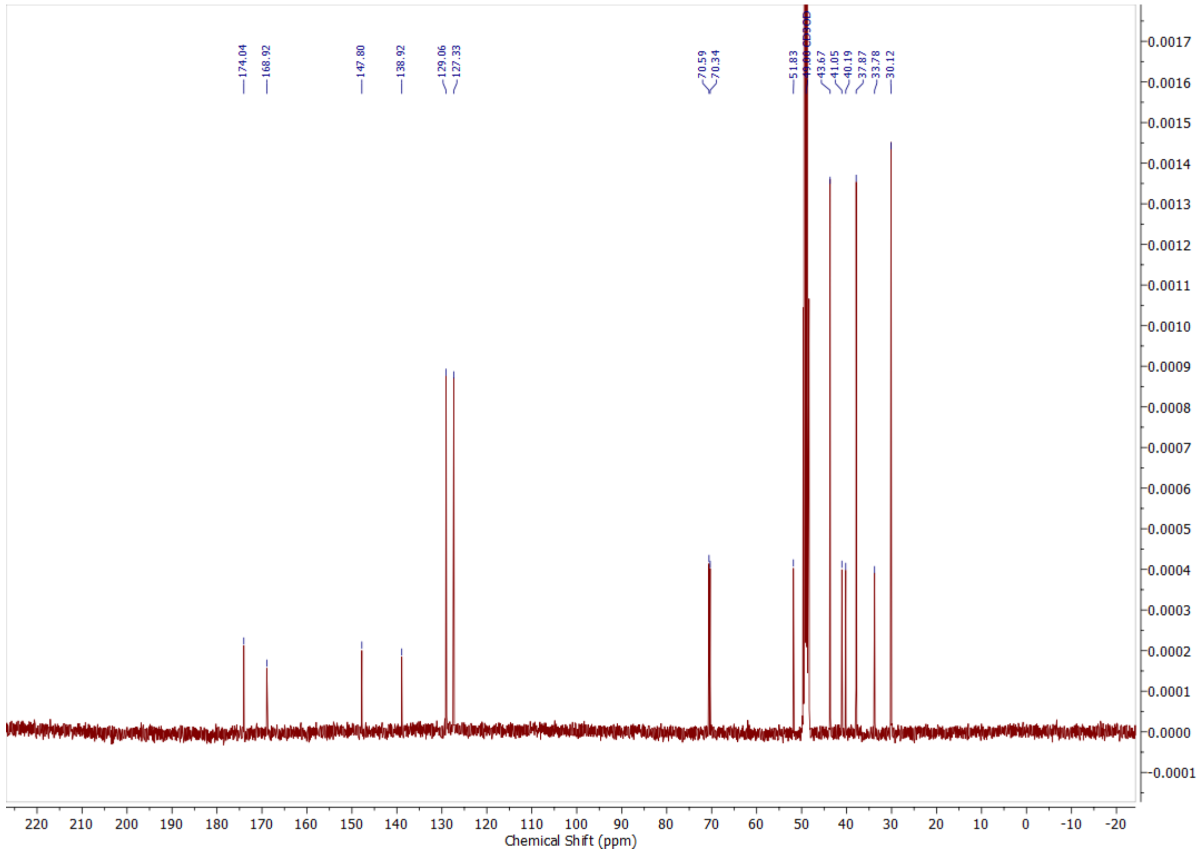

^{1}H NMR of Compound **7**:

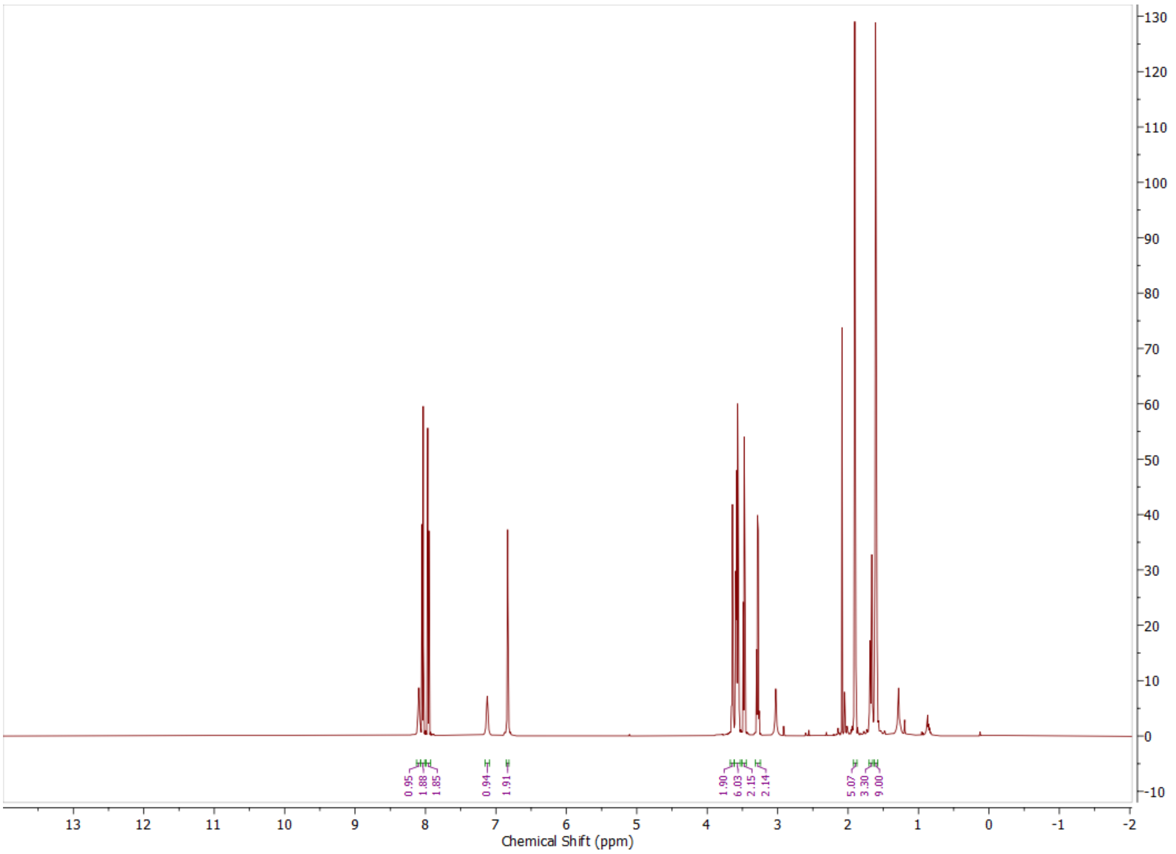

^{13}C NMR of Compound **7**:

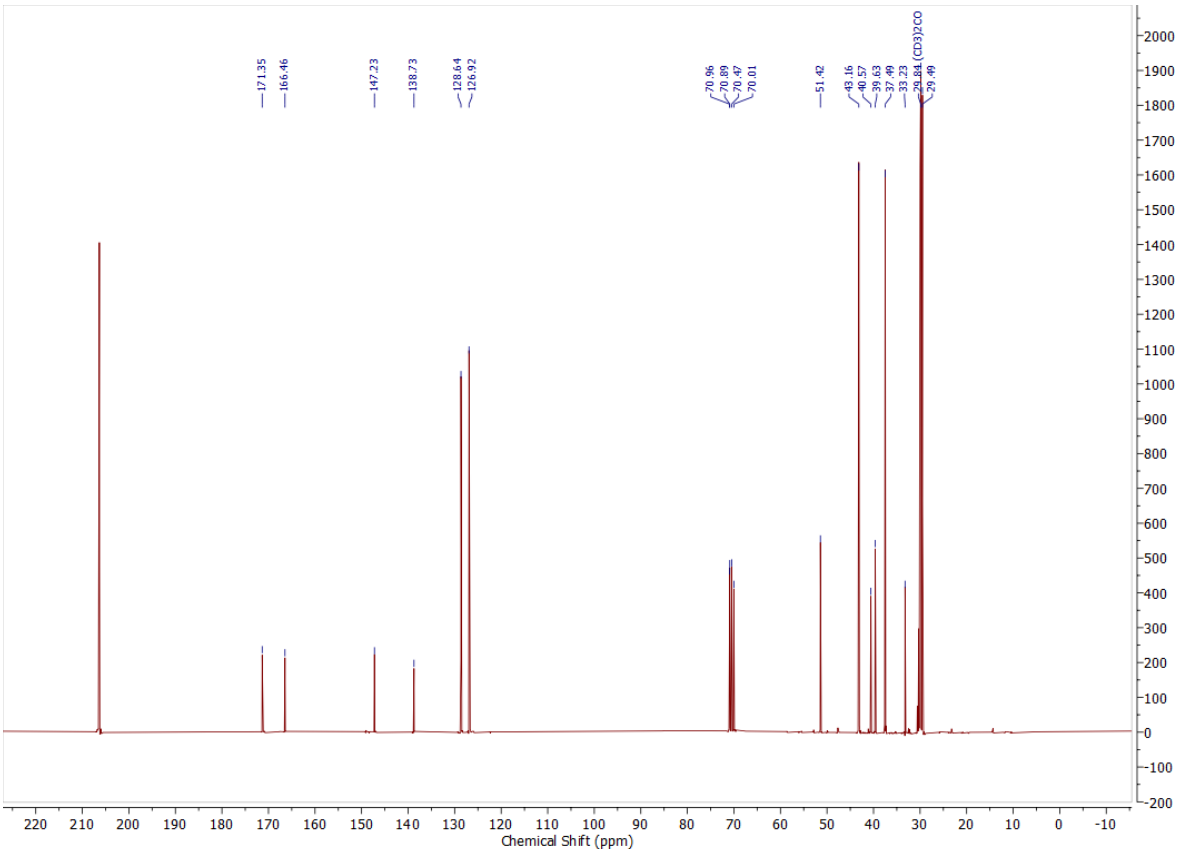

^{1}H NMR of Compound **8**:

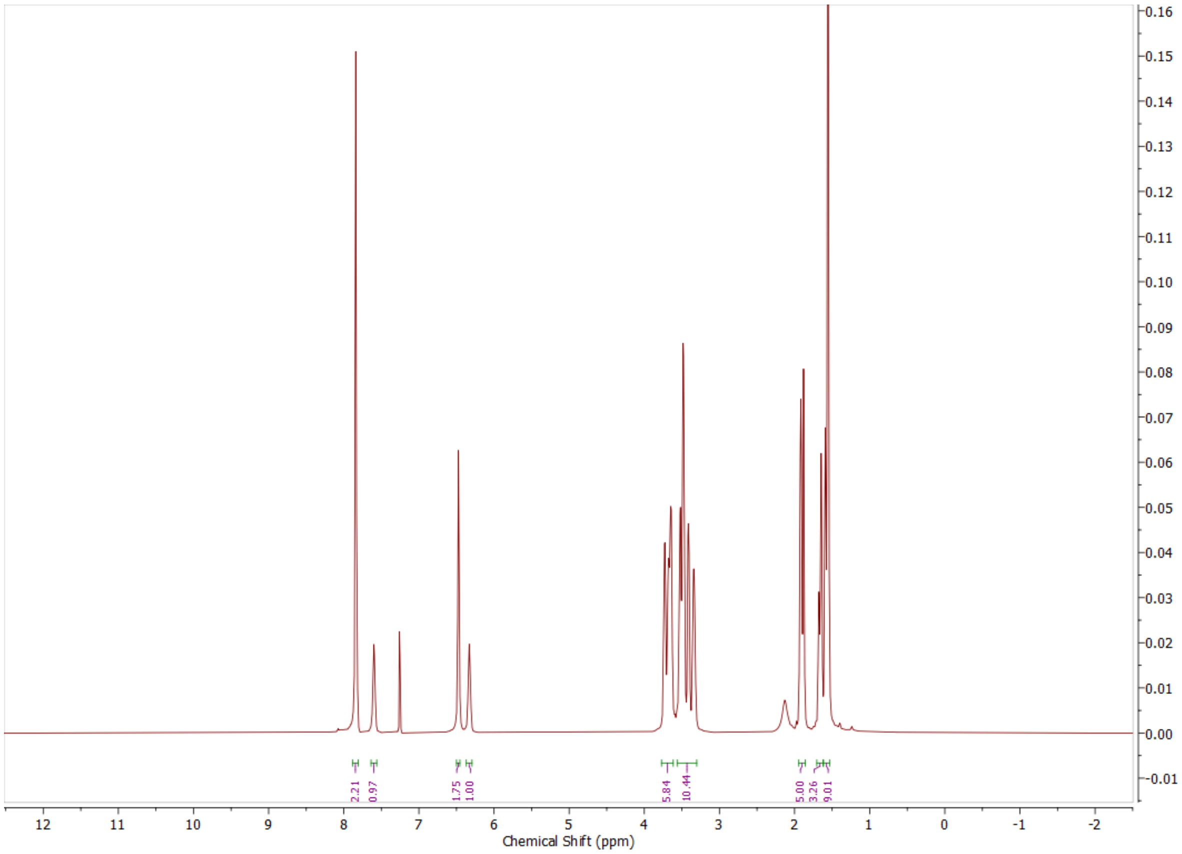

^{13}C NMR of Compound **8**:

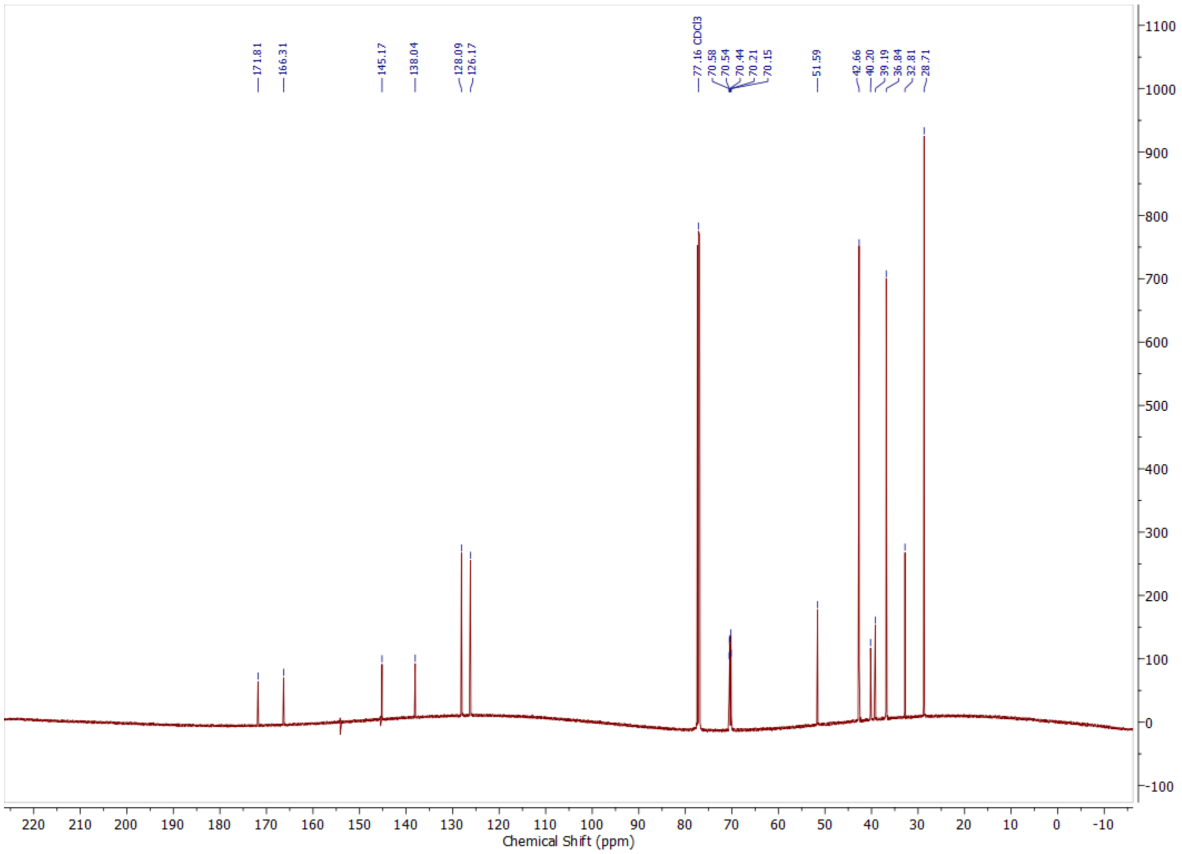

^{1}H NMR of Compound **9**:

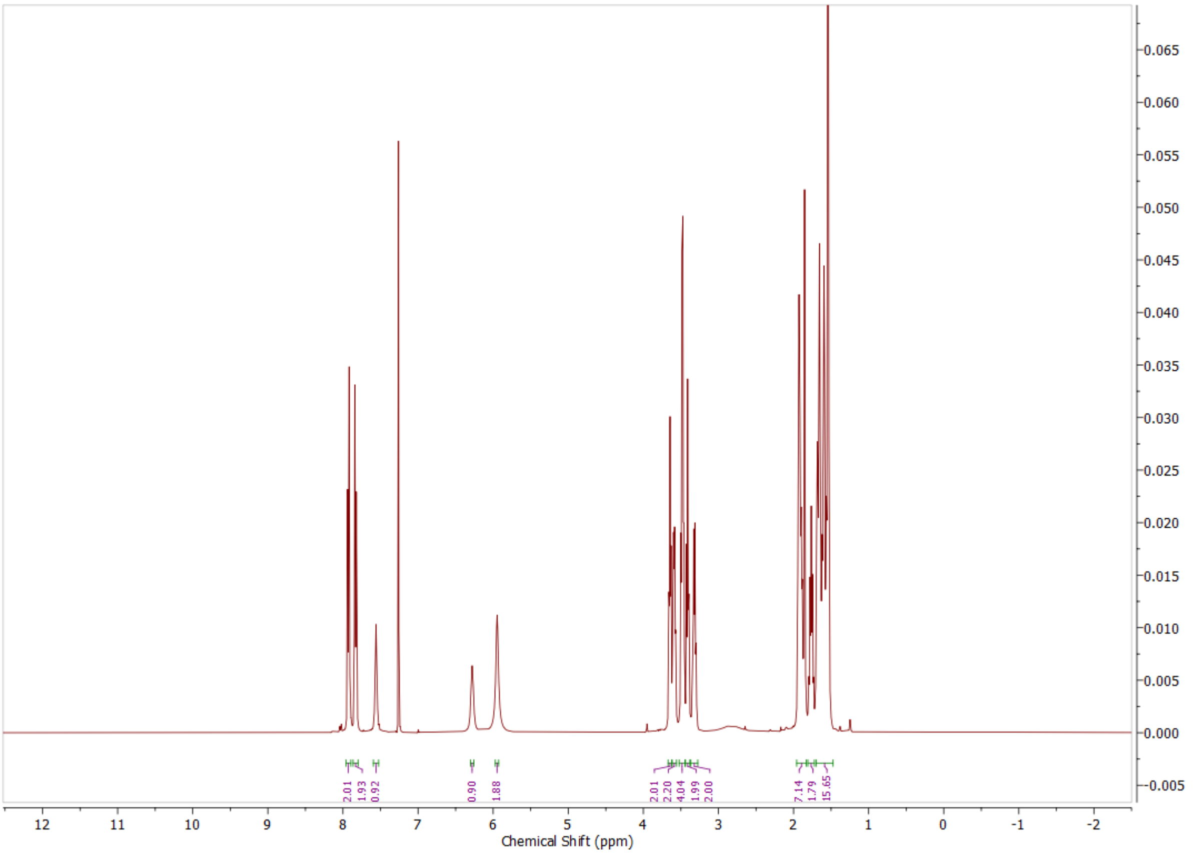

^{13}C NMR of Compound **9**:

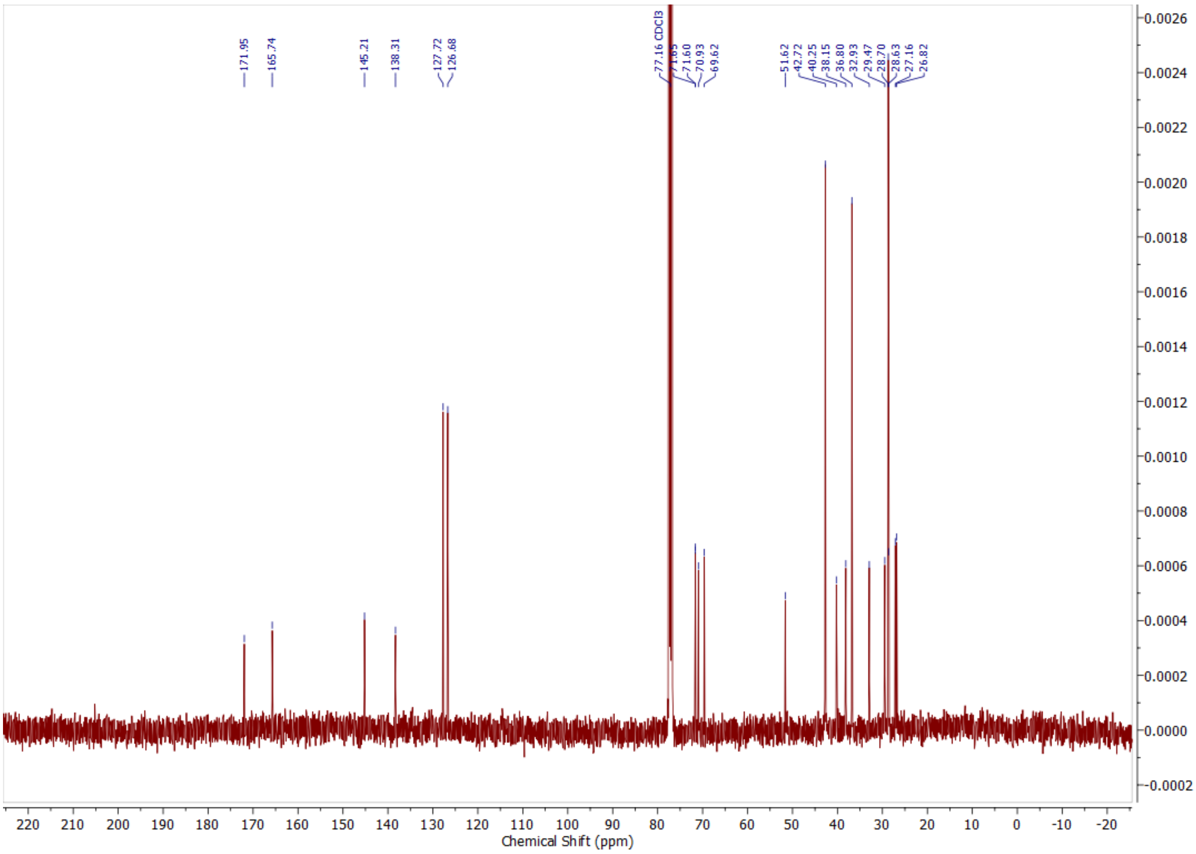

^{1}H NMR of Compound **10**:

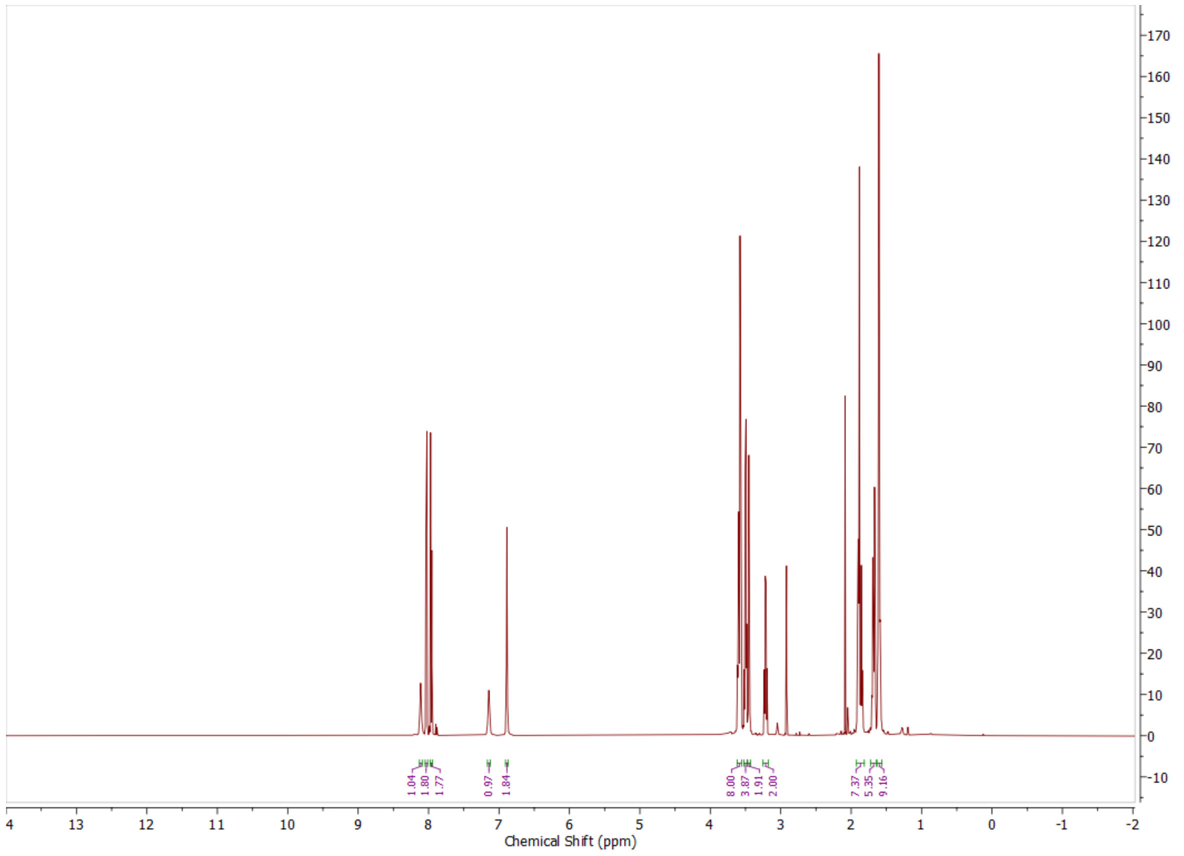

^{13}C NMR of Compound **10**:

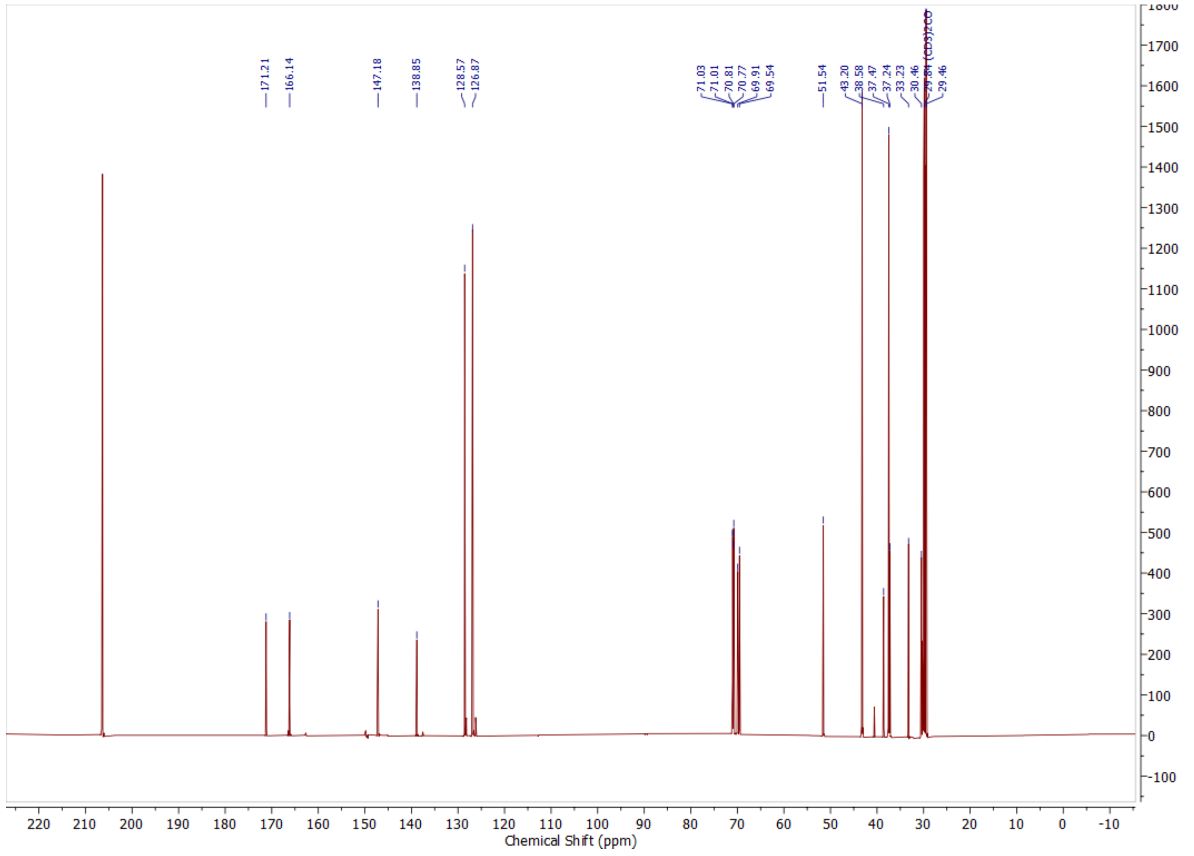

^{1}H NMR of Compound **11**:

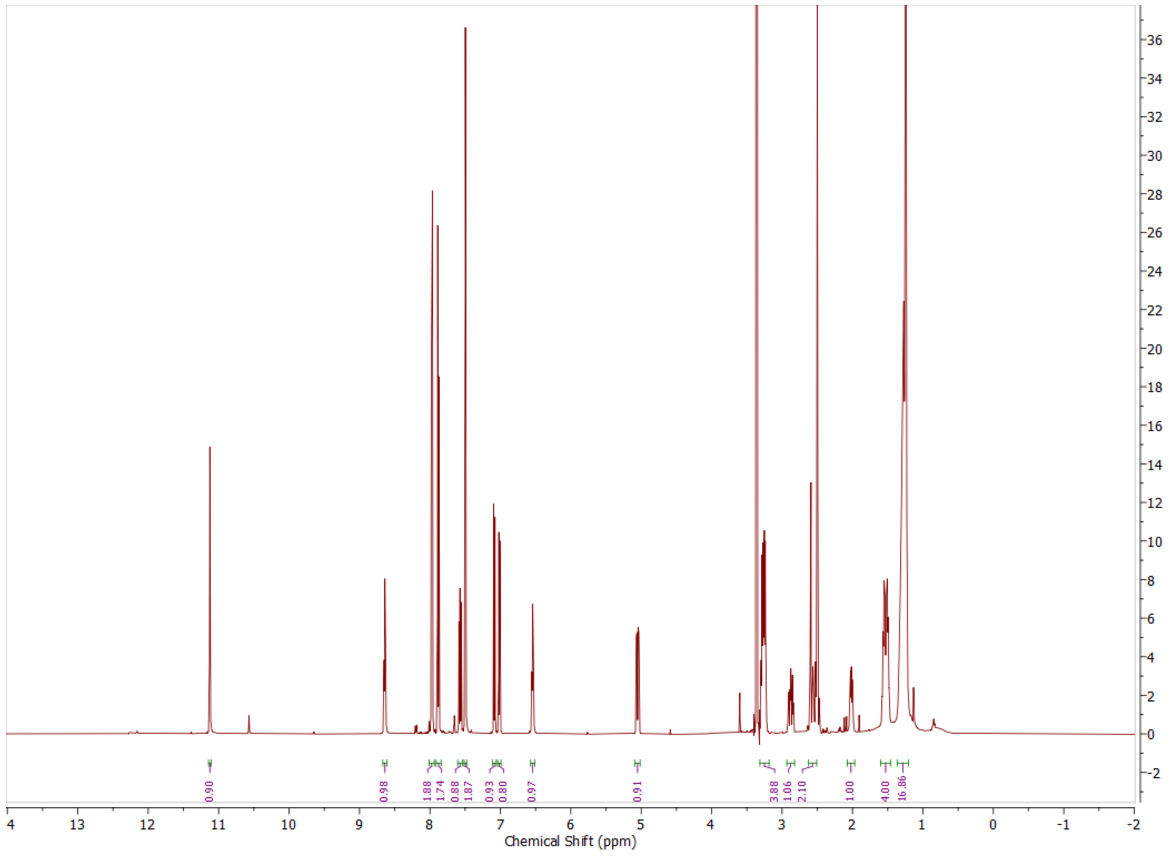

^{13}C NMR of Compound **11**:

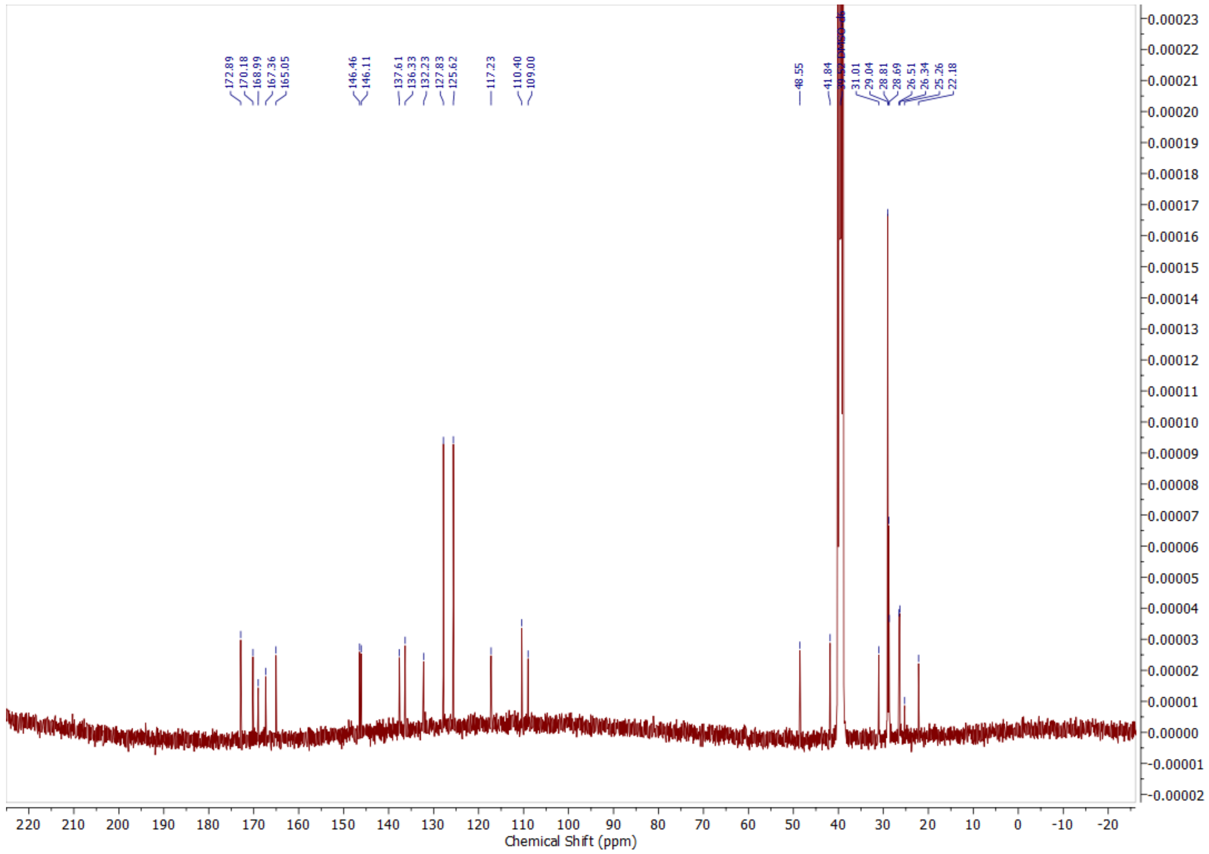

^{1}H NMR of Compound **13**:

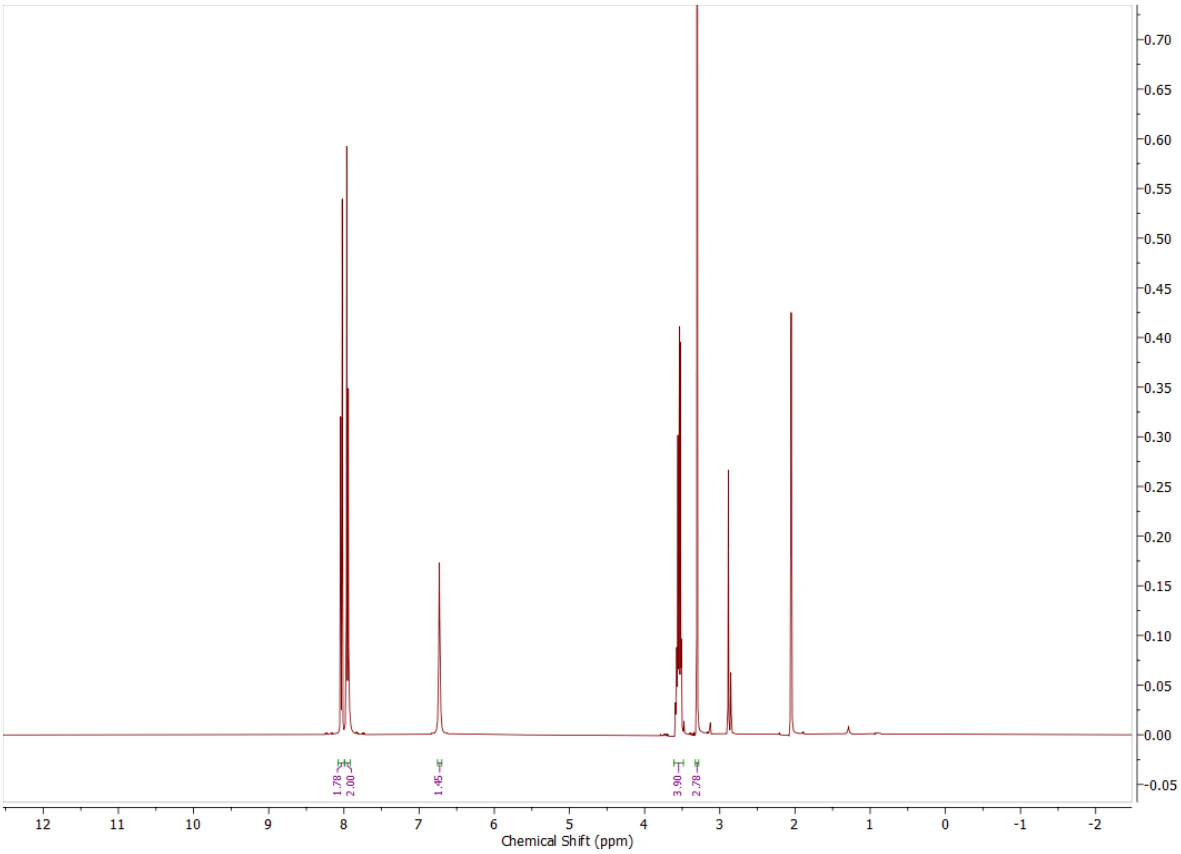

^{13}C NMR of Compound 1**3**:

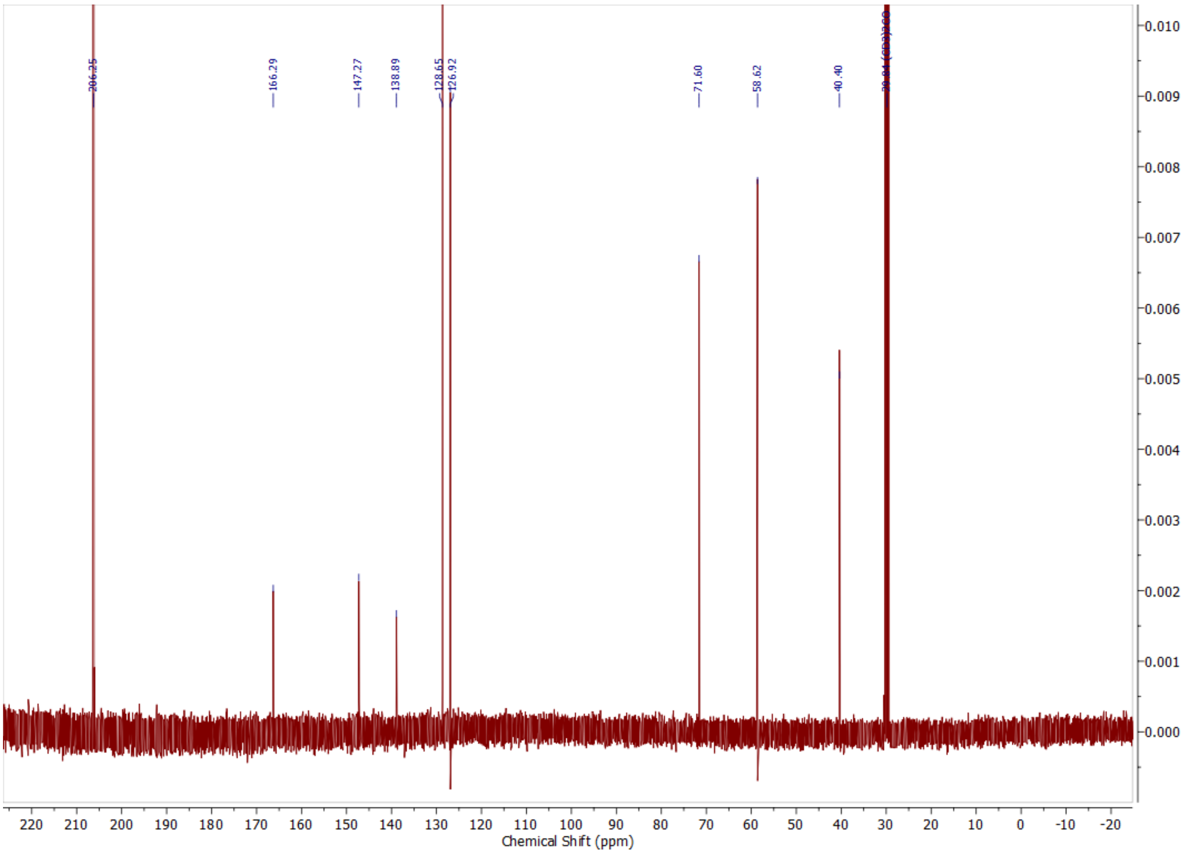

HPLC Trace for Compounds 1-11 and 13.

HPLC Trace of Compound **1**:

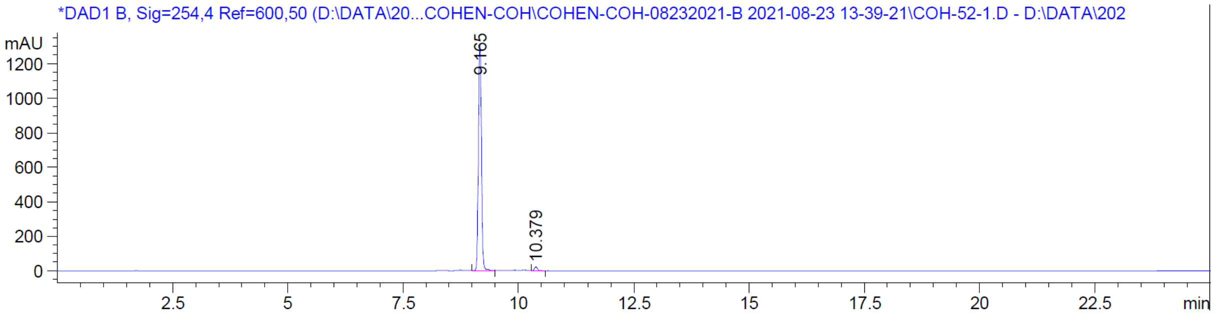

Signal 2: DAD1 B, Sig=254,4 Ref=600,50
Signal has been modified after loading from rawdata file!

Peak #	RetTime [min]	Type	Width [min]	Area [mAU*s]	Height [mAU]	Area %
1	9.165	VV	0.0706	5944.02930	1319.50488	98.1192
2	10.379	VV	0.0727	113.93610	23.42631	1.8808

Totals : 6057.96540 1342.93119

HPLC Trace of Compound **2**:

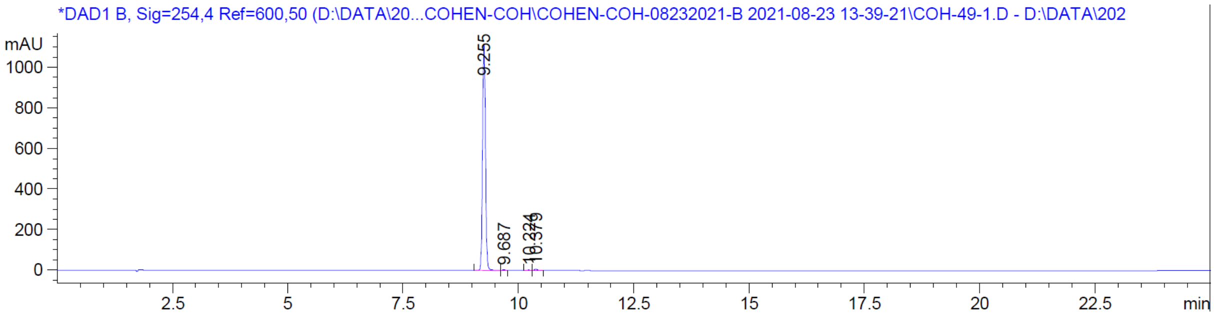

Signal 2: DAD1 B, Sig=254,4 Ref=600,50
Signal has been modified after loading from rawdata file!

Peak #	RetTime [min]	Type	Width [min]	Area [mAU*s]	Height [mAU]	Area %
1	9.255	VV	0.0674	4937.31250	1119.84387	98.4552
2	9.687	VV	0.0873	20.20191	3.30593	0.4028
3	10.224	VV	0.0919	17.86291	2.81924	0.3562
4	10.379	VV	0.0817	39.40399	7.21677	0.7858

Totals : 5014.78131 1133.18581

HPLC Trace of Compound **3**:

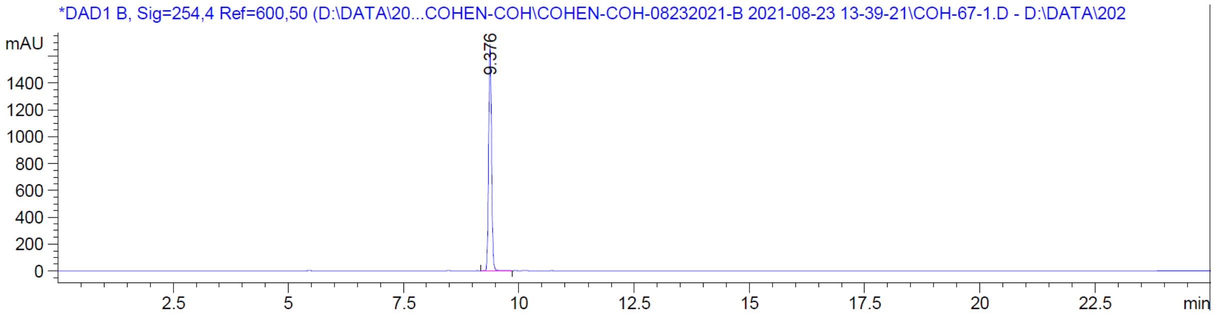

Signal 2: DAD1 B, Sig=254,4 Ref=600,50
Signal has been modified after loading from rawdata file!

Peak #	RetTime [min]	Type	Width [min]	Area [mAU*s]	Height [mAU]	Area %
1	9.376	VV	0.0695	7468.22559	1692.20911	100.0000

Totals : 7468.22559 1692.20911

HPLC Trace of Compound **4**:

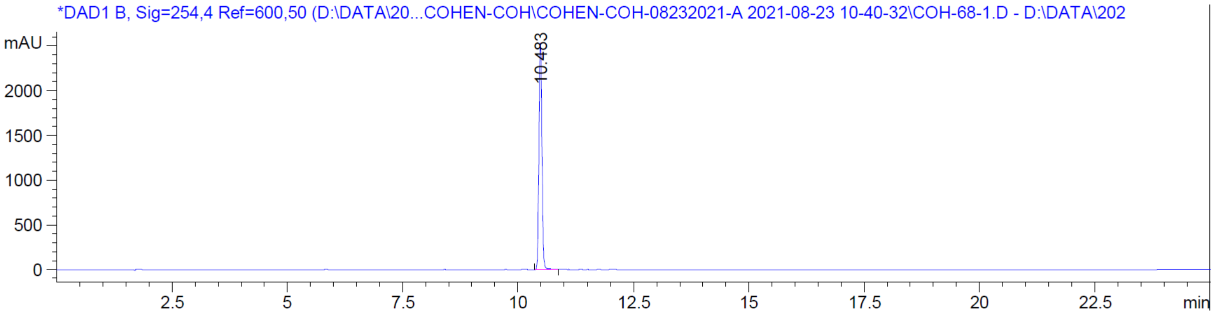

Signal 2: DAD1 B, Sig=254,4 Ref=600,50
Signal has been modified after loading from rawdata file!

Peak #	RetTime [min]	Type	Width [min]	Area [mAU*s]	Height [mAU]	Area %
1	10.483	VV	0.0736	1.16755e4	2540.59375	100.0000

Totals : 1.16755e4 2540.59375

HPLC Trace of Compound **5**:

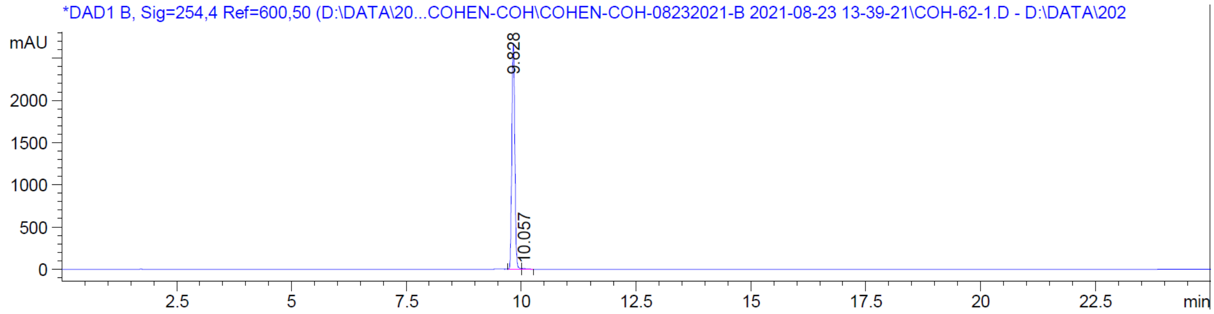

Signal 2: DAD1 B, Sig=254,4 Ref=600,50
Signal has been modified after loading from rawdata file!

Peak #	RetTime [min]	Type	Width [min]	Area [mAU*s]	Height [mAU]	Area %
1	9.828	VV	0.0731	1.22511e4	2692.91748	99.3103
2	10.057	VV	0.0976	85.08805	12.17046	0.6897

Totals : 1.23362e4 2705.08794

HPLC Trace of Compound **6**:

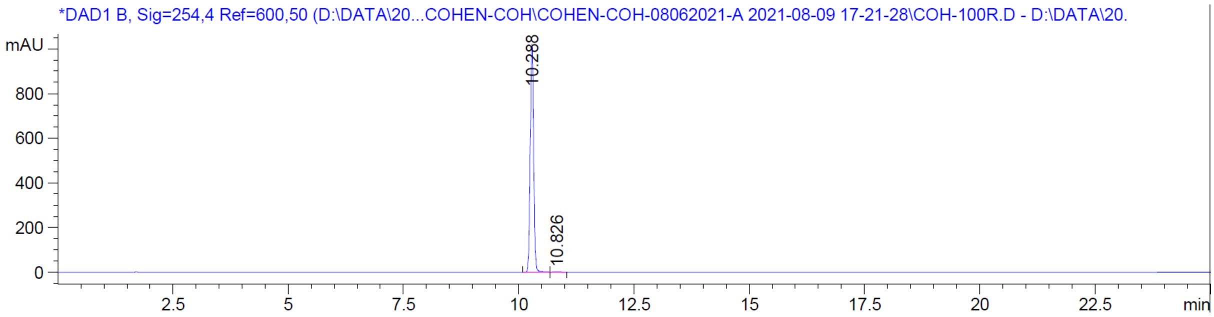

Signal 2: DAD1 B, Sig=254,4 Ref=600,50
Signal has been modified after loading from rawdata file!

Peak #	RetTime [min]	Type	Width [min]	Area [mAU*s]	Height [mAU]	Area %
1	10.288	VV	0.0824	5265.08398	1016.71509	99.4338
2	10.826	VV	0.1392	29.97951	2.98412	0.5662

Totals : 5295.06349 1019.69920

HPLC Trace of Compound **7**:

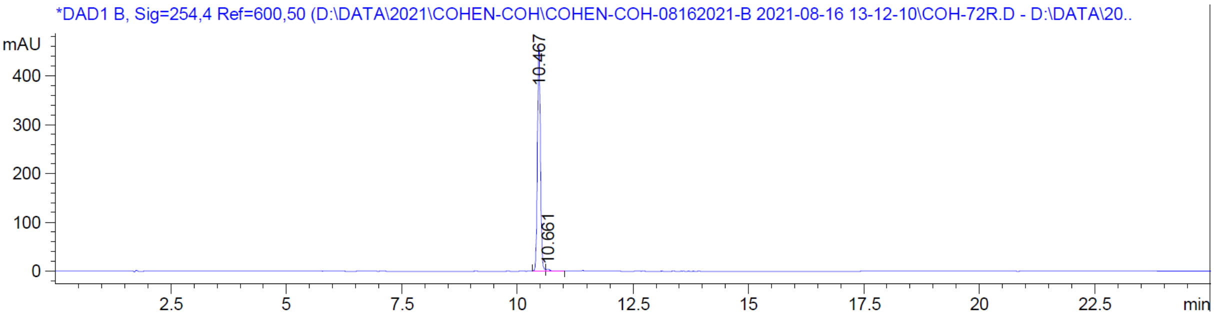

Signal 2: DAD1 B, Sig=254,4 Ref=600,50
Signal has been modified after loading from rawdata file!

Peak #	RetTime [min]	Type	Width [min]	Area [mAU*s]	Height [mAU]	Area %
1	10.467	VV	0.0708	2102.31445	464.96030	98.2363
2	10.661	VB	0.1130	37.74373	4.63511	1.7637

Totals : 2140.05819 469.59540

HPLC Trace of Compound **8**:

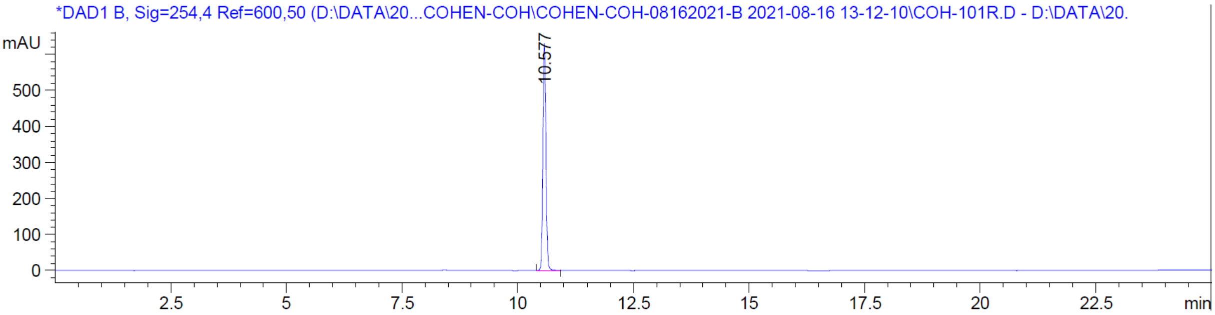

Signal 2: DAD1 B, Sig=254,4 Ref=600,50
Signal has been modified after loading from rawdata file!

Peak #	RetTime [min]	Type	Width [min]	Area [mAU*s]	Height [mAU]	Area %
1	10.577	VV	0.0714	2902.85522	633.89856	100.0000

Totals : 2902.85522 633.89856

HPLC Trace of Compound **9**:

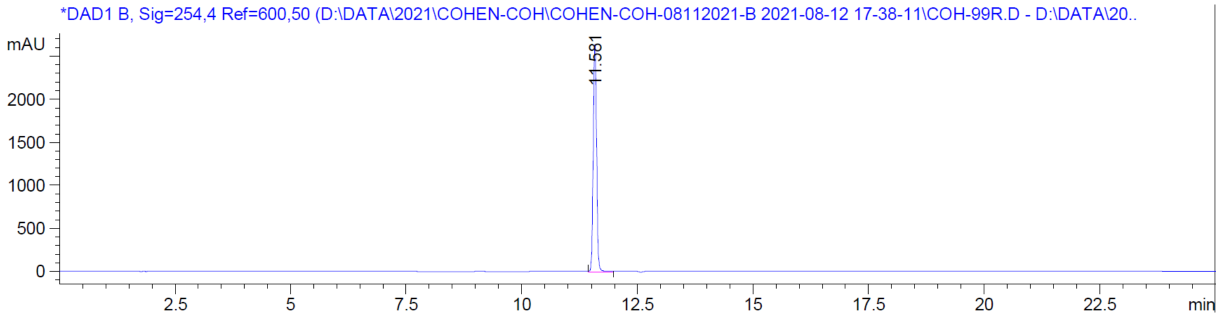

Signal 2: DAD1 B, Sig=254,4 Ref=600,50
Signal has been modified after loading from rawdata file!

Peak #	RetTime [min]	Type	Width [min]	Area [mAU*s]	Height [mAU]	Area %
1	11.581	VV	0.0802	1.36690e4	2650.58594	100.0000

Totals : 1.36690e4 2650.58594

HPLC Trace of Compound **10**:

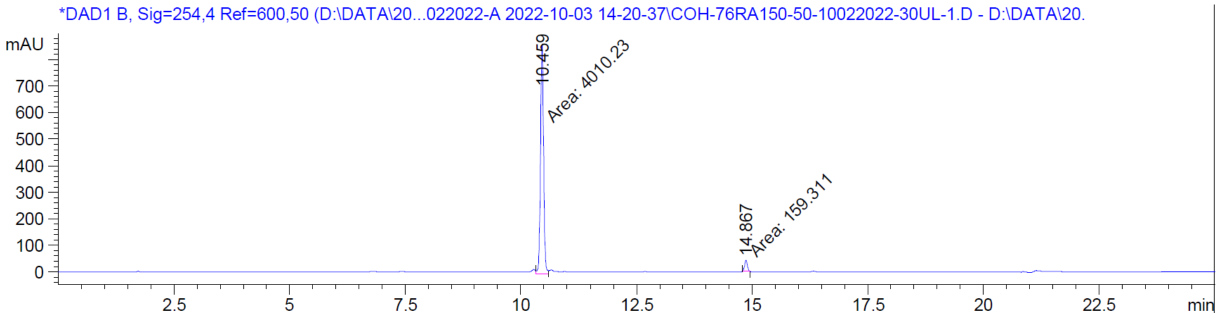

Signal 2: DAD1 B, Sig=254,4 Ref=600,50
Signal has been modified after loading from rawdata file!

Peak #	RetTime [min]	Type	Width [min]	Area [mAU*s]	Height [mAU]	Area %
1	10.459	MM	0.0772	4010.22925	866.03027	96.1792
2	14.867	MM	0.0666	159.31110	39.86028	3.8208

Totals : 4169.54034 905.89055

HPLC Trace of Compound **11**:

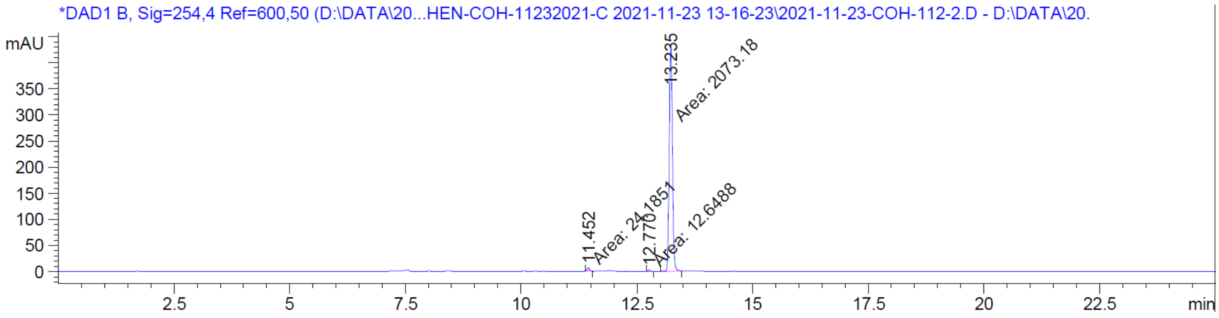

Signal 2: DAD1 B, Sig=254,4 Ref=600,50
Signal has been modified after loading from rawdata file!

Peak #	RetTime [min]	Type	Width [min]	Area [mAU*s]	Height [mAU]	Area %
1	11.452	MM	0.0671	24.18509	6.01066	1.1462
2	12.770	MM	0.0735	12.64877	2.86744	0.5995
3	13.235	MM	0.0792	2073.17578	436.38184	98.2543
Totals :				2110.00964	445.25993	

HPLC Trace of Compound **13**:

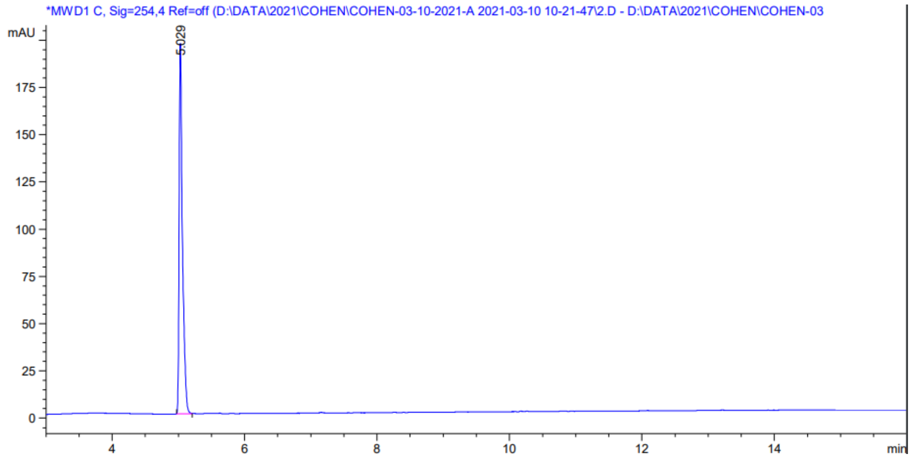

Signal 1: MWD1 C, Sig=254,4 Ref=off
Signal has been modified after loading from rawdata file!

Peak #	RetTime [min]	Type	Width [min]	Area [mAU*s]	Height [mAU]	Area %
1	5.029	BB	0.0503	678.03149	196.15242	100.0000
Totals :				678.03149	196.15242	

References

1. Drummond, M. L.; Henry, A.; Li, H.; Williams, C. I., Improved Accuracy for Modeling PROTAC-Mediated Ternary Complex Formation and Targeted Protein Degradation via New In Silico Methodologies. *J. Chem. Inf. Model.* **2020,** *60*, 5234-5254.
